# Consecutive four-component synthesis of trisubstituted 3-iodoindoles by an alkynylation–cyclization–iodination–alkylation sequence

**DOI:** 10.3762/bjoc.19.99

**Published:** 2023-09-14

**Authors:** Nadia Ledermann, Alae-Eddine Moubsit, Thomas J J Müller

**Affiliations:** 1 Heinrich-Heine Universität Düsseldorf, Institut für Organische Chemie und Makromolekulare Chemie, Universitätsstraße 1, D-40225 Düsseldorf, Germany

**Keywords:** alkynylation, catalysis, cyclization, indoles, iodination, multicomponent reactions

## Abstract

A library of 19 differently substituted 3-iodoindoles is generated by a consecutive four-component reaction starting from *ortho*-haloanilines, terminal alkynes, *N*-iodosuccinimide, and alkyl halides in yields of 11–69%. Initiated by a copper-free alkynylation, followed by a base-catalyzed cyclizive indole formation, electrophilic iodination, and finally electrophilic trapping of the intermediary indole anion with alkyl halides provides a concise one-pot synthesis of 3-iodoindoles. The latter are valuable substrates for Suzuki arylations, which are exemplified with the syntheses of four derivatives, some of them are blue emitters in solution and in the solid state, in good yield.

## Introduction

Indoles and their derived substitution patterns are omnipresent heterocyclic structural motifs in nature [1], many natural products [2–3], drugs [4–8], and dyes [9–11] and their preparation is an evergreen in organic synthesis [12–15]. Although the classical Fischer indole synthesis provides a very reliable and broadly applicable access to indole derivatives [16–18], striving for new indole syntheses is ongoing. In particular, metal-catalyzed processes for accessing indoles have become attractive alternatives over the past decades [19–24]. Besides Larock's indole synthesis employing alkyne anellation [25] and Cacchi's cyclization of *ortho*-alkynylanilines [20,22] catalytic syntheses of indoles from alkynes have become increasingly interesting [26–27]. In addition, as one-pot processes with a huge exploratory potential and diversity-oriented character, syntheses of indoles by multicomponent reactions have aroused considerable interest [28–30].

**Scheme 1:**
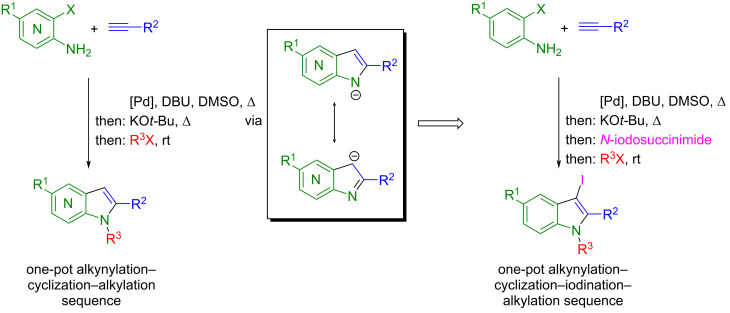
Consecutive alkynylation–cyclization–alkylation three-component synthesis and conception of a consecutive alkynylation–cyclization–iodination–alkylation four-component synthesis via an (aza)indole anion intermediate.

As part of our program to develop heterocycle syntheses based upon transition-metal catalysis [31], we disclosed an activating group-free alkynylation–cyclization sequence to (aza)indoles [32–33] that could be readily concatenated with a concluding N-alkylation of the 7-azaindole intermediate in the sense of consecutive three-component coupling–cyclization–alkylation synthesis of 1,2,5-trisubstituted 7-azaindoles [34]. Inspired by the coupling–cyclization–alkylation sequence and the stepwise Sonogashira coupling–cyclization–iodination protocol to give valuable 3-iodoindoles by Amjad and Knight [35], we reasoned that the interception by an electrophilic iodination step prior to terminal alkylation could provide a straightforward entry to trisubstituted 3-iodoindoles, which are valuable building blocks for accessing highly decorated (aza)indoles (Scheme 1). Here, we report the concise consecutive four-component synthesis of trisubstituted 3-iodoindoles.

## Results and Discussion

**Scheme 2:**
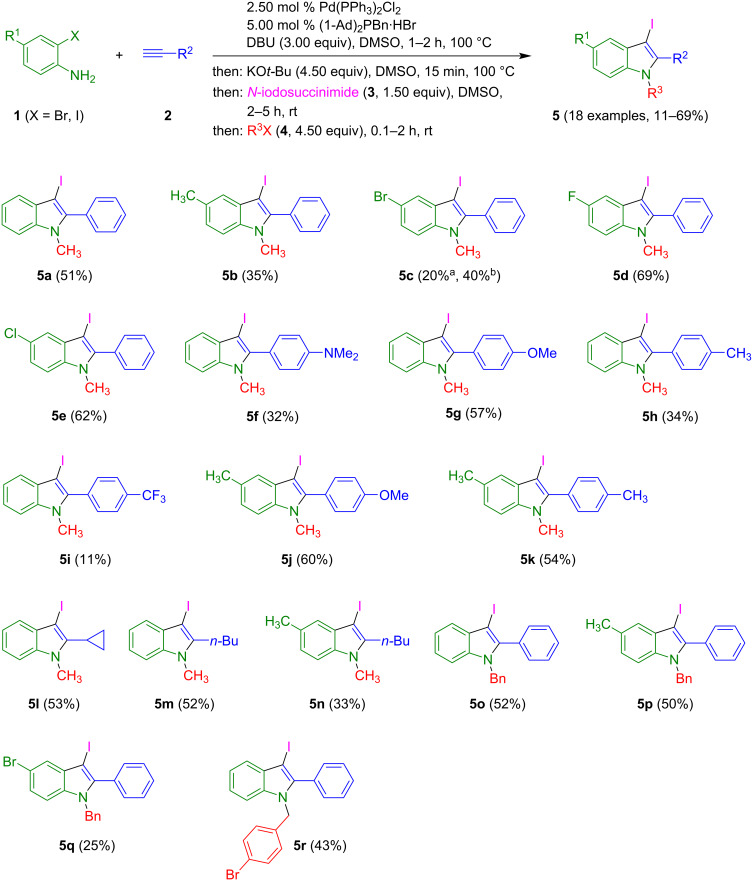
Consecutive alkynylation–cyclization–iodination–alkylation four-component synthesis of trisubstituted 3-iodoindoles (^a^with 2,4-dibromoaniline (**1c**); ^b^with 4-bromo-2-iodoaniline (**1d**)).

In our previous studies on the alkynylation–cyclization synthesis of 2-substituted (aza)indoles [32–34], we could show that the copper-free Pd-catalyzed alkynylation of 2-aminobromopyridines or 2-bromoanilines and the subsequent base-catalyzed anellation in a one-pot fashion proceeds without nitrogen protection or activation using KO*t*-Bu in DMSO as a base. Under these conditions, the formation of the terminal (aza)indole anion is the driving force (Scheme 1) [34]. As a consequence, the electrophilic trapping of this intermediate with alkyl halides provides as concise access to N-substituted (aza)indoles. As already shown for *N*-alkyl 7-azaindole formation in one case, the crucial 7-azaindole anion could be trapped with electrophilic iodine (from *N*-iodosuccinimide), resulting in a 3-iodo-7-azaindole anion, which could then be alkylated, still in a one-pot fashion [34]. Therefore, we set out to directly employ these standard conditions to the sequence of *ortho*-haloanilines **1**, terminal alkynes **2**, *N*-iodosuccinimide (**3**), and alkyl halides **4** to screen the scope of the one-pot synthesis of trisubstituted 3-iodoindoles **5** in a consecutive four-component fashion (Scheme 2).

The sequence commences with a copper-free alkynylation using DBU as a base at 100 °C. This step is followed by the addition of KO*t*-Bu and reaction at 100 °C for 15 min and subsequent reaction with *N*-iodosuccinimide (**3**) at room temperature. Finally, the reaction with alkyl halides **4** at room temperature gives the title compounds **5** in yields between 11–69% after chromatographic workup. The structures of the products were unambiguously confirmed by ^1^H and ^13^C NMR spectroscopy, as well as by mass spectrometry. Assuming that four new bonds are being formed in this one-pot process, the range of yield from 11 to 69% (after isolation) accounts for an average yield of 55–90% per bond-forming step which can be considered to be relative efficient, also because only a single terminal purification step is required. However, noteworthy, the 3-iodoindoles are sensitive to light and prolonged storage at room temperature, even under protective gas atmosphere, leads to slow decomposition. Therefore, storage at low temperature in a dark vial is strongly recommended.

**Scheme 3:**
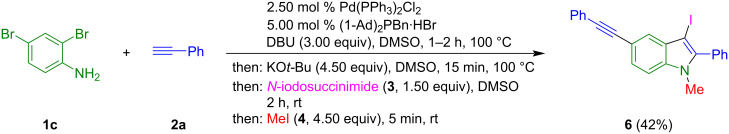
Consecutive double alkynylation–cyclization–iodination–alkylation pseudo-five-component synthesis of 5-phenylethynyl-1,2-disubstituted 3-iodoindole **6**.

Upon using 2,4-dibromoaniline (**1c**) as the substrate and an excess of phenylacetylene (**2a**), both carbon–bromine bonds are transformed in the alkynylation step affording the alkynyl-substituted 3-iodoindole **6** as product in 42% isolated yield in the sense of a pseudo-five-component reaction (Scheme 3).

**Scheme 4:**
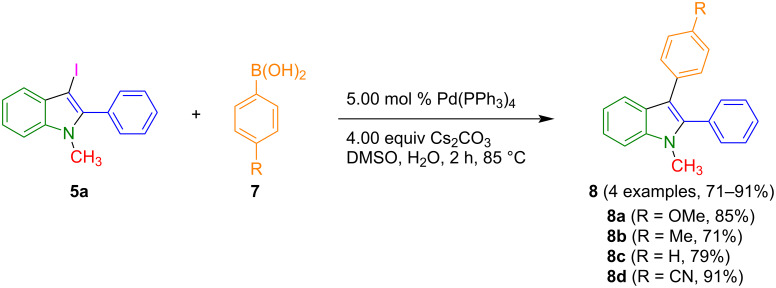
Suzuki coupling of 3-iodoindole **5a** with arylboronic acids **7** to give 1,2,3-trisubstituted indoles **8**.

Finally, the 3-iodoindole **5a** and arylboronic acids **7** were employed in a standard Suzuki protocol with cesium carbonate as a base to give rise to the formation of 1,2,3-trisubstitued indoles **8** in good yield (Scheme 4). The 1,2,3-trisubstitued indoles **8** were unambiguously confirmed by ^1^H and ^13^C NMR spectroscopy, as well as by mass spectrometry and elemental analysis.

**Figure 1:**
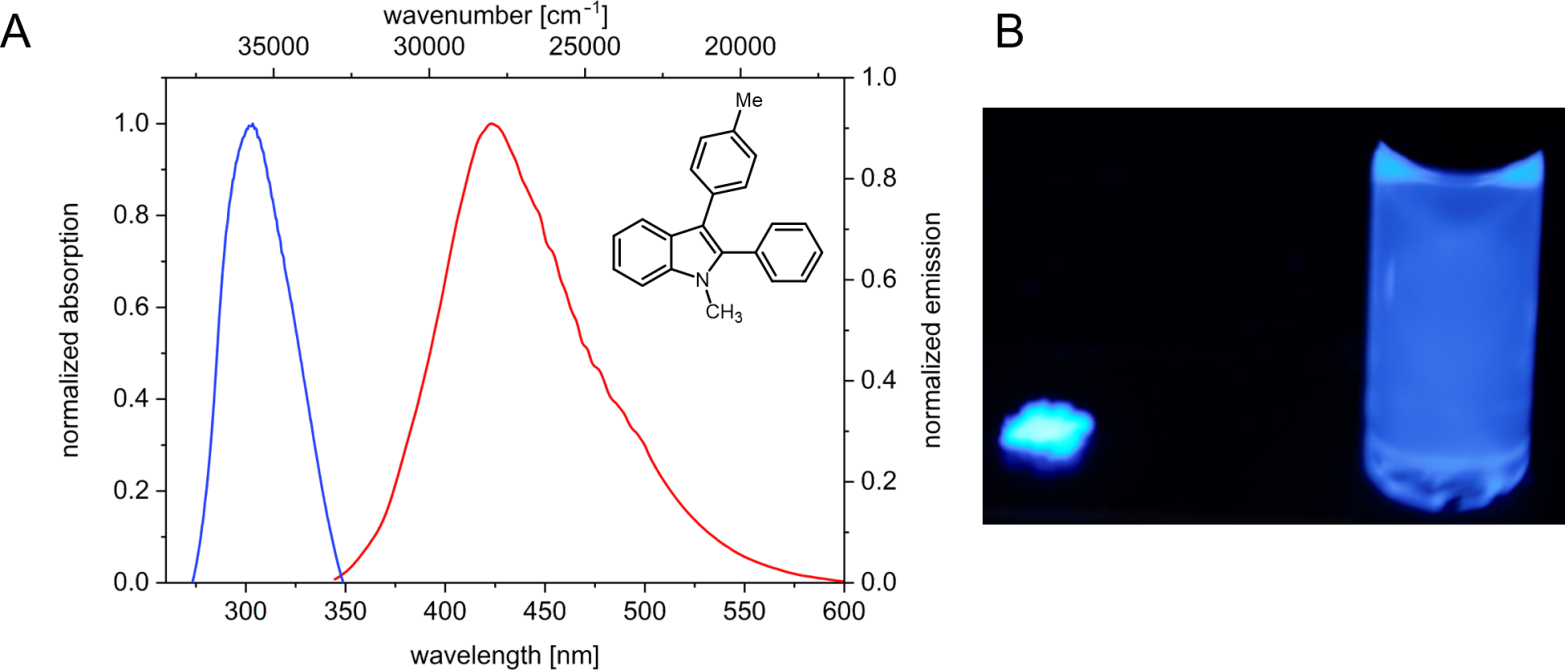
A: Absorption and emission spectra of 1-methyl-2-phenyl-3-(*p*-tolyl)-1*H*-indole (**8b**), recorded in dichloromethane, *T* = 298 K, λ_exc_ = 290 nm. B: Emission of compound **8b** in the solid state (right) and in solution (left) (UV lamp, λ_exc_ = 365 nm.

Miura et al. could show that 1-alkyl-2,3-diarylindoles constitute a class of blue-emissive indole derivatives that are accessible in two steps from indole carboxylates [36]. Our two-step approach taking advantage of a de novo formation of 3-iodoindoles with variable substitution pattern in a consecutive four-component process provides a concise access to the aforementioned class of emitters. For example the absorption maximum of indole derivative **8b** appears at 309 nm with an absorption coefficient ε = 10700 M^−1^ cm^−1^ and the emission maximum is found at 423 nm with a Stokes shift of 8700 cm^−1^ (Figure 1A). Moreover, compound **8b** emits intensively blue in both the solid state and solution (Figure 1B).

## Conclusion

In summary the indole anion intermediate resulting from a one-pot alkynylation–cyclization sequence, which has been previously shown to be efficiently trapped by carbon electrophiles to give N-substituted indoles in a consecutive three-component synthesis, can be selectively iodinated in the 3-position with *N*-iodosuccinimide prior to N-alkylation to give substituted 3-iodoindoles in a concise consecutive four-component fashion in modest to good yields. These target compounds are versatile building blocks for instance for a Suzuki coupling to give 1-alkyl-2,3-diarylindoles that can be of particular interest as indole-based blue emitters in solution and in the solid state. The expansion of this practical concise synthesis of indoles and azaindoles and their exploration as biologically active apoptosis inducers [37] and as functional blue emitters is currently underway.

## Experimental

### Consecutive four-component synthesis of 1,2-disubstituted 3-iodoindole **5d** (typical procedure)

PdCl_2_(PPh_3_)_2_ (17.4 mg, 25.0 μmol) and (1-Ad)_2_PBn·HBr (23.6 mg, 50 μmol) were placed in an oven-dried Schlenk tube with magnetic stirring bar under nitrogen. Then, 2-iodo-4-fluoroaniline (**1e**, 237 mg, 1.00 mmol), phenylacetylene (**2a**, 122 mg, 1.20 mmol), DBU (457 mg, 3.00 mmol), and DMSO (1.50 mL) were added under nitrogen. The reaction mixture was heated at 100 °C (oil bath) for 2 h. After cooling to room temperature, potassium *tert*-butoxide (505 mg, 4.50 mmol) and DMSO (1.50 mL) were added to the reaction mixture and heated to 100 °C (oil bath) for 15 min. After cooling to room temperature, *N*-iodosuccinimide (**3**, 338 mg, 1.50 mmol) and DMSO (1.00 mL) were added and the mixture stirred at room temperature for a further 2 to 5 h (monitored by TLC). Then, methyl iodide (**4a**, 639 mg, 4.50 mmol) was added and the reaction mixture was stirred at room temperature for 0.1 to 2 h (monitored by TLC). Deionized water (20 mL) was added to the reaction mixture and the aqueous phase was extracted with dichloromethane (3 × 20 mL). The combined organic phases were dried (anhydrous sodium sulfate), filtered, and the solvent was then removed under vacuo. The residue was purified by chromatography on silica gel (*n*-hexane/ethyl acetate 100:1 to 5:1) to give pure compound **5d** (243 mg, 69%) as a beige solid. Mp 118.2 °C; *R*_f_ 0.50 (*n*-hexane/ethyl acetate 10:1); ^1^H NMR (600 MHz, CDCl_3_) δ 3.68 (s, 3H), 7.02–7.07 (m, 1H), 7.17–7.20 (m, 1H), 7.23–7.26 (m, 1H), 7.46–7.54 (m, 5H); ^13^C NMR (150 MHz, CDCl_3_) δ 1.9 (C_quat_), 32.4 (CH_3_), 106.7 (CH), 110.8 (CH), 111.5 (CH), 128.6 (C_quat_), 129.3, 130.9, 131.5 (C_quat_), 134.5 (C_quat_), 143.5 (C_quat_), 160.0 (C_quat_); IR (cm^−1^) ν̃: 604 (w), 619 (w), 662 (w), 689 (s), 733 (m), 756 (s), 789 (m), 860 (w), 907 (w), 934 (w), 957 (w), 997 (w), 1028 (w), 1051 (w), 1074 (w), 1107 (w), 1132 (m), 1165 (w), 1206 (m), 1238 (w), 1261 (w), 1275 (w), 1292 (w), 1315 (w), 1352 (w), 1406 (w), 1445 (w), 1456 (m), 1472 (w), 1541 (w), 1585 (w), 1622 (w), 1865 (w), 2853 (w), 2924 (w), 2961 (w), 3032 (w), 3957 (w), 3103 (w); EIMS (70 eV) *m*/*z* (%): 351 ([M]^+^, 2), 211 (C_14_H_10_FN^+^, 100), 149 (19), 106 (12), 71 (10), 57 (22); HRMS (*m*/*z*): [M + H]^+^ calcd for C_15_H_12_FIN, 351.9993; found, 351.9831.

### Consecutive pseudo-five-component synthesis of 3-iodo-1-methyl-2-phenyl-5-(phenylethynyl)-1*H*-indole (**6**)

PdCl_2_(PPh_3_)_2_ (17.4 mg, 25.0 µmol) and (1-Ad)_2_PBn·HBr (23.6 mg, 50 μmol) were placed in an oven-dried Schlenk tube with magnetic stirring bar under nitrogen. Then, 2,4-dibromoaniline (**1c**, 254 mg, 1.00 mmol), phenylacetylene (**2a**, 245 mg, 2.40 mmol), DBU (457 mg, 3.00 mmol), and 1.50 mL DMSO were added and flushed with nitrogen. The reaction mixture was heated at 100 °C until complete conversion of the starting material (via TLC control). Potassium *tert*-butoxide (505 mg, 4.50 mmol) and 1.50 mL DMSO were then added and the reaction mixture was stirred for an additional 15 min. After cooling the reaction mixture to room temperature, NIS (338 mg, 1.50 mmol) and 1.00 mL DMSO were added. After complete conversion (via TLC control), methyl iodide (639 mg, 4.50 mmol) was added and also stirred at room temperature for 5 min. Water was added to the mixture and the aqueous phase was extracted with dichloromethane. The combined organic phases were dried with anhydrous sodium sulfate, filtered, and the solvent was removed under reduced pressure. The residue was purified by chromatography on silica gel (*n*-hexane/ethyl acetate 20:1 to 5:1) to give compound **6** (184 mg, 42%) as a colorless solid. Mp 204.5 °C; *R*_f_ 0.35 (*n*-hexane/ethyl acetate 10:1); ^1^H NMR (300 MHz, CDCl_3_) δ 3.69 (s, 3H), 7.27–7.30 (m, 1H), 7.31–7.39 (m, 3H), 7.46–7.56 (m, 6H), 7.57–7.60 (m, 2H), 7.72–7.75 (m, 1H); ^13^C NMR (75 MHz, CDCl_3_) δ 32.3 (CH_3_), 59.2 (C_quat_), 87.9 (C_quat_), 90.8 (C_quat_), 110.1 (CH), 115.5 (C_quat_), 123.9 (C_quat_), 125.4 (CH), 126.6 (CH), 128.0 (CH), 128.5 (CH), 128.6 (CH), 129.1 (CH), 130.5 (C_quat_), 131.0 (CH), 131.4 (C_quat_), 131.7 (CH), 137.6 (C_quat_), 142.9 (C_quat_); IR (cm^−1^) ν̃: 611 (m), 621 (w), 664 (w), 679 (m), 691 (s), 702 (s), 754 (s), 787 (m), 806 (s), 870 (w), 916 (w), 970 (w), 1022 (w), 1069 (w), 1103 (w), 1148 (w), 1179 (w), 1209 (w), 1229 (w), 1277 (w), 1298 (w), 1337 (w), 1364 (w), 1431 (w), 1441 (w), 1473 (w), 1493 (w), 1595 (w), 1873 (w), 1954 (w), 2029 (w), 2810 (w), 2847 (w), 2893 (w); EIMS (70 eV), *m*/*z* (%): 433 ([M], 100), 304 ([M − I], 38), 227 (11), 153 (29); Anal. calcd for C_23_H_16_IN: C, 63.76; H, 3.72; N, 3.23; found: C, 63.81; H, 3.74; N, 2.96.

### Synthesis of 1,2,3-trisubstituted indole **8b** (typical procedure)

3-Iodoindole **5a** (167 mg, 0.50 mmol), (*p*-tolyl)boronic acid (**7b**, 204 mg, 1.50 mmol), Pd(PPh_3_)_4_ (28.9 mg, 25.0 μmol), and cesium carbonate (652 mg, 2.00 mmol) were placed in an oven-dried Schlenk tube with magnetic stirring bar under nitrogen. Under nitrogen DMSO (5.00 mL) and deionized water (0.80 mL) were added and the reaction mixture was heated to 85 °C (oil bath) for 2 h. Then, deionized water (20 mL) was added to the reaction mixture and the aqueous phase was extracted with dichloromethane (3 × 20 mL). The combined organic phases were dried (anhydrous sodium sulfate), filtered, and the solvent was removed under vacuo. The residue was purified by chromatography on silica gel (*n*-hexane/ethyl acetate 20:1 to 5:1) to give compound **8b** (105 mg, 71%) as a colorless solid. Mp 150.8 °C (lit.: 157 °C [38]); *R*_f_ 0.58 (*n*-hexane/ethyl acetate 10:1); ^1^H NMR (300 MHz, CDCl_3_) δ 2.24 (s, 3H), 3.58 (s, 3H), 6.98–7.02 (m, 2H), 7.08–7.14 (m, 3H), 7.40–7.17 (m, 7H), 7.68–7.73 (m, 1H); ^13^C NMR (75 MHz, CDCl_3_) δ 21.3 (CH_3_), 31.1 (CH_3_), 109.7 (CH), 115.1 (C_quat_), 119.8 (CH), 120.2 (CH), 122.2 (CH), 127.2 (C_quat_), 128.1 (CH), 128.5 (CH), 129.1 (CH), 129.8 (CH), 131.3 (CH), 132.2 (C_quat_), 132.2 (C_quat_), 135.1 (C_quat_), 137.4 (C_quat_), 137.6 (C_quat_); IR (cm^−1^) ν̃: 698 (s), 721 (m), 741 (s), 783 (w), 810 (m), 918 (w), 939 (m), 1005 (w), 1020 (m), 1037 (w), 1072 (w), 1088 (m), 1117 (w), 1138 (w), 1227 (w), 1261 (w), 1306 (w), 1329 (m), 1368 (m), 1391 (w), 1414 (w), 1431 (w), 1460 (m), 1499 (w), 1512 (w), 1545 (w), 1607 (w), 2855 (w), 2914 (w), 2961 (w), 3013 (w), 3026 (w), 3053 (w); EIMS (70 eV), *m*/*z* (%): 297 ([M], 1), 208 ([C_15_H_12_N], 100), 180 (12), 165 (12); Anal. calcd for C_22_H_19_N: C, 88.85; H, 6.44; N, 4.71; found: C, 88.65; H, 6.29; N, 4.52.

## Supporting Information

File 1 Experimental details of the synthesis and analytical data of compounds **5**, **6**, and **8**, ^1^H and ^13^C NMR spectra of compounds **5**, **6**, and **8**.
