# Supplementary material for: Consecutive four-component synthesis of trisubstituted 3-iodoindoles by an alkynylation–cyclization–iodination–alkylation sequence
Source: Beilstein J Org Chem. 2023 Sep 14;19:1379–85. doi: 10.3762/bjoc.19.99 (PMC10509542; doi:10.3762/bjoc.19.99)
Supplement: File 1 — Experimental details of the synthesis and analytical data of compounds 5, 6, and 8, 1H and 13C NMR spectra of compounds 5, 6, and 8. [file Beilstein_J_Org_Chem-19-1379-s001.pdf]

BEILSTEIN JOURNAL OF ORGANIC CHEMISTRY

Supporting Information

for

Consecutive four-component synthesis of trisubstituted 3-iodoindoles by an alkynylation–cyclization–iodination–alkylation sequence

Nadia Ledermann, Alae-Eddine Moubsit and Thomas J. J. Müller

Beilstein J. Org. Chem. **2023,** *19,* 1379–1385. doi:10.3762/bjoc.19.99

Experimental details of the synthesis and analytical data of compounds 5, 6, and 8, ^{1}H and ^{13}C NMR spectra of compounds 5, 6, and 8

License and Terms: This is a supporting information file under the terms of the Creative Commons Attribution License (https://creativecommons.org/licenses/by/4.0). Please note that the reuse, redistribution and reproduction in particular requires that the author(s) and source are credited and that individual graphics may be subject to special legal provisions.
The license is subject to the *Beilstein Journal of Organic Chemistry* terms and conditions: (https://www.beilstein-journals.org/bjoc/terms)

Table of contents

1. General considerations S3

2. Starting materials S4

2.1. Di(adamantan-1-yl)(benzyl)phosphonium bromide S4

2.2. 2-Arylethynes **2** S4

3. Synthetic procedure and analytical data S5

3.1. General procedure GP for the synthesis of 1,2-disubstituted 3-iodoindoles **5** S5

3.1.1 3-Iodo-1-methyl-2-phenyl-1*H*-indole (**5a**) S6

3.1.2 3-Iodo-1,5-dimethyl-2-phenyl-1*H*-indole (**5b**). S7

3.1.3 5-Bromo-3-iodo-1-methyl-2-phenyl-1*H*-indole (**5c**) S7

3.1.4 5-Fluoro-3-iodo-1-methyl-2-phenyl-1*H*-indole (**5d**) S8

3.1.5 5-Chloro-3-iodo-1-methyl-2-phenyl-1*H*-indole (**5e**) S9

3.1.6 4-(3-Iodo-1-methyl-1*H*-indol-2-yl)-*N*,*N*-dimethylaniline (**5f**) S9

3.1.7 3-Iodo-2-(4-methoxyphenyl)-1-methyl-1*H*-indole (**5g**) S10

3.1.8 3-Iodo-1-methyl-2-(*p*-tolyl)-1*H*-indole (**5h**) S10

3.1.9 3-Iodo-1-methyl-2-(4-(trifluormethyl)phenyl)-1*H*-indole (**5i**) S11

3.1.10 3-Iodo-2-(4-methoxyphenyl)-1,5-dimethyl-1*H*-indole (**5j**) S11

3.1.11 3-Iodo-1,5-dimethyl-2-(*p*-tolyl)-1*H*-indole (**5k**) S12

3.1.12 2-Cyclopropyl-3-iodo-1-methyl-1*H*-indole (**5l**) S12

3.1.13 2-Butyl-3-iodo-1-methyl-1*H*-indole (**5m**) S13

3.1.14 2-Butyl-3-iodo-1,5-dimethyl-1*H*-indole (**5n**) S13

3.1.15 1-Benzyl-3-iodo-2-phenyl-1*H*-indole (**5o**) S14

3.1.16 1-Benzyl-3-iodo-5-methyl-2-phenyl-1*H*-indole (**5p**) S15

3.1.17 1-Benzyl-5-bromo-3-iodo-2-phenyl-1*H*-indole (**5q**) S15

3.1.18 1-(4-Bromobenzyl)-3-iodo-2-phenyl-1*H*-indole (**5r**) S16

3.2. Synthesis of 3-iodo-1-methyl-2-phenyl-5-(phenylethynyl)-1*H*-indole (**6**) S17

3.3. General procedure GP for the synthesis of 1,2,3-trisubstituted indoles **8** S17

3.3.1. 3-(4-Methoxyphenyl)-1-methyl-2-phenyl-1*H*-indole (**8a**) S18

3.3.2. 1-Methyl-2-phenyl-3-(*p*-tolyl)-1*H*-indole (**8b**) S19

3.3.3. 1-Methyl-2,3-diphenyl-1*H*-indole (**8c**)[39] S19

3.3.4. 4-(1-Methyl-2-phenyl-1*H*-indol-3-yl)benzonitrile (**8d**) S20

4. ^{1}H and ^{13}C NMR spectra of compounds 5, 6, and 8 .... S21

4.1. 3-Iodo-1-methyl-2-phenyl-1*H*-indole (**5a**) S21

4.2. 3-Iodo-1,5-dimethyl-2-phenyl-1*H*-indole (**5b**) S22

4.3. 5-Bromo-3-iodo-1-methyl-2-phenyl-1*H*-indole (**5c**) S23

4.4. 5-Fluoro-3-iodo-1-methyl-2-phenyl-1*H*-indole (**5d**) S24

4.5. 5-Chloro-3-iodo-1-methyl-2-phenyl-1*H*-indole (**5e**) S25

4.6. 4-(3-Iodo-1-methyl-1*H*-indol-2-yl)-*N*,*N*-dimethylaniline (**5f**) S26

4.7. 3-Iodo-2-(4-methoxyphenyl)-1-methyl-1*H*-indole (**5g**) S27

4.8. 3-Iodo-1-methyl-2-(*p*-tolyl)-1*H*-indole (**5h**) S28
4.9. 3-Iodo-1-methyl-2-(4-(trifluormethyl)phenyl)-1*H*-indole (**5i**) S29
4.10. 3-Iodo-2-(4-methoxyphenyl)-1,5-dimethyl-1*H*-indole (**5j**) S30
4.11. 3-Iodo-1,5-dimethyl-2-(*p*-tolyl)-1*H*-indole (**5k**) S31
4.12. 2-Cyclopropyl-3-iodo-1-methyl-1*H*-indole (**5l**) S32
4.13. 2-Butyl-3-iodo-1-methyl-1*H*-indole (**5m**) S33
4.14. 2-Butyl-3-iodo-1,5-dimethyl-1*H*-indole (**5n**) S34
4.15. 1-Benzyl-3-iodo-2-phenyl-1*H*-indole (**5o**) S35
4.16. 1-Benzyl-3-iodo-5-methyl-2-phenyl-1*H*-indole (**5p**) S36
4.17. 1-Benzyl-5-bromo-3-iodo-2-phenyl-1*H*-indole (**5q**) S37
4.18. 1-(4-Bromobenzyl)-3-iodo-2-phenyl-1*H*-indole (**5r**) S38
4.19. Synthesis of 3-Iod-1-methyl-2-phenyl-5-(phenylethynyl)-1*H*-indole (**6**) S39
4.20. 3-(4-Methoxyphenyl)-1-methyl-2-phenyl-1*H*-indole (**8a**) S40
4.21. 1-Methyl-2-phenyl-3-(*p*-tolyl)-1*H*-indole (**8b**) S41
4.22. 1-Methyl-2,3-diphenyl-1*H*-indole (**8c**) S42
4.23. 4-(1-Methyl-2-phenyl-1*H*-indol-3-yl)benzonitrile (**8d**) S43
5. References S44

1. General considerations

All reactions were carried out in heated Schlenk or multi-necked flasks under nitrogen atmosphere and using the septum and syringe technique, unless otherwise stated. The solvents used were dried by the MB-SPS 800 solvent drying system from M. Braun. Column chromatography was performed using silica gel M60 (230–400 mesh) from Macherey-Nagel GmbH & Co. KG, Düren. All column chromatographic separations were performed using flash technique at an overpressure of about 2 bar of compressed air. The crude products were previously adsorbed on Celite 545, particle size 0.02–0.10 mm from Merck KGAa. Silica yellow-coated aluminum foils with UV indicator (60 F254 Merck, Darmstadt) were used for thin layer chromatography. Detection was performed by using UV light (λ = 254 nm).

All commercially available chemicals were purchased from Acros, Merck KGAa, Alfa Aesar, VWR, Fluorochem, and Macherey-Nagel and used without further purification. ^{1}H,^{13}C, and 135-DEPT NMR spectra were recorded on a Bruker AV III 300 instrument. The resonance of the residual non-deuterated solvent used always served as the standard. Chloroform ($CDCl_3$, δ H 7.26, δ C 77.2) was used. Spin multiplicities were abbreviated as s (singlet), d (doublet), t (triplet), dd (doublet of a doublet), and m (multiplet). Methyl (CH_3), methylene (CH_2), methine (CH), and quaternary carbon nuclei were assigned using 135-DEPT spectra. Where necessary for identification, ^{1}H,^{1}H-COSY, HMQC, and HMBC spectra were also recorded in individual cases. EI mass spectra were recorded using a Finnigan MAT 8200 TSQ 7000 spectrometer. Indicated are all peaks with an intensity >10% of the base peak, the molecular ion peak, and any characteristic fragment peaks with an intensity <10%. IR spectra were recorded using an IRAffinity-1 instrument from Shimadzu. Measurements were made using the ATR technique. The intensities of the absorption bands are abbreviated as s (strong), m (medium), and w (weak). UV–vis spectra were recorded on a Perkin Elmer Lambda 19 spectrometer. All melting points were measured using a Büchi Melting Point B-540 apparatus. Elemental analyses were performed at the Institute of Pharmaceutical and Medicinal Chemistry, Heinrich Heine University, using a Perkin Elmer Series II Analyser 2400 instrument.

2. Starting materials

2.1. Di(adamantan-1-yl)(benzyl)phosphonium bromide[1]

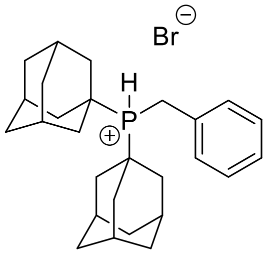

$C_{27}H_{38}BrP$

[473.47]

Di(adamantan-1-yl)phosphane (3.02 g, 10.0 mmol) and benzyl bromide (7.00 mL, 60.0 mmol) were suspended in 40 mL diethyl ether in a round-bottomed flask and the reaction suspension was stirred for 2 h at room temperature. Subsequently, the precipitated product collected by filtration under reduced pressure and washed three times with cold diethyl ether. The product was then dried under high vacuum to give a colorless solid (3.14 g, 67%).

^{1}H NMR (300 MHz, $CDCl_3$): δ = 1.65-2.32 (m, 30 H), 3.74 (dd, J = 13.3 Hz, 6.1 Hz, 2 H), 7.27-7.49 (m, 3 H), 7.63 (d, J = 8.0 Hz, 2 H), 9.06 (t, J = 6.1 Hz, 1 H). ^{13}C NMR (75 MHz, $CDCl_3$): δ = 19.8 (d, J_{CP} = 37.4 Hz, CH_2), 27.7 (d, J_{CP} = 9.3 Hz, CH), 35.6 (d, J_{CP} = 1.5 Hz, CH_2), 38.5 (d, J_{CP} = 2.8 Hz, CH_2), 38.6 (d, J_{CP} = 30.9 Hz, C_{quat}), 128.3 (d, J = 2.2 Hz, CH), 129.9 (d, J = 1.4 Hz, CH), 130.2 (d, J = 5.9 Hz, CH), 130.7 (d, J = 7.7 Hz, C_{quat}). EI MS (70 eV, *m/z* (%)): 392 ([M^+-HBr], 27), 136 (11), 135 ([$C_{10}H_{15}$], 100), 93 (10). IR $\tilde{\nu}$ [cm^{-1}]: 695 (s), 716 (m), 760 (m), 1454 (m), 1497 (m), 2855 (m), 2903 (s).

2.2. 2-Arylethynes 2

The 2-arylethynyes **2c** and **2f** were synthesized according to a literature procedure.[2]

3. Synthetic procedure and analytical data

3.1. General procedure GP for the synthesis of 1,2-disubstituted 3-iodoindoles 5

$PdCl_2(PPh_3)_2$ (17.4 mg, 25.0 μmol) and $(1\text{-}Ad)_2PBn\text{-}HBr$ (23.6 mg, 50 μmol) were placed in an oven-dried Schlenk tube with magnetic stirring bar under nitrogen. Then, the corresponding *o*-bromoaniline **1** (1.00 mmol), terminal alkyne **2** (1.20 mmol), DBU (457 mg, 3.00 mmol), and DMSO (1.50 mL) were added under nitrogen (for experimental details, see Table S1). The reaction mixture was heated at 100 °C (oil bath) for 2 h. After cooling to room temp potassium *tert*-butoxide (505 mg, 4.50 mmol) and DMSO (1.50 mL) were added to the reaction mixture and heated to 100 °C (oil bath) for 15 min. After cooling to room temp, *N*-iodosuccinimide (**3**, 338 mg, 1.50 mmol) and DMSO (1.00 mL) were added and stirred at room temp for 2 to 5 h (monitored by TLC). Then, the alkyl halide **4** (4.50 mmol) was added and the reaction mixture was stirred at room temp for 0.1 to 2 h (monitored by TLC). Deionized water (20 mL) was added to the reaction mixture and the aqueous phase was extracted with dichloromethane (3 × 20 mL). The combined organic phases were dried (anhydrous sodium sulfate), filtered, and the solvent was removed under vacuo. The residue was purified by chromatography on silica gel (*n*-hexane/ethyl acetate) to give the pure 1,2-disubstituted 3-iodoindoles **5**.

Table S1: Experimental details of the consecutive four-component synthesis of 1,2-disubstituted 3-iodoindoles **5**.

entry	*ortho*-bromo aniline **1**	alkyne **2**	alkyl halide **4**	1,2-disubstituted 3-iodoindoles **5**
1	172 mg (1.00 mmol) of *o*-bromaniline (**1a**)	122 mg (1.20 mmol) of phenylacetylene (**2a**)	639 mg (4.50 mmol) of methyliodide (**4a**)	167 mg (51%) of **5a**
2	186 mg (1.00 mmol) of 2-bromo-4-methylaniline (**1b**)	122 mg (1.20 mmol) of **2a**	639 mg (4.50 mmol) of **4a**	122 mg (35%) of **5b**
3	251 mg (1.00 mmol) of 2,4-dibromoaniline (**1c**)	122 mg (1.20 mmol) of **2a**	639 mg (4.50 mmol) of **4a**	100 mg (24%) of **5c**
3	298 mg (1.00 mmol) of 4-bromo-2-iodoaniline (**1d**)	122 mg (1.20 mmol) of **2a**	639 mg (4.50 mmol) of **4a**	165 mg (40%) of **5c**
4	237 mg (1.00 mmol) of 2-iodo-4-fluoroaniline (**1e**)	122 mg (1.20 mmol) of **2a**	639 mg (4.50 mmol) of **4a**	243 mg (69%) of **5d**
5	253 mg (1.00 mmol) of 2-	122 mg (1.20 mmol) of **2a**	639 mg (4.50 mmol) of **4a**	

	iodo-4-chloroaniline (**1f**)			229 mg (62%) of **5e**
6	172 mg (1.00 mmol) of **1a**	174 mg (1.20 mmol) of 4-dimethylaminophenylacetylene (**2b**)	639 mg (4.50 mmol) of **4a**	120 mg (32%) of **5f**
7	172 mg (1.00 mmol) of **1a**	158 mg (1.20 mmol) of 4-methoxyphenylacetylene (**2c**)	639 mg (4.50 mmol) of **4a**	207 mg (57%) of **5g**
8	172 mg (1.00 mmol) of **1a**	139 mg (1.20 mmol) of 4-tolylacetylene (**2d**)	639 mg (4.50 mmol) of **4a**	116 mg (34%) of **5h**
9	172 mg (1.00 mmol) of **1a**	4-trifluorphenylacetylene (**2e**)	639 mg (4.50 mmol) of **4a**	43 mg (11%) of **5i**
10	186 mg (1.00 mmol) of **1b**	158 mg (1.20 mmol) of **2c**	639 mg (4.50 mmol) of **4a**	224 mg (60%) of **5j**
11	186 mg (1.00 mmol) of **1b**	139 mg (1.20 mmol) of **2b**	639 mg (4.50 mmol) of **4a**	196 mg (54%) of **5k**
12	172 mg (1.00 mmol) of **1a**	79.3 mg 139 mg (1.20 mmol) of cyclopropylacetylene (**2f**)	639 mg (4.50 mmol) of **4a**	159 mg (53%) of **5l**
13	172 mg (1.00 mmol) of **1a**	100 mg (1.20 mmol) of 1-hexyne (**2g**)	639 mg (4.50 mmol) of **4a**	162 mg (52%) of **5m**
14	186 mg (1.00 mmol) of **1b**	100 mg (1.20 mmol) of **2d**	639 mg (4.50 mmol) of **4a**	109 mg (33%) of **5n**
15	172 mg (1.00 mmol) of **1a**	122 mg (1.20 mmol) of **2a**	684 mg (4.50 mmol) of benzylbromide (**4b**)	210 mg (52%) of **5o**
16	186 mg (1.00 mmol) of **1b**	122 mg (1.20 mmol) of **2a**	684 mg (4.50 mmol) of **4b**	211 mg (50%) of **5p**
17	298 mg (1.00 mmol) of **1d**	122 mg (1.20 mmol) of **2a**	684 mg (4.50 mmol) of **4b**	120 mg (25%) of **5q**
18	172 mg (1.00 mmol) of **1a**	122 mg (1.20 mmol) of **2a**	1125 mg (4.50 mmol) of 4-bromobenzyl-bromide (**4c**)	207 mg (43%) of **5r**

3.1.1 3-Iodo-1-methyl-2-phenyl-1*H*-indole (5a)[3]

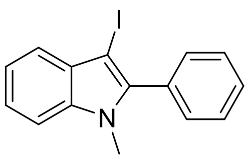

$C_{15}H_{12}IN$

[333.17]

According to the GP and after flash chromatography on silica gel (*n*-hexane/ethyl acetate 20:1 to ethyl acetate) compound **5a** (167 mg, 51%) was isolated as a yellow oil, R_f (*n*-hexane/ethyl acetate 10:1) = 0.47. ^{1}H NMR (300 MHz, CDCl$_3$): δ 3.60 (s, 3 H), 7.16-7.32 (m, 3 H), 7.37-7.55 (m, 6 H). ^{13}C NMR (75 MHz, CDCl$_3$): δ 32.0 (CH$_3$), 58.9 (C$_{quat}$), 109.9 (CH), 120.8 (CH), 121.5 (CH), 123.0 (CH), 128.5 (CH), 128.9 (CH), 130.5 (C$_{quat}$), 131.0 (CH), 131.6 (C$_{quat}$), 137.8 (C$_{quat}$), 141.8 (C$_{quat}$). EI MS (70 eV, *m/z* (%)): 334 (16), 333 ([M]$^+$, 100), 206 ([M – I]$^+$, 14), 205 (29),

204 (46), 179 (14), 178 (17), 102 (21), 56 (14). IR: $\tilde{\nu}$ [cm^{-1}]: 3053 (w), 3024 (w), 2990 (w), 2938 (w), 2880 (w), 2833 (w), 1605 (w), 1574 (w), 1462 (m), 1441 (m), 1427 (m), 1410 (w), 1377 (w), 1366 (w), 1356 (m), 1337 (m), 1308 (w), 1277 (w), 1231 (m), 1211 (m), 1194 (w), 1153 (m), 1128 (w), 1101 (m), 1074 (w), 1049 (w), 1022 (m), 1010 (m), 964 (w), 934 (m), 922 (m), 883 (w), 841 (w), 825 (m), 786 (m), 734 (s), 698 (s). HRMS *m/z* calcd. for $[C_{15}H_{12}IN+H]^+$: 334.0087; Found: 334.0085.

3.1.2 3-Iodo-1,5-dimethyl-2-phenyl-1*H*-indole (5b)[4,5]

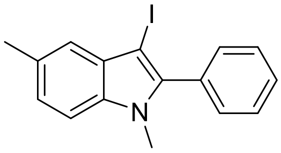

$C_{16}H_{14}IN$

[347.01]

According to the GP and after flash chromatography on silica gel (*n*-hexane/ethyl acetate 100:1 to 30:1) compound **5b** (122 mg, 35%) was isolated as a yellow solid, Mp 96.8 °C (lit.: oil;[4] 102-103 °C[5]), R_f (*n*-hexane/ethyl acetate 10:1) = 0.55. ^{1}H NMR (300 MHz, CDCl$_3$): δ 2.48 (s, 3 H), 3.61 (s, 3 H), 7.04-7.28 (m, 3 H), 7.35-7.51 (m, 5 H). ^{13}C NMR (75 MHz, CDCl$_3$): δ 21.5 (CH$_3$), 32.1 (CH$_3$), 58.3 (C$_{quat}$), 109.6 (CH), 121.1 (CH), 124.6 (CH), 128.4 (CH), 128.7 (CH), 130.2 (C$_{quat}$), 130.5 (C$_{quat}$), 130.9 (CH), 131.8 (C$_{quat}$), 136.2 (C$_{quat}$), 141.7 (C$_{quat}$). EI MS (70 eV, *m/z* (%)): 347 ([M]$^+$, 39), 222 (100), 221 (61), 205 (23), 179 (11). IR: $\tilde{\nu}$ [cm^{-1}]: 3906 (w), 3022 (w), 2936 (w), 2913 (w), 2857 (w), 2810 (w), 1603 (w), 1470 (m), 1429 (w), 1356 (w), 1333 (w), 1290 (w), 1279 (w), 1236 (w), 1209 (m), 1148 (w), 1105 (w), 1072 (w), 1022 (m), 961 (w), 920 (w), 860 (m), 797 (m), 772 (s), 742 (w), 712 (m), 696 (s), 669 (w). Anal. calcd. for $C_{16}H_{14}IN$ (347.0): C 59.35, H 4.06, N 4.03; Found: C 59.46, H 4.32, N 4.14.

3.1.3 5-Bromo-3-iodo-1-methyl-2-phenyl-1*H*-indole (5c)

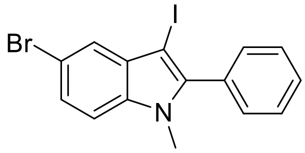

$C_{15}H_{11}BrIN$

[410.91]

According to the GP and after flash chromatography on silica gel (*n*-hexane/ethyl acetate 100:1 to 5:1) compound **5c** (80 mg, 40%) was isolated as a reddish solid, Mp 204.5 °C, R_f (*n*-hexane/ethyl acetate 10:1) = 0.55. ^{1}H NMR (600 MHz, CDCl$_3$): δ 3.66 (s, 3 H), 7.19 (d, J = 8.6 Hz, 1 H), 7.37-7.39 (m, 1 H), 7.44-7.55 (m, 5 H), 7.65 (d, *J* = 1.9 Hz, 1 H). ^{13}C NMR (150 MHz,

$CDCl_3$): δ 32.25 (CH_3), 57.87 (C_{quat}), 111.50 (CH), 114.06 (C_{quat}), 124.09 (CH), 125.84 (CH), 128.63 (CH), 129.19 (CH), 130.92 (CH), 131.22 (C_{quat}), 132.18 (C_{quat}), 136.64 (C_{quat}), 143.00 (C_{quat}). EI MS (70 eV, *m/z* (%)): 412 ([^{81}Br-M]$^+$, 49), 410 ([^{79}Br-M]$^+$, 50), 305 (13), 294 (78), 293 (70), 285 (19), 257 (92), 204 (90), 193 (62), 183 ([C_3H_6IN]$^+$, 100), 102 (40). IR: $\tilde{\nu}$ [cm^{-1}]: 611 (m), 664 (w), 679 (w), 691 (s), 702 (s), 716 (w), 752 (s), 787 (m), 806 (s), 839 (w), 870 (w), 916 (w), 970 (w), 1022 (w), 1038 (w), 1069 (w), 1103 (w), 1126 (w), 1148 (w), 1179 (w), 1209 (w), 1229 (w), 1260 (w), 1277 (w), 1298 (w), 1337 (w), 1362 (w), 1393 (w), 1431 (w), 1595 (w), 1954 (w), 2847 (w), 2922 (w), 2947 (w), 2968 (w), 2980 (w), 3022 (w), 3061 (w). HRMS *m/z* calcd. for [$C_{15}H_{11}BrIN+H$]$^+$: 411.9192; Found: 411.9191.

3.1.4 5-Fluoro-3-iodo-1-methyl-2-phenyl-1*H*-indole (5d)

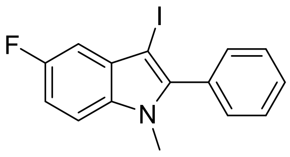

$C_{15}H_{11}FIN$

[350.99]

According to the GP and after flash chromatography on silica gel (*n*-hexane/ethyl acetate 100:1 to 5:1) compound **5d** (243 mg, 69%) was isolated as a beige solid, Mp 118.2 °C, R_f (*n*-hexane/ethyl acetate 10:1) = 0.50. ^{1}H NMR (600 MHz, $CDCl_3$): δ 3.68 (s, 3 H), 7.02-7.07 (m, 1 H), 7.17-7.20 (m, 1 H), 7.23-7.26 (m, 1 H), 7.46-7.54 (m, 5 H). ^{13}C NMR (150 MHz, $CDCl_3$): δ 32.4 (CH_3), 58.2 (C_{quat}), 106.7 (CH, J_{CF} = 27.2 Hz), 110.85 (d, J_{CF} = 9.0 Hz, CH), 111.40 (CH, J_{CF} = 27.2 Hz), 128.6 (CH), 129.3 (CH), 130.9 (CH), 130.11 (d, J_{CF} = 10.5 Hz, C_{quat}), 131.52 (d, J_{CF} = 3 Hz, C_{quat}), 134.5 (C_{quat}), 143.5 (C_{quat}), 158.8 (d, J_{CF} = 240 Hz, C_{quat}). EI MS (70 eV, *m/z* (%)): 351 ([M]$^+$, 2), 211 ($C_{14}H_{10}FN^+$, 100), 149 (19), 106 (12), 71 (10), 57 (22). IR: $\tilde{\nu}$ [cm^{-1}]: 604 (w), 619 (w), 662 (w), 689 (s), 733 (m), 756 (s), 789 (m), 860 (w), 907 (w), 934 (w), 957 (w), 997 (w), 1028 (w), 1051 (w), 1074 (w), 1107 (w), 1132 (m), 1165 (w), 1206 (m), 1238 (w), 1261 (w), 1275 (w), 1292 (w), 1315 (w), 1352 (w), 1406 (w), 1445 (w), 1456 (m), 1472 (w), 1541 (w), 1585 (w), 1622 (w), 1865 (w), 2853 (w), 2924 (w), 2961 (w), 3032 (w), 3957 (w), 3103 (w). HRMS *m/z* calcd. for [$C_{15}H_{11}FIN+H$]$^+$: 351.9993; Found: 351.9831.

3.1.5 5-Chloro-3-iodo-1-methyl-2-phenyl-1*H*-indole (5e)

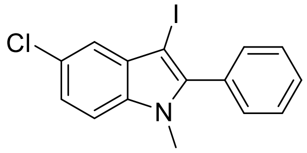

$C_{15}H_{11}ClIN$

[366.96]

According to the GP and after flash chromatography on silica gel (*n*-hexane/ethyl acetate 100:1 to 5:1) compound **5e** (129 mg, 62%) was isolated as a beige solid, Mp 104.1 °C, R_f (*n*-hexane/ethyl acetate 10:1) = 0.51. ^{1}H NMR (300 MHz, $CDCl_3$): δ 3.58 (s, 3 H), 7.11-7.18 (m, 2 H), 7.35-7.47 (m, 6 H). ^{13}C NMR (150 MHz, $CDCl_3$): δ 32.5 (CH_3), 58.2 (C_{quat}), 111.3 (CH), 121.2 (CH), 123.5 (CH), 126.8 (C_{quat}), 128.8 (CH), 129.4 (CH), 131.1 (CH), 131.5 (C_{quat}), 131.8 (C_{quat}), 136.6 (C_{quat}), 143.4 (C_{quat}). EI MS (70 eV, *m/z* (%)): 369 ([^{37}Cl-M]$^+$, 31), 367 ([^{35}Cl-M]$^+$, 100), 204 (65), 176 (15), 102 (36), 88 (11). IR: $\tilde{\nu}$ [cm^{-1}]: 652 (w), 685 (w), 700 (m), 735 (w), 758 (m), 791 (m), 820 (w), 951 (w), 1020 (w), 1040 (w), 1063 (w), 1107 (w), 1140 (w), 1177 (w), 1213 (w), 1231 (w), 1261 (w), 1333 (w), 1373 (w), 1427 (w), 1441 (w), 1464 (w), 1578 (w), 1607 (w), 2841 (w), 2884 (w), 2901 (w), 2967 (w), 2990 (w), 3052 (w). HRMS *m/z* calcd. for [$C_{15}H_{11}ClIN$+H]$^+$: 367.9698; Found: 367.9692.

3.1.6 4-(3-Iodo-1-methyl-1*H*-indol-2-yl)-*N*,*N*-dimethylaniline (5f)

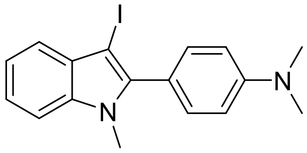

$C_{17}H_{17}IN_2$

[376.04]

According to the GP and after flash chromatography on silica gel (*n*-hexane/ethyl acetate 50:1 to 5:1) compound **5f** (120 mg, 32%) was isolated as a brown solid, Mp 207.2 °C, R_f (*n*-hexane/ethyl acetate 10:1) = 0.36. ^{1}H NMR (300 MHz, $CDCl_3$): δ 3.05 (s, 6 H), 3.69 (s, 3H), 6.81-6.87 (m, 2 H). EI MS (70 eV, *m/z* (%)): 376 ([M]$^+$, 100), 250 ([M – I]$^+$, 78), 234 (25), 204 (32), 187 (25), 124 (19).[1] IR: $\tilde{\nu}$ [cm^{-1}]: 610 (w), 638 (w), 658 (w), 689 (w), 714 (w), 745 (s), 818 (s), 843 (w), 899 (w), 932 (w), 943 (w), 1089 (w), 1061 (w), 1103 (w), 1225 (w), 1157 (w), 1167 (w), 1190 (m), 1225 (m), 1308 (w), 1335 (m), 1348 (m), 1427 (w), 1445 (w), 1460 (m), 1481 (m), 1493 (m), 1445 (w), 1611 (m), 1886 (w), 1440 (w), 2614 (w), 2805 (w), 2855 (w), 2889 (w), 3044 (w). HRMS *m/z* calcd. for [$C_{17}H_{17}IN_2$+H]$^+$: 377.0509; Found: 377.0508.

[1] The solution of compound **5f** tended to decompose after a short time so that it was not possible to record a meaningful ^{13}C NMR spectrum.

3.1.7 3-Iodo-2-(4-methoxyphenyl)-1-methyl-1*H*-indole (5g)

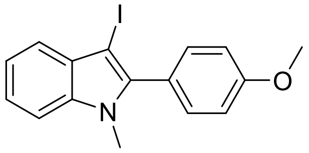

$C_{16}H_{14}INO$

[363.01]

According to the GP and after flash chromatography on silica gel (*n*-hexane/ethyl acetate 20:1 to 5:1) compound **5g** (207 mg, 57%) was isolated as a brown solid, Mp 126.2 °C, R_f (*n*-hexane/ethyl acetate 10:1) = 0.28. ^1H NMR (300 MHz, $CDCl_3$): δ 3.58 (s, 3 H), 3.80 (s, 3 H), 6.98-6.93 (m, 2 H), 7.19-7.11 (m, 1 H), 7.23-7.20 (m, 2 H), 7.33-7.28 (m, 2H), 7.43-7.39 (m, 1 H). ^{13}C NMR (75 MHz, $CDCl_3$): δ 32.0 (CH_3), 55.4 (CH_3), 58.8 (C_{quat}), 109.8 (CH), 113.9 (CH), 120.6 (CH), 121.3 (CH), 122.7 (CH), 123.8 (C_{quat}), 130.35 (C_{quat}), 132.2 (CH), 137.7 (C_{quat}), 141.7 (C_{quat}), 159.9 (C_{quat}). EI MS (70 eV, *m/z* (%)): 363 ($[M]^+$, 100), 348 ($[M - CH_3]^+$, 22), 193 (32). IR: $\tilde{\nu}$ [cm^{-1}]: 910 (w), 635 (w), 683 (w), 716 (w), 731 (w), 746 (s), 773 (w), 831 (m), 934 (w), 989 (w), 1009 (m), 1020 (s),1045 (m), 1074 (m), 1099 8m), 1130 (w), 1155 (w), 1175 (m), 1211 (w), 1246 (s), 1290 (w), 1334 (w), 1358 (w), 1369 (w), 1410 (w), 1420 (w), 1441 (w), 1452 (m), 1458 (m), 1477 (w), 1491 (m), 1576 (w), 1612 (w), 2837 (w), 2885 (m), 2901 (m), 2924 (m), 2968 (m), 3067 (w), 3649 (w). HRMS *m/z* calcd. for $[C_{16}H_{14}INO+H]^+$: 364.0154; Found: 364.0191.

3.1.8 3-Iodo-1-methyl-2-(*p*-tolyl)-1*H*-indole (5h)[6]

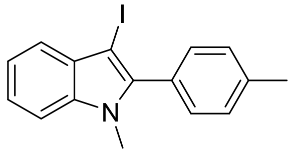

$C_{16}H_{14}IN$

[347.20]

According to the GP and after flash chromatography on silica gel (*n*-hexane/ethyl acetate 20:1 to 2:1) compound **5h** (116 mg, 34%) was isolated as a yellow solid, Mp 63.8 °C (lit.: 67-69 °C[6]), R_f (*n*-hexane/ethyl acetate 10:1) = 0.54. ^1H NMR (300 MHz, $CDCl_3$): δ 2.37 (s, 3 H), 3.56 (s, 3 H), 7.12-7.29 (m, 7 H), 7.43 (m, 1 H). ^{13}C NMR (75 MHz, $CDCl_3$): δ 21.6 (CH_3), 32.1 (CH_3), 58.8 (C_{quat}), 109.9 (CH), 120.7 (CH), 121.4 (CH), 122.9 (CH), 128.7 (C_{quat}), 129.3 (CH), 130.5 (C_{quat}), 130.8 (CH), 137.8 (C_{quat}), 138.8 (C_{quat}), 141.9 (C_{quat}). EI MS (70 eV, *m/z* (%)): 347 ($[M]^+$, 34), 221 ([M-I], 100), 220 (44), 205 (28), 204 (32), 178 (12), 109 (11), 102 (14), 86 (10), 57 (32), 56 (23). IR: $\tilde{\nu}$ [cm^{-1}]: 609 (m), 633 (w), 719 (w), 741 (s), 770 (w), 824 (m), 837 (w), 924 (w), 934 (m), 962 (w), 1011 (w), 1043 (w), 1101 (m), 1128 (w), 1155 (w), 1180 (w), 1217 (w),

1233 (w), 1306 (w), 1337 (w), 1358 (w), 1373 (w), 1410 (w), 1429 (w), 1460 (m), 2878 (w), 2916 (w), 2938 (w), 3022 (w), 3051 (w), 3069 (w). HRMS *m/z* calcd. for $[C_{16}H_{14}IN+H]^+$: 348.0244; Found: 348.0237.

3.1.9 3-Iodo-1-methyl-2-(4-(trifluormethyl)phenyl)-1*H*-indole (5i)

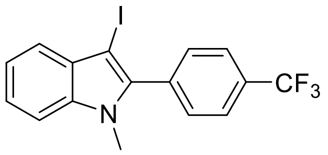

$C_{16}H_{11}F_3IN$

[400.98]

According to the GP and after flash chromatography on silica gel (*n*-hexane/ethyl acetate 20:1 to ethyl acetate) compound **5i** (43 mg, 11%) was isolated as a yellow oil, R_f (*n*- hexane/ethyl acetate 10:1) = 0.57. ^{1}H NMR (300 MHz, $CDCl_3$): δ 3.60 (s, 3 H), 7.15-7.19 (m, 1 H), 7.23-7.28 (m, 2 H), 7.42-7.45 (m, 1 H), 7.51-7.54 (m, 2 H), 7.69-7.71 (m, 2 H). ^{13}C NMR (150 MHz, $CDCl_3$): δ 32.2 (CH_3), 59.9 (C_{quat}), 110.1 (CH), 121.2 (CH), 121.9 (CH), 123.7 (CH), 124.71 (q, J_{CF} = 328 Hz, C_{quat}), 125.5 (q, J_{CF} = 3.8 Hz, CH), 130.6 (C_{quat}), 130.95 (q, J_{CF} = 39 Hz, CH), 131.1 (C_{quat}), 131.5 (CH), 135.6 (C_{quat}), 138.2 (C_{quat}), 140.2 (C_{quat}). EI MS (70 eV, *m/z* (%)): 401 ([M]$^+$, 100), 275 (13), 256 (10), 146 (10). IR: $\tilde{\nu}$ [cm^{-1}]: 682 (s), 734 (s), 786 (m), 825 (m), 841 (w), 883 (w), 922 (m), 945 (m), 964 (w), 1010 (m), 1022 (m), 1051 (w), 1074 (w), 1101 (m), 1142 (w), 1153 (m), 1194 (w), 1217 (m), 1231 (m), 1287 (w), 1308 (w), 1337 (m), 1366 (w), 1377 (w), 1410 (w), 1427 (m), 1441 (m), 1459 (m), 1574 (w), 1605 (w), 2833 (w), 2880 (w), 2938 (w), 2990 (w), 3024 (w). HRMS *m/z* calcd. for $[C_{16}H_{11}F_3IN]^+$: 400.9888; Found: 400.9981.

3.1.10 3-Iodo-2-(4-methoxyphenyl)-1,5-dimethyl-1*H*-indole (5j)

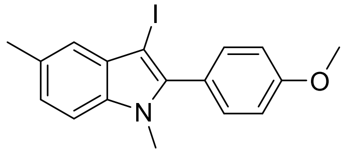

$C_{17}H_{16}INO$

[377.02]

According to the GP and after flash chromatography on silica gel (*n*-hexane/ethyl acetate 20:1 to 10:1) compound **5j** (224 mg, 60%) was isolated as a yellow solid, Mp 111.2 °C, R_f (*n*-hexane/ethyl acetate 10:1) = 0.55. ^{1}H NMR (300 MHz, $CDCl_3$): δ 2.50 (s, 3 H), 3.62 (s, 3 H), 3.87 (s, 3 H), 6.99-7.05 (m, 2 H), 7.07-7.27 (m, 3 H), 7.34-7.40 (m, 2 H). ^{13}C NMR (75 MHz, $CDCl_3$): δ 21.6 (CH_3), 32.1 (CH_3), 55.5 (CH_3), 58.3 (C_{quat}), 109.7 (CH), 114.0 (CH), 121.0 (CH), 124.0 (C_{quat}), 124.4 (CH), 130.2 (C_{quat}), 130.6 (C_{quat}), 132.3 (CH), 136.2 (C_{quat}), 141.7 (C_{quat}),

160.0 (C_{quat}). EI MS (70 eV, *m/z* (%)): 377 ([M]$^+$, 100), 252 (41), 237 (24), 207 (17), 193 (12), 127 (16). IR $\tilde{\nu}$ [cm^{-1}]: 667 (w), 696 (w), 714 (w), 743 (w), 772 (m), 797 (s), 839 (m), 864 (w), 922 (w), 961 (w), 1011 (w), 1152 (w), 1165 (w), 1177 (w), 1211 (w), 1238 (m), 1290 (w), 1329 (w), 1356 (w), 1368 (w), 1412 (w), 1431 (w), 1454 (w), 1477 (m), 1549 (w), 1612 (w), 2835 (w), 2905 (w), 2936 (w), 2953 (w), 3005 (w). Anal. calcd. for $C_{17}H_{16}INO$ (377.02): C 54.13, H 4.28, N 3.71; Found: C 54.32, H 4.21, N 3.54.

3.1.11 3-Iodo-1,5-dimethyl-2-(*p*-tolyl)-1*H*-indole (5k)

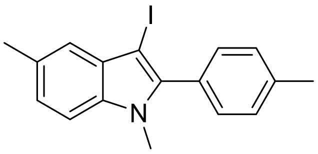

$C_{17}H_{16}IN$

[361.01]

According to the GP and after flash chromatography on silica gel (*n*-hexane/ethyl acetate 100:1 to 70:1) compound **5k** (196 mg, 54%) was isolated as a yellow solid, Mp 133.2 °C, R_f (*n*-hexane/ethyl acetate 10:1) =0.45. ^{1}H NMR (300 MHz, $CDCl_3$): δ 2.37 (s, 3 H), 2.43 (s, 3 H), 3.56 (s, 3 H), 7.02-7.29 (m, 7 H). ^{13}C NMR (75 MHz, $CDCl_3$): δ 21.5 (2 CH_3), 32.0 (CH_3), 58.1 (C_{quat}), 109.6 (CH), 121.0 (CH), 124.4 (CH), 128.8 (C_{quat}), 129.1 (CH), 130.1 (C_{quat}), 130.5 (CH), 130.8 (C_{quat}), 136.2 (C_{quat}), 138.7 (C_{quat}), 141.8 (C_{quat}). EI MS (70 eV, *m/z* (%)): 361 ([M]$^+$, 100), 235 (16), 219 (28), 109 (13). IR $\tilde{\nu}$ [cm^{-1}]: 644 (w), 675 (w), 733 (w), 771 (w), 791 (s), 824 (s), 864 (m), 924 (w), 959 (w), 1018 (w), 1038 (w), 1107 (w), 1146 (w), 1182 (w), 1217 (w), 1290 (w), 1325 (w), 1368 (w), 1379 (w), 1404 (w), 1427 (w), 1439 (w), 1477 (m), 1614 (w), 2857 (w), 2916 (w), 3014 (w), 3647 (w). Anal. calcd. for $C_{17}H_{16}IN$ (361.01): C 56.53, H 4.46, N 3.88; Found: C 56.67, H 4.60, N 3.73.

3.1.12 2-Cyclopropyl-3-iodo-1-methyl-1*H*-indole (5l)

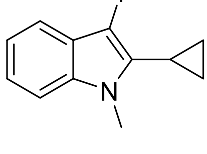

$C_{12}H_{12}IN$

[297.14]

According to the GP and after flash chromatography on silica gel (*n*-hexane/ethyl acetate 20:1 to 5:1) compound **5l** (153 mg, 53%) was isolated as a yellow oil, R_f (*n*-hexane/ethyl acetate 10:1) = 0.57. ^{1}H NMR (600 MHz, $CDCl_3$): δ 0.72-0.88 (m, 2 H), 0.91-1.09 (m, 2 H), 1.66 (tt, *J* = 8.3, 5.4 Hz, 1 H), 3.68 (s, 3 H), 6.96-7.19 (m, 3 H), 7.60 (d, *J* = 7.6 Hz, 1 H). ^{13}C NMR (150 MHz, $CDCl_3$): δ 7.2 (C_{quat}), 7.8 (CH_2), 30.5 (CH_3), 58.5 (C_{quat}), 109.1 (CH), 120.1 (CH), 120.8

(CH), 122.4 (CH), 130.0 (C_{quat}), 137.3 (C_{quat}), 140.0 (C_{quat}). EI MS (70 eV, *m/z* (%)): 297 ($[M]^+$, 100), 170 ($[M-I]^+$, 56), 168 (31), 154 (48),127 (29), 115 (20). IR $\tilde{\nu}$ [cm^{-1}]: 737 (s), 783 (w), 826 (m), 907 (w), 920 (w), 935 (w), 1011 (m), 1026 (w), 1103 (w), 1130 (w), 1159 (w), 1196 (w), 1231 (m), 1308 (w), 1319 (m), 1385 (m), 1433 (w), 1466 (w), 1530 (w), 2841 (w), 2884 (w), 2943 (w), 3003 (w), 3051 (w), 3080 (w). HRMS *m/z* calcd. for $[C_{12}H_{12}IN+H]^+$: 298.0087; Found: 298.0088.

3.1.13 2-Butyl-3-iodo-1-methyl-1*H*-indole (5m)

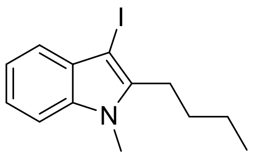

$C_{13}H_{16}IN$

[313.18]

According to the GP and after flash chromatography on silica gel (*n*-hexane/ethyl acetate 20:1 to 10:1) compound **5m** (162 mg, 52%) was isolated as a yellow oil, R_f (*n*-hexane/ethyl acetate 10:1) = 0.58. ^{1}H NMR (300 MHz, $CDCl_3$): δ 0.98 (t, J = 7.2 Hz, 3 H), 1.38-1.54 (m, 2 H), 1.54-1.68 (m, 2 H), 2.84-2.90 (m, 2 H), 3.76 (s, 3 H), 7.15-7.25 (m, 3 H), 7.37-7.42 (m, 1 H). ^{13}C NMR (75 MHz, $CDCl_3$): δ 13.9 (CH_3), 22.5 (CH_2), 26.9 (CH_2), 30.6 (CH_2), 31.5 (CH_3), 57.8 (C_{quat}), 109.2 (CH), 120.2 (CH), 120.5 (CH), 121.9 (CH), 130.0 (C_{quat}), 137.6 (C_{quat}), 141.9 (C_{quat}). EI MS (70 eV, *m/z* (%)): 313 ($[M]^+$, 100), 186 ($[M-I]^+$, 49), 174 (20). IR $\tilde{\nu}$ [cm^{-1}]: 644 (w), 679 (w), 702 (w), 735 (s), 839 (w), 880 (w), 935 (m), 961 (w), 1011 (m), 1080 (w), 1105 (m), 1128 (w), 1161 (m), 1202 (w), 1232 (m), 1312 (w), 1338 (w), 1358 (w), 1389 (w), 1414 (w), 1427 (w), 1442 (w), 1464 (s), 1530 (w), 1572 (w), 2857 (w), 2928 (w), 2953 (w), 3024 (w), 3053 (w). HRMS *m/z* calcd. for $[C_{13}H_{16}IN+H]^+$: 314.0400; Found: 314.0936.

3.1.14 2-Butyl-3-iodo-1,5-dimethyl-1*H*-indole (5n)

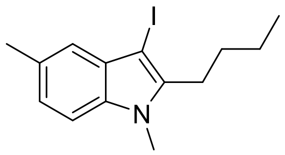

$C_{14}H_{18}IN$

[327.04]

According to the GP and after flash chromatography on silica gel (*n*-hexane/ethyl acetate 20:1 to 10:1) compound **5n** (109 mg, 33%) was isolated as a yellow oil, R_f (*n*-hexane/ethyl acetate 10:1) = 0.62. ^{1}H NMR (600 MHz, $CDCl_3$): δ 0.89 (t, J = 7.3 Hz, 3 H), 1.32-1.41 (m, 2 H), 1.45-1.53 (m, 2 H), 2.39 (s, 3 H), 2.72-2.78 (m, 2 H), 3.64 (s, 3 H), 6.93-7.18 (m, 3 H). ^{13}C NMR (150 MHz, $CDCl_3$): δ 14.1 (CH_3), 21.5 (CH_3), 22.6 (CH_2), 27.0 (CH_2), 30.8 (CH_3), 31.6 (CH_2),

57.3 (C_{quat}), 109.1 (CH), 120.3 (CH), 123.5 (CH), 129.8 (C_{quat}), 130.3 (C_{quat}), 136.0 (C_{quat}), 141.8 (C_{quat}). EI MS (70 eV, *m/z* (%)): 327 ($[M]^+$, 100), 201 ($[M - I]^+$, 33), 188 (17). IR $\tilde{\nu}$ [cm^{-1}]: 629 (w), 681 (w), 700 (w), 716 (w), 743 (w), 756 (w), 787 (s), 864 (w), 891 (w), 932 (w), 959 (w), 1995 (w), 1038 (w), 1070 (w), 1109 (w), 1144 (w), 1157 (w), 1171 (w), 1190 (w), 1236 (w), 1290 (w), 1329 (w), 1356 (w), 1387 (w), 1456 (w), 1485 (m), 1530 (w), 1574 (w), 2859 (w), 2926 (w), 2953 (w), 3011 (w). HRMS *m/z* calcd. for $[C_{14}H_{18}IN+H]^+$: 328.0562; Found: 328.0558.

3.1.15 1-Benzyl-3-iodo-2-phenyl-1*H*-indole (5o)[7]

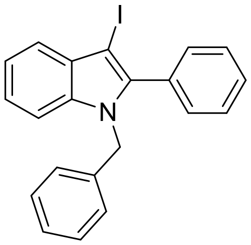

$C_{21}H_{16}IN$

[409.03]

According to the GP and after flash chromatography on silica gel (*n*-hexane/ethyl acetate 20:1 to 5:1) compound **5o** (210 mg, 52%) was isolated as a yellow solid, Mp 135.1 °C. R_f (*n*-hexane/ethyl acetate 10:1) = 0.49. ^1H NMR (300 MHz, $CDCl_3$): δ 5.29 (s, 2 H), 6.93-6.97 (m, 2 H), 7.16-7.31 (m, 5 H), 7.42-7.46 (m, 6 H), 7.57-7.65 (m, 1 H). ^{13}C NMR (75 MHz, $CDCl_3$): δ 48.7 (CH_2), 60.2 (C_{quat}), 110.8 (CH), 121.0 (CH), 121.6 (CH), 123.2 (CH), 126.0 (CH), 127.4 (CH), 128.5 (CH), 128.7 (CH), 129.0 (CH) 130.7 (C_{quat}), 130.9 (CH), 131.6 (C_{quat}), 137.3 (C_{quat}), 137.7 (C_{quat}), 142.0 (C_{quat}). EI MS (70 eV, *m/z* (%)): 409 ($[M]^+$, 30), 284 ($[M - I]^+$, 52), 205 (15), 191 (13), 165 (14), 91 ($[C_7H_7]$, 100). IR $\tilde{\nu}$ [cm^{-1}]: 608 (w), 648 (w), 667 (w), 698 (s), 731 (s), 746 (s), 762 (m), 787 (w), 829 (w), 905 (w), 918 (w), 935 (w), 966 (w), 1013 (w), 1028 (w), 1074 (w), 1115 (w), 1155 (w), 1171 (m), 1196 (w), 1229 (w), 1252 (w), 1277 (w), 1302 (w), 1341 (m), 1381 (w), 1414 (w), 1441 (m), 1493 (w), 1601 (w), 2860 (w), 2901 (w), 2914 (w), 3025 (w), 3055 (w). HRMS *m/z* calcd. for $[C_{21}H_{16}IN+H]^+$: 410.0406; Found: 410.0283.

3.1.16 1-Benzyl-3-iodo-5-methyl-2-phenyl-1*H*-indole (5p)

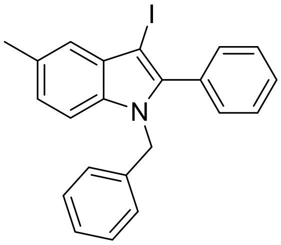

$C_{22}H_{18}IN$

[423.04]

According to the GP and after flash chromatography on silica gel (*n*-hexane/ethyl acetate 20:1 to 5:1) compound **5p** (211 mg, 50%) was isolated as a yellow solid, Mp 141.4 °C, R_f (*n*-hexane/ethyl acetate 10:1) = 0.60. ^{1}H NMR (300 MHz, $CDCl_3$): δ 2.51 (s, 3 H), 5.27 (s, 2 H), 6.91-6.96 (m, 2 H), 7.02-7.08 (m, 2 H), 7.20-7.25 (m, 3 H), 7.33 (s, 1 H), 7.37-7.46 (m, 5 H). ^{13}C NMR (75 MHz, $CDCl_3$): δ 21.6 (CH_3), 48.8 (CH_2), 59.7 (C_{quat}), 110.6 (CH), 121.3 (CH), 124.9 (CH), 126.1 (CH), 127.4 (CH), 128.5 (CH), 128.8 (CH), 129.0 (CH), 130.6 (C_{quat}), 130.9 (C_{quat}), 131.8 (C_{quat}), 135.8 (C_{quat}), 137.9 (C_{quat}), 142.0 (C_{quat}). EI MS (70 eV, *m/z* (%)): 423 ($[M]^+$, 97), 297 ($[M - I]^+$, 73), 219 (22), 206 (30), 190 (18), 91 ($[C_7H_7]^+$, 100). IR $\tilde{\nu}$ [cm^{-1}]: 646 (w), 681 (w), 696 (s), 725 (s), 737 (m), 772 (m), 789 (s), 808 (w), 864 (w), 924 (w), 961 (w), 1030 (w), 1069 (w), 1157 (w), 1177 (w), 1190 (w), 1231 (w), 1256 (w), 1290 (w), 1329 (w), 1350 (w), 1385 (w), 1433 (w), 1441 (w), 1452 (m), 1470 (w), 1603 (w), 2853 (w), 2918 (w), 3015 (w), 3026 (w), 3055 (w). HRMS *m/z* calcd. for $[C_{22}H_{18}IN+H]^+$: 424.0562; Found: 424.0557.

3.1.17 1-Benzyl-5-bromo-3-iodo-2-phenyl-1*H*-indole (5q)

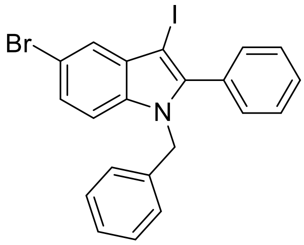

$C_{21}H_{15}BrIN$

[486.94]

According to the GP and after flash chromatography on silica gel (*n*-hexane/ethyl acetate 20:1 to 5:1) compound **5q** (120 mg, 25%) was isolated as a yellow solid, Mp 140.9 °C, R_f (*n*-hexane/ethyl acetate 10:1) = 0.49. ^{1}H NMR (300 MHz, $CDCl_3$): δ 5.32 (d, J = 8.5 Hz, 2 H), 6.94-6.97 (m, 1 H), 7.04-7.21 (m, 1 H), 7.26-7.30 (m, 3 H), 7.37-7.48 (m, 6 H), 7.58-7.62 (m, 1 H), 7.70-7.82 (m, 1 H). ^{13}C NMR (75 MHz, $CDCl_3$): δ 48.9 (CH_2), 60.4 (C_{quat}), 111.0 (CH), 112.4 (CH), 115.8 (CH), 123.9 (C_{quat}), 125.5 (C_{quat}), 126.1 (CH), 128.4 (CH), 128.7 (CH), 128.9 (CH), 130.9 (CH), 131.7 (CH), 137.2 (C_{quat}), 137.3 (C_{quat}), 137.4 (C_{quat}), 143.1 (C_{quat}). EI MS (70 eV,

m/z (%)): 489 ([^{81}Br-M]$^+$, 38), 487 ([^{79}Br-M]$^+$, 37), 281 ([M – I – Br]$^+$, 36), 190 (24), 163 (15), 91 ([C_7H_7]$^+$, 100), 65 (13). IR $\tilde{\nu}$ [cm^{-1}]: 624 (w), 665 (w),687 (s), 700 (s), 727 (m),754 (s), 785 (w), 845 (w), 866 (w), 876 (m), 899 (w), 945 (w), 964 (w), 1028 (w), 1051 (w), 1072 (m), 1111 (w), 1128 (w), 1157 (w), 1169 (m), 1198 (w), 1233 (w), 1254 (w), 1277 (w), 1306 (w), 1329 (w), 1350 (w), 1371 (w), 1383 (w), 1412 (w), 1441 (m), 1452 (w), 1483 (w), 1493 (w), 1593 (w), 2868 (w), 2901 (w), 2918 (w), 2968 (w), 3024 (w). HRMS *m/z* calcd. for [$C_{21}H_{15}{}^{79}BrIN$+H]$^+$: 487.9511; Found: 487.9421.

3.1.18 1-(4-Bromobenzyl)-3-iodo-2-phenyl-1*H*-indole (5r)

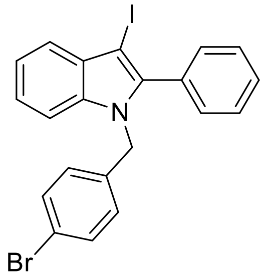

$C_{21}H_{15}BrIN$

[486.94]

According to the GP and after flash chromatography on silica gel (*n*-hexane/ethyl acetate 20:1 to 5:1) compound **5r** (207 mg, 43%) was isolated as a yellow solid, Mp 133.6 °C, R_f (*n*-hexane/ethyl acetate 10:1) = 0.50. ^{1}H NMR (300 MHz, CDCl$_3$): δ 5.12 (s, 2 H), 6.71-6.67 (m, 2 H), 7.01-7.10 (m, 1 H), 7.14-7.18 (m, 2 H), 7.22-7.27 (m, 2 H), 7.27-7.32 (m, 2 H), 7.32-7.37 (m, 3 H), 7.46-7.53 (m, 1 H). ^{13}C NMR (75 MHz, CDCl$_3$): δ 48.1 (CH$_2$), 60.6 (C_{quat}), 110.6 (CH), 121.2 (CH), 121.3 (C_{quat}), 121.7 (CH), 123.4 (CH), 127.8 (CH), 128.6 (CH), 129.1 (CH), 130.7 (C_{quat}), 130.8 (CH), 131.4 (C_{quat}), 131.8 (CH), 136.4 (C_{quat}), 137.2 (C_{quat}), 141.8 (C_{quat}). EI MS (70 eV, *m/z* (%)): 489 ([^{81}Br-M]$^+$, 60), 487 ([^{79}Br-M]$^+$, 58), 363 (26), 281 (23), 202 (34), 170 ([$C_7H_6{}^{79}Br$], 100), 90 ([C_7H_6]$^+$, 36), 84 (44) IR $\tilde{\nu}$ [cm^{-1}]: 604 (w), 627 (w), 658 (m), 702 (s), 741 (s), 781 (w), 797 (m), 812 (m), 837 (w), 908 (w), 943 (w), 961 (w), 972 (w), 986 (w), 1011 (m), 1030 (w), 1070 (m), 1111 (w), 1157 (w), 1167 (m), 1184 (w), 1229 (w), 1258 (w), 1269 (w),1296 (w), 1312 (w), 1341 (w), 1362 (w), 1404 (w), 1435 (w), 1456 (w), 1485 (w), 1601 (w), 2853 (w), 2901 (w), 2911 (w), 2965 (w), 3026 (w), 3055 (w). HRMS *m/z* calcd. for [$C_{21}H_{15}{}^{81}BrIN$]$^+$: 486.9433; Found: 484.9428.

3.3. Synthesis of 3-iodo-1-methyl-2-phenyl-5-(phenylethynyl)-1*H*-indole (6)

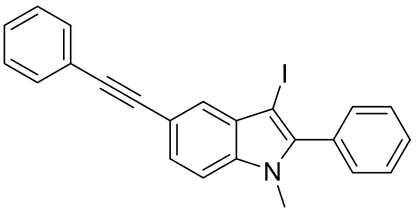

$C_{23}H_{16}IN$

[433.03]

$PdCl_2(PPh_3)_2$ (17.4 mg, 25.0 µmol) and $(1\text{-Ad})_2PBn\text{-}HBr$ (23.6 mg, 50 µmol) were placed in an oven-dried Schlenk tube with magnetic stirring bar under nitrogen. Then, 2,4-dibromoaniline (**1c**, 254 mg, 1.00 mmol), phenylacetylene (**2a**, 245 mg, 2.40 mmol), DBU (457 mg, 3.00 mmol), and 1.50 mL DMSO were added and flushed with nitrogen. The reaction mixture was heated at 100 °C until complete conversion of the starting material (via TLC control). Potassium *tert*-butoxide (505 mg, 4.50 mmol) and 1.50 mL DMSO were then added to the reaction mixture and stirred for an additional 15 min. After cooling the reaction mixture to room temperature, NIS (338 mg, 1.50 mmol) and 1.00 mL DMSO were added. After complete conversion (via TLC control), methyl iodide (639 mg, 4.50 mmol) was added and the mixture also stirred at room temperature for 5 min. Then, water was added and the aqueous phase was extracted with dichloromethane. The combined organic phases were dried with anhydrous sodium sulfate, filtered and the solvent was then removed under reduced pressure. The residue was purified by chromatography on silica gel (*n*-hexane/ethyl acetate 20:1 to 5:1) to give compound **6** (184 mg, 42%) as a colorless solid, Mp 204.5 °C, R_f (*n*-hexane/ethyl acetate 10:1) = 0.35. ^{1}H NMR (300 MHz, $CDCl_3$): δ 3.69 (s, 3 H), 7.27-7.30 (m, 1 H), 7.31-7.39 (m, 3 H), 7.46-7.56 (m, 6 H), 7.57-7.60 (m, 2 H), 7.72-7.75 (m, 1 H). ^{13}C NMR (75 MHz, $CDCl_3$): δ 32.3 (CH_3), 59.2 (C_{quat}), 87.9 (C_{quat}), 90.8 (C_{quat}), 110.1 (CH), 115.5 (C_{quat}), 123.9 (C_{quat}), 125.4 (CH), 126.6 (CH), 128.0 (CH), 128.5 (CH), 128.6 (CH), 129.1 (CH), 130.5 (C_{quat}), 131.0 (CH), 131.4 (C_{quat}), 131.7 (CH), 137.6 (C_{quat}), 142.9 (C_{quat}). EI MS (70 eV, *m/z* (%)): 433 ([M], 100), 304 ([M-I], 38), 227 (11), 153 (29). IR: $\tilde{\nu}$ [cm^{-1}]: 611 (m), 621 (w), 664 (w), 679 (m), 691 (s), 702 (s), 754 (s), 787 (m), 806 (s), 870 (w), 916 (w), 970 (w), 1022 (w), 1069 (w), 1103 (w), 1148 (w), 1179 (w), 1209 (w), 1229 (w), 1277 (w), 1298 (w), 1337 (w), 1364 (w), 1431 (w), 1441 (w), 1473 (w), 1493 (w), 1595 (w), 1873 (w), 1954 (w), 2029 (w), 2810 (w), 2847 (w), 2893 (w). Anal. calcd. for $C_{23}H_{16}IN$ (433.03): C 63.76, H 3.72, N 3.23; Found: C 63.81, H 3.74, N 2.96.

3.4. General procedure GP for the synthesis of 1,2,3-trisubstituted indoles 8

3-Iodoindole **5a** (167 mg, 0.50 mmol), arylboronic acid **7** (1.50 mmol), $Pd(PPh_3)_4$ (28.9 mg, 25.0 µmol), and cesium carbonate (652 mg, 2.00 mmol) were placed in an oven-dried Schlenk tube with magnetic stirring bar under nitrogen (for experimental details, see Table S2). Under

nitrogen, DMSO (5.00 mL) and deionized water (0.80 mL) were added and the reaction mixture was heated to 85 °C (oil bath) for 2 h. Deionized water (20 mL) was added to the reaction mixture and the aqueous phase was extracted with dichloromethane (3 × 20 mL). The combined organic phases were dried (anhydrous sodium sulfate), filtered, and the solvent was then removed under vacuo. The residue was purified by chromatography on silica gel (*n*-hexane/ethyl acetate) to give the pure 1,2,3-trisubstituted 3-iodoindoles **8**.

Table S2: Suzuki synthesis of 1,2,3-trisubstituted indoles **8**.

entry	boronic acid **7**	1,2,3-trisubstituted indole **8**
1	228 mg (1.50 mmol) of 4-methoxyphenyl boronic acid (**7a**)	133 mg (85%) of **8a**
2	204 mg (1.50 mmol) of *p*-tolyl boronic acid (**7b**)	105 mg (71%) of **8b**
3	183 mg (1.50 mmol) of phenyl boronic acid (**7c**)	111 mg (79%) of **8c**
4	220 mg (1.50 mmol) of *p*-cyanophenyl boronic acid (**7d**)	140 mg (91%) of **8d**

3.4.1. 3-(4-Methoxyphenyl)-1-methyl-2-phenyl-1*H*-indole (8a)[8]

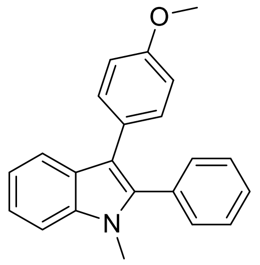

$C_{22}H_{19}NO$

[313.14]

According to the GP (AS2) and after flash chromatography on silica gel (*n*-hexane/ethyl acetate 20:1 to 5:1) compound **8a** (106 mg, 85%) was isolated as a colorless solid, Mp 103.9 (lit.: 128-129 °C[8]), R_f (*n*-hexane/ethyl acetate 10:1) = 0.51. ^{1}H NMR (300 MHz, $CDCl_3$): δ 3.66 (s, 3 H), 3.78 (s, 3 H), 6.79-6.85 (m, 2 H), 7.15-7.23 (m, 3 H), 7.28-7.40 (m, 7 H), 7.72-7.78 (m, 1 H). ^{13}C NMR (75 MHz, $CDCl_3$): δ 31.1 (CH_3), 55.3 (CH_3), 109.6 (CH), 113.9 (CH), 114.3 (CH), 114.9 (C_{quat}), 120.2 (CH), 122.2 (CH), 127.3 (C_{quat}), 127.7 (C_{quat}), 127.9 (CH), 128.0 (CH), 128.5 (CH), 131.0 (CH), 131.3 (C_{quat}), 132.3 (C_{quat}), 137.5 (C_{quat}), 157.7 (C_{quat}). EI MS (70 eV, *m/z* (%)): 313 ([M], 15), 214 (100), 171 (33), 156 (11), 128 (26). IR $\tilde{\nu}$ [cm^{-1}]: 625 (w), 650 (w), 677 (w), 700 (s), 723 (m), 741 (s), 781 (m), 806 (w), 829 (w), 847 (w), 893 (w), 918 (w), 939 (w), 997 (w), 1022 (w), 1040 (m), 1092 (w), 1111 (w), 1136 (w), 1177 (m), 1242 (s), 1277 (w), 1329 (w), 1369 (w), 1395 (w), 1418 (w), 1435 (w), 1462 (w), 1479 (w), 1501 (w), 1512 (m), 1549 (w),

1607 (w), 2828 (w), 2889 (w), 2902 (w), 2928 (w), 2945 (w), 3005 (w), 3649 (w). Anal. calcd. for $C_{22}H_{19}NO$ (313.14): C 84.31, H 6.11, N 4.47; Found: C 84.34, H 5.95, N 4.18.

3.4.2. 1-Methyl-2-phenyl-3-(*p*-tolyl)-1*H*-indole (8b)[9]

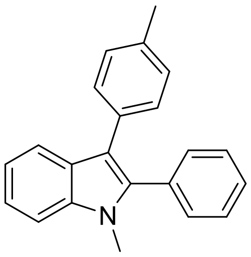

$C_{22}H_{19}N$

[297.15]

According to the GP (AS2) and after flash chromatography on silica gel (*n*-hexane/ethyl acetate 20:1 to 5:1) compound **8b** (105 mg, 71%) was isolated as a colorless solid, Mp 150.8 °C (lit.: 157 °C[9]). R_f (*n*-hexane/ethyl acetate 10:1) = 0.58. ^{1}H NMR (300 MHz, $CDCl_3$): δ 2.24 (s, 3 H), 3.58 (s, 3 H), 6.98-7.02 (m, 2 H), 7.08-7.14 (m, 3 H), 7.40-7.17 (m, 7 H), 7.68-7.73 (m, 1 H). ^{13}C NMR (75 MHz, $CDCl_3$): δ 21.3 (CH_3), 31.1 (CH_3), 109.7 (CH), 115.1 (C_{quat}), 119.8 (CH), 120.2 (CH), 122.2 (CH), 127.2 (C_{quat}), 128.1 (CH), 128.5 (CH), 129.1 (CH), 129.8 (CH), 131.3 (CH), 132.2 (C_{quat}), 132.2 (C_{quat}), 135.1 (C_{quat}), 137.4 (C_{quat}), 137.6 (C_{quat}). EI MS (70 eV, *m/z* (%)): 297 ([M], 1), 208 ([$C_{15}H_{12}N$], 100), 180 (12), 165 (12). IR $\tilde{\nu}$ [cm^{-1}]: 698 (s), 721 (m), 741 (s), 783 (w), 810 (m), 918 (w), 939 (m), 1005 (w), 1020 (m), 1037 (w), 1072 (w), 1088 (m), 1117 (w), 1138 (w), 1227 (w), 1261 (w), 1306 (w), 1329 (m), 1368 (m), 1391 (w), 1414 (w), 1431 (w), 1460 (m), 1499 (w), 1512 (w), 1545 (w), 1607 (w), 2855 (w), 2914 (w), 2961 (w), 3013 (w), 3026 (w), 3053 (w). Anal. calcd. for $C_{22}H_{19}N$ (297.15): C 88.85, H 6.44, N 4.71; Found: C 88.65, H 6.29, N 4.52.

3.4.3. 1-Methyl-2,3-diphenyl-1*H*-indole (8c)[10]Fehler! Textmarke nicht definiert.

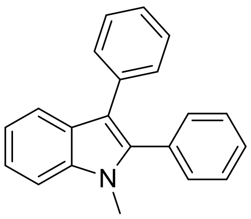

$C_{21}H_{17}N$

[283.13]

According to the GP (AS2) and after flash chromatography on silica gel (*n*-hexane/ethyl acetate 20:1 to 5:1) compound **8c** (111 mg, 79%) was isolated as a colorless solid, Mp 127.9 °C (lit.: 137-139 °C[10]), R_f (*n*-hexane/ethyl acetate 10:1) = 0.75. ^{1}H NMR (300 MHz, $CDCl_3$): δ 3.59 (s,

3 H), 7.05-7.35 (m, 13 H), 7.69-7.74 (m, 1 H). ^{13}C NMR (75 MHz, $CDCl_3$): δ 31.1 (CH_3), 109.7 (CH), 115.2 (C_{quat}), 119.7 (CH), 120.3 (CH), 122.3 (CH), 125.6 (CH), 127.1 (C_{quat}), 128.2 (CH), 128.3 (CH), 128.5 (CH), 130.0 (CH), 131.3 (CH), 132.0 (C_{quat}), 135.4 (C_{quat}), 137.5 (C_{quat}), 137.9 (C_{quat}). EI MS (70 eV, *m/z* (%)): 283 ([M], 13), 207 ([$C_{15}H_{12}N$], 100), 179 (12), 165 (14), 102 (10). IR $\tilde{\nu}$ [cm^{-1}]: 617 (w), 664 (w), 698 (s), 737 (s), 772 (w), 802 (w), 914 (w), 939 (w), 1018 (w), 1078 (w), 1090 (w), 1138 (w), 1152 (w), 1169 (w), 1179 (w), 1217 (w), 1229 (w), 1258 (w), 1310 (w), 1329 (w), 1369 (w), 1398 (w), 1429 (w), 1462 (w), 1470 (w), 1497 (w), 1445 (w), 1597 (w), 2868 (w), 2905 (w), 3028 (w), 3049 (w). Anal. calcd. for $C_{21}H_{17}N$ (283.13): C 89.01, H 6.05, N 4.94; Found: C 88.93, H 5.76, N 4.82.

3.4.4. 4-(1-Methyl-2-phenyl-1*H*-indol-3-yl)benzonitrile (8d)[11]

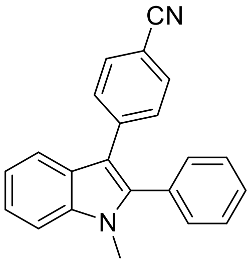

$C_{22}H_{16}N_2$

[308.13]

According to the GP (AS2) and after flash chromatography on silica gel (*n*-hexane/ethyl acetate 20:1 to 5:1) compound **8d** (140 mg, 91%) was isolated as a colorless solid, Mp 194.2 °C (lit.: 190-191 °C[11]), R_f (*n*-hexane/ethyl acetate 10:1) = 0.42. ^{1}H NMR (300 MHz, $CDCl_3$): δ 3.60 (s, 3 H), 7.12-7.47 (m, 12 H), 7.66-7.72 (m, 1 H). ^{13}C NMR (75 MHz, $CDCl_3$): δ 31.1 (CH_3), 108.7 (C_{quat}), 110.1 (CH), 113.6 (C_{quat}), 119.2 (CH), 119.5 (C_{quat}), 121.1 (CH), 122.9 (CH), 126.4 (C_{quat}), 128.8 (CH), 128.9 (CH), 130.1 (CH), 131.1 (CH), 131.3 (C_{quat}), 132.2 (CH), 137.6 (C_{quat}), 139.1 (C_{quat}), 140.8 (C_{quat}). EI MS (70 eV, *m/z* (%)): 308 ([M], 100), 292 ([M-CH_3], 19), 146 (15). IR $\tilde{\nu}$ [cm^{-1}]: 633 (m), 706 (s), 746 (s), 806 (w), 824 (w), 860 (m), 897 (w), 928 (w), 1024 (w), 1090 (w), 1134 (w), 1155 (w), 1177 (w), 1233 (w), 1258 (w), 1277 (w), 1329 (w), 1362 (w), 1398 (w), 1468 (m), 1603 (m), 1904 (w), 2828 (w), 2847 (w), 2886 (w), 2967 (w), 2990 (w), 3022 (w), 3071 (w). Anal. calcd. for $C_{22}H_{16}N_2$ (308.13): C 85.69, H 5.23, N 9.08; Found: C 85.82, H 5.31, N 9.00.

4. ^{1}H and ^{13}C NMR spectra of compounds 5, 6, and 8

4.1. 3-Iodo-1-methyl-2-phenyl-1*H*-indole (5a)

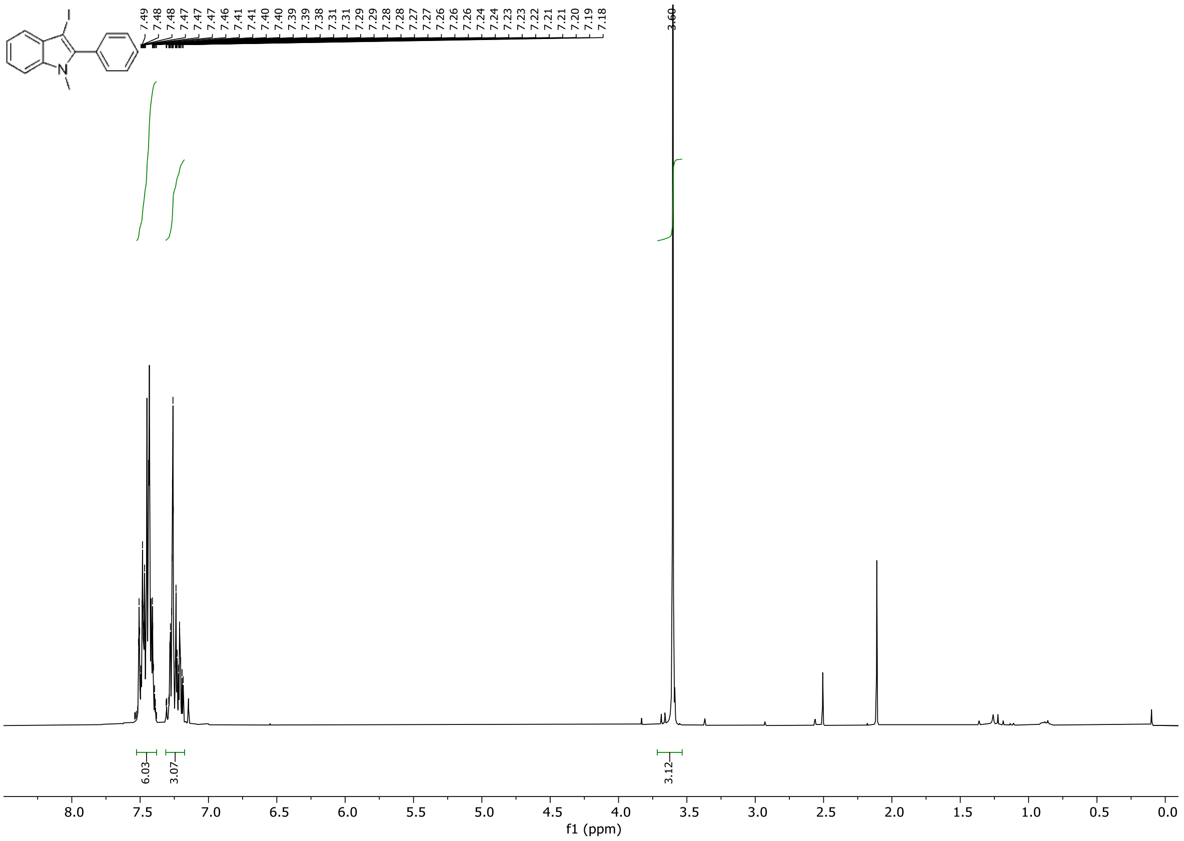

Figure S1. ^{1}H NMR spectrum (300 MHz, recorded in $CDCl_3$, T = 298 K).

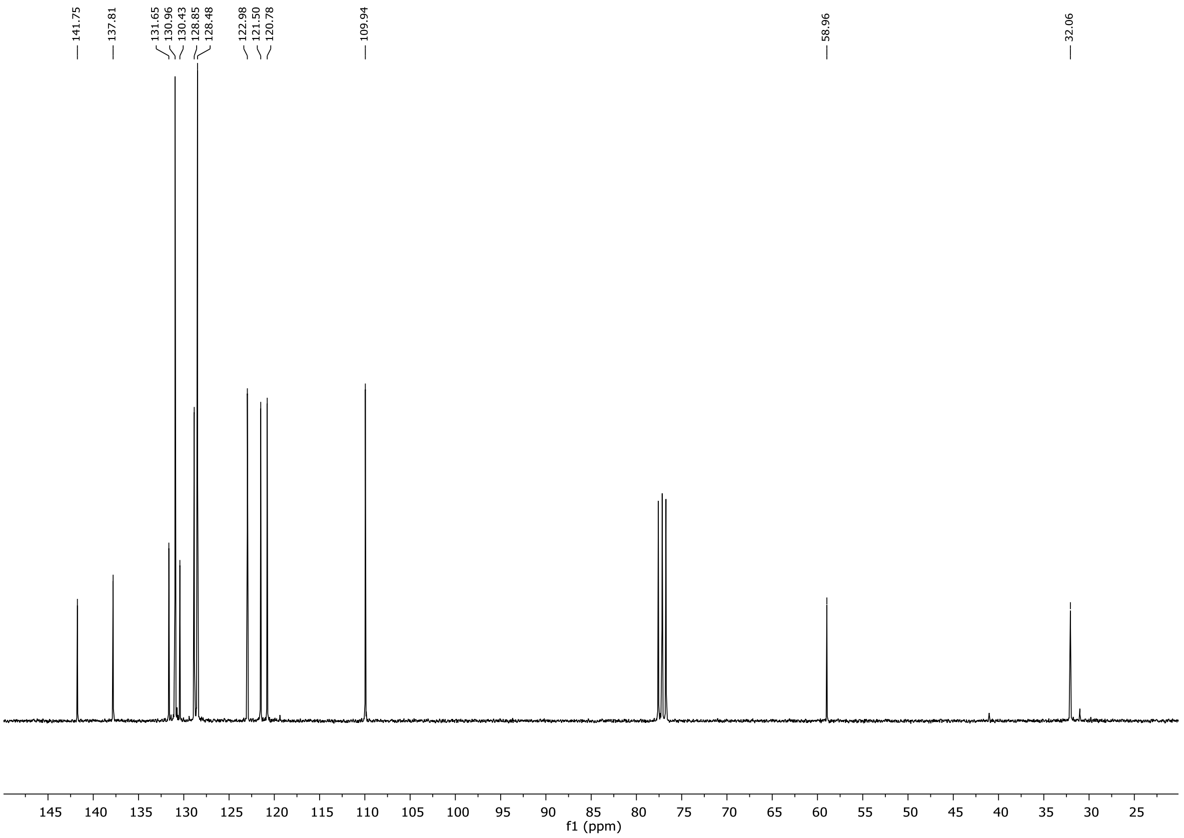

Figure S2. ^{13}C NMR spectrum (75 MHz, recorded in $CDCl_3$, T = 298 K).

4.2. 3-Iodo-1,5-dimethyl-2-phenyl-1*H*-indole (5b)

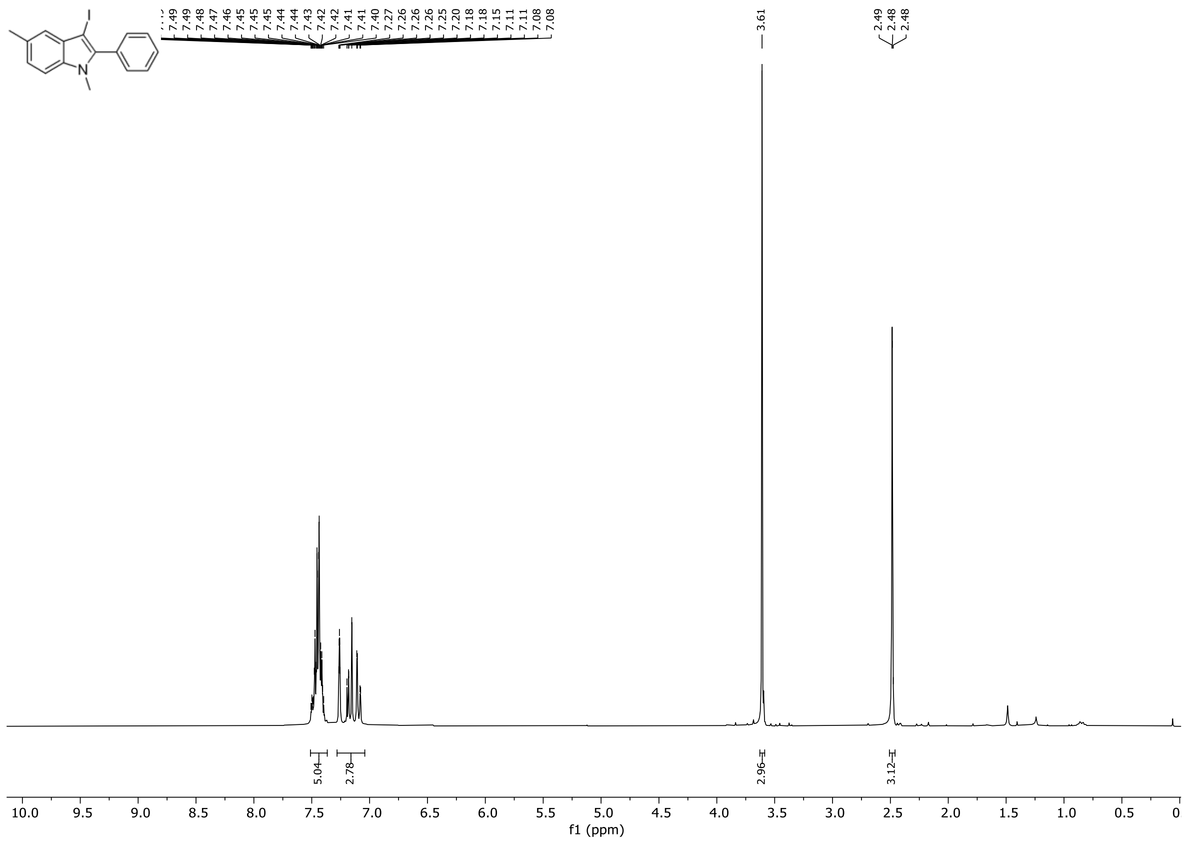

Figure S3. ^{1}H NMR spectrum (300 MHz, recorded in $CDCl_3$, T = 298 K).

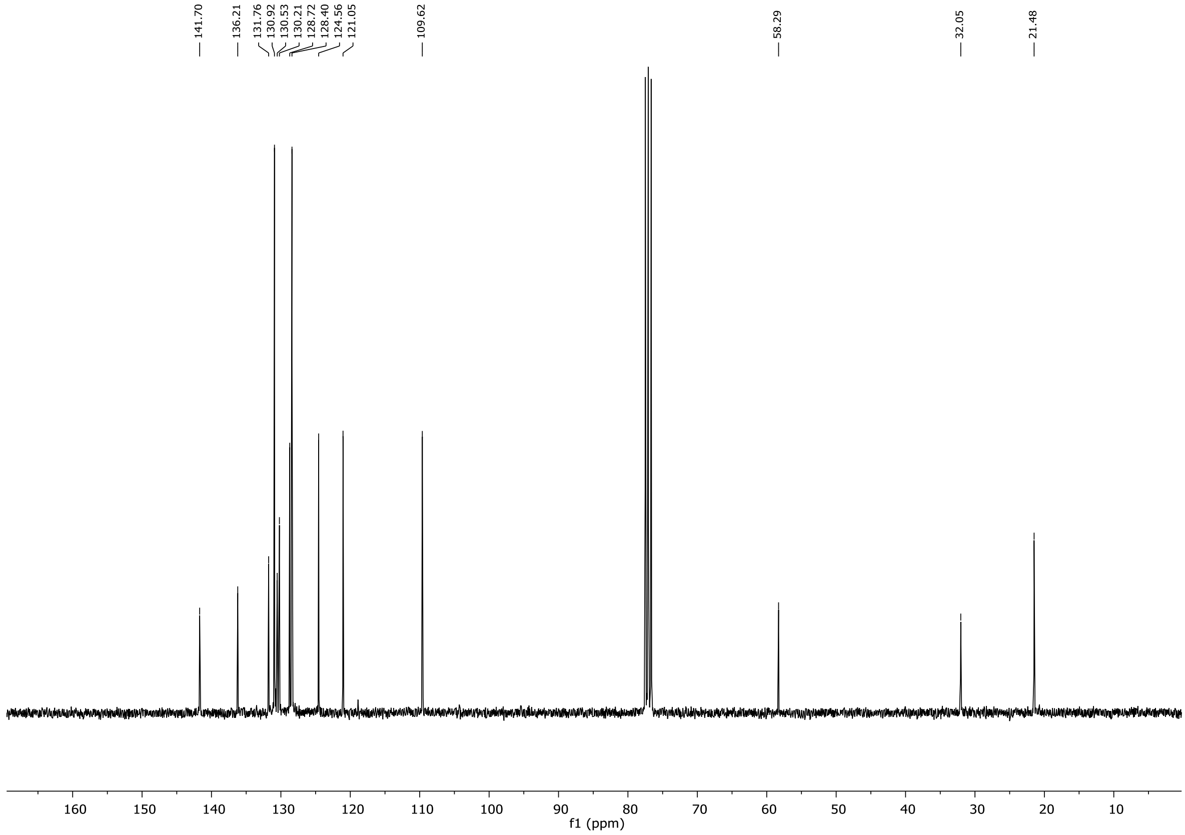

Figure S4. ^{13}C NMR spectrum (75 MHz, recorded in $CDCl_3$, T = 298 K).

4.3. 5-Bromo-3-iodo-1-methyl-2-phenyl-1*H*-indole (5c)

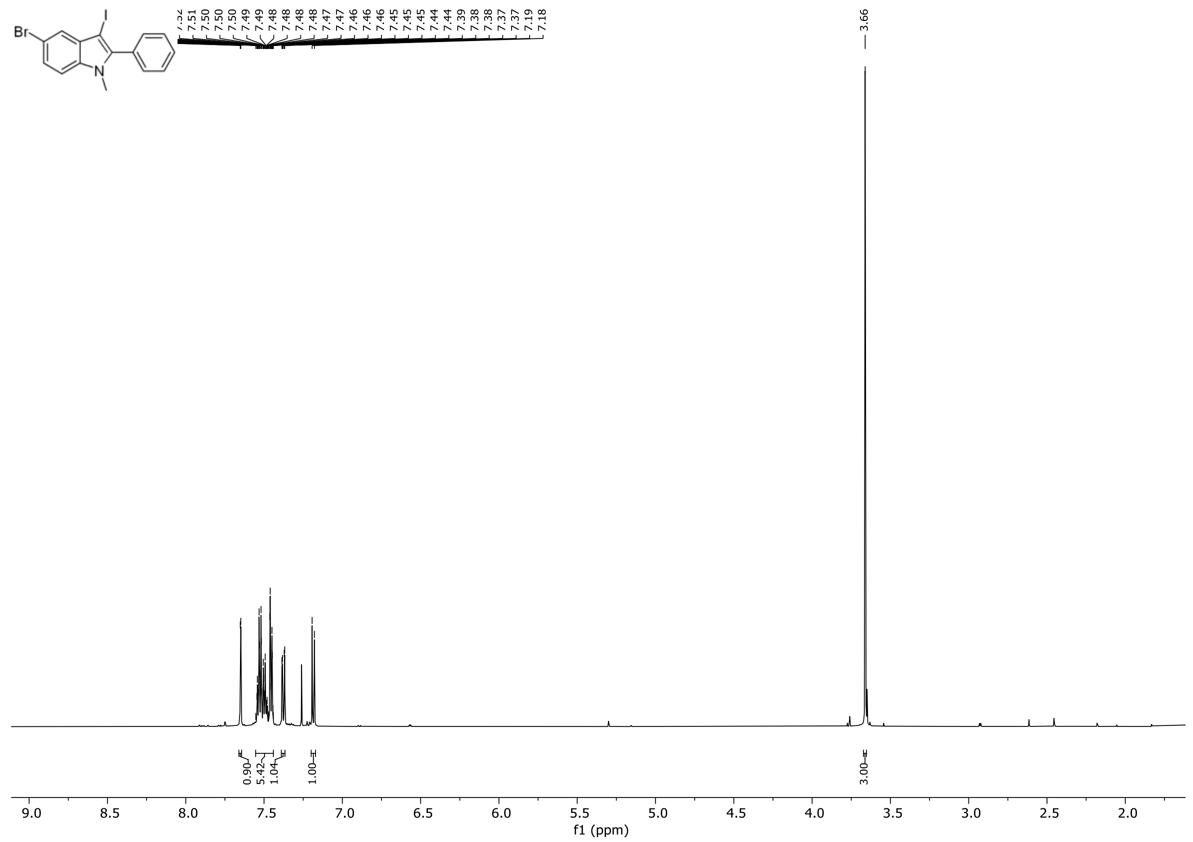

Figure S5. ^{1}H NMR spectrum (300 MHz, recorded in $CDCl_3$, T = 298 K).

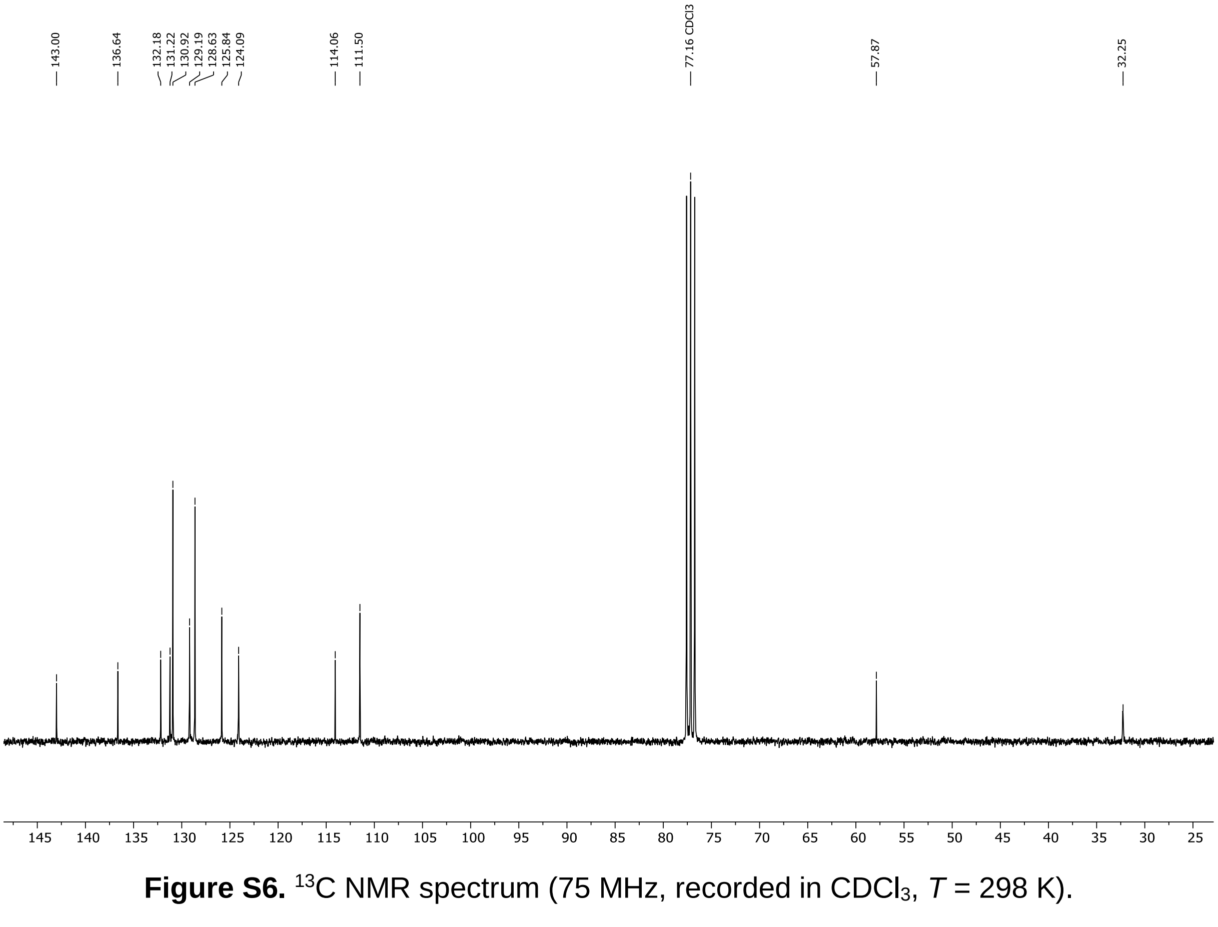

Figure S6. ^{13}C NMR spectrum (75 MHz, recorded in $CDCl_3$, T = 298 K).

4.4. 5-Fluoro-3-iodo-1-methyl-2-phenyl-1*H*-indole (5d)

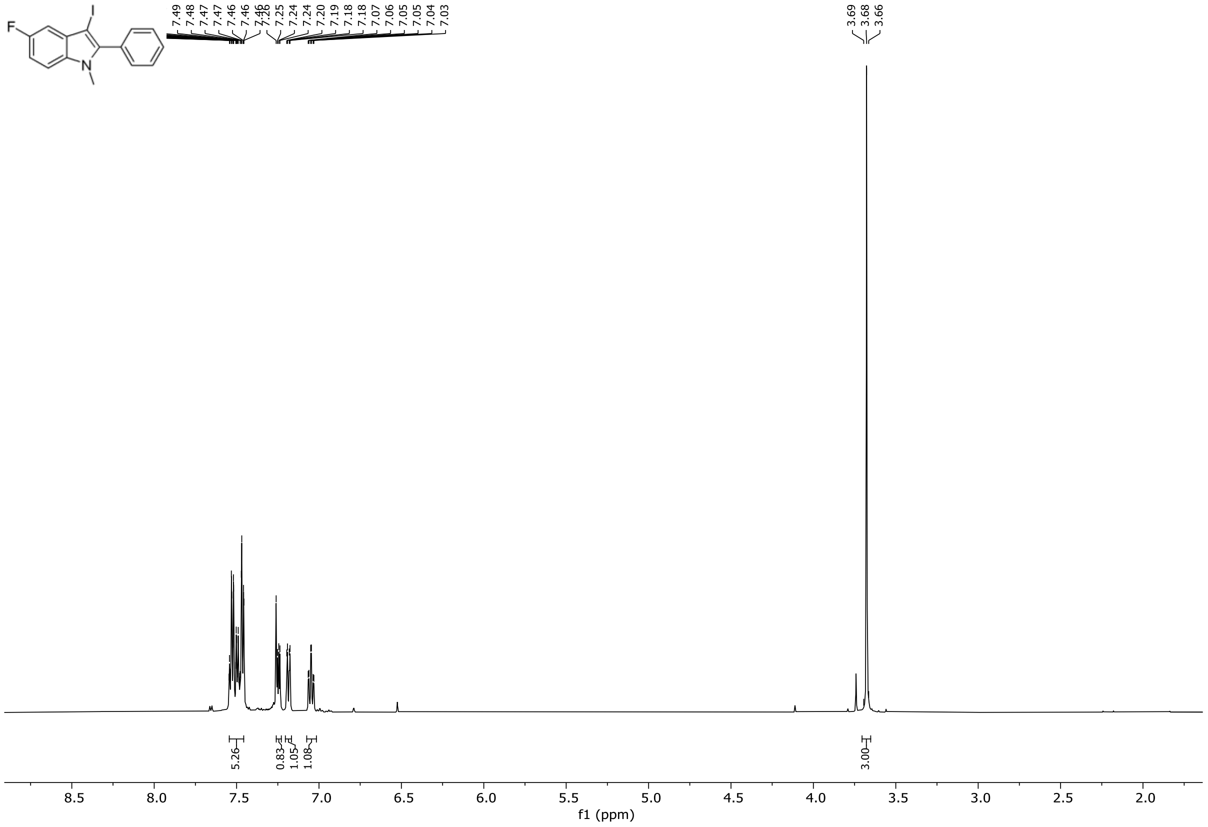

Figure S7. ^{1}H NMR spectrum (300 MHz, recorded in $CDCl_3$, T = 298 K).

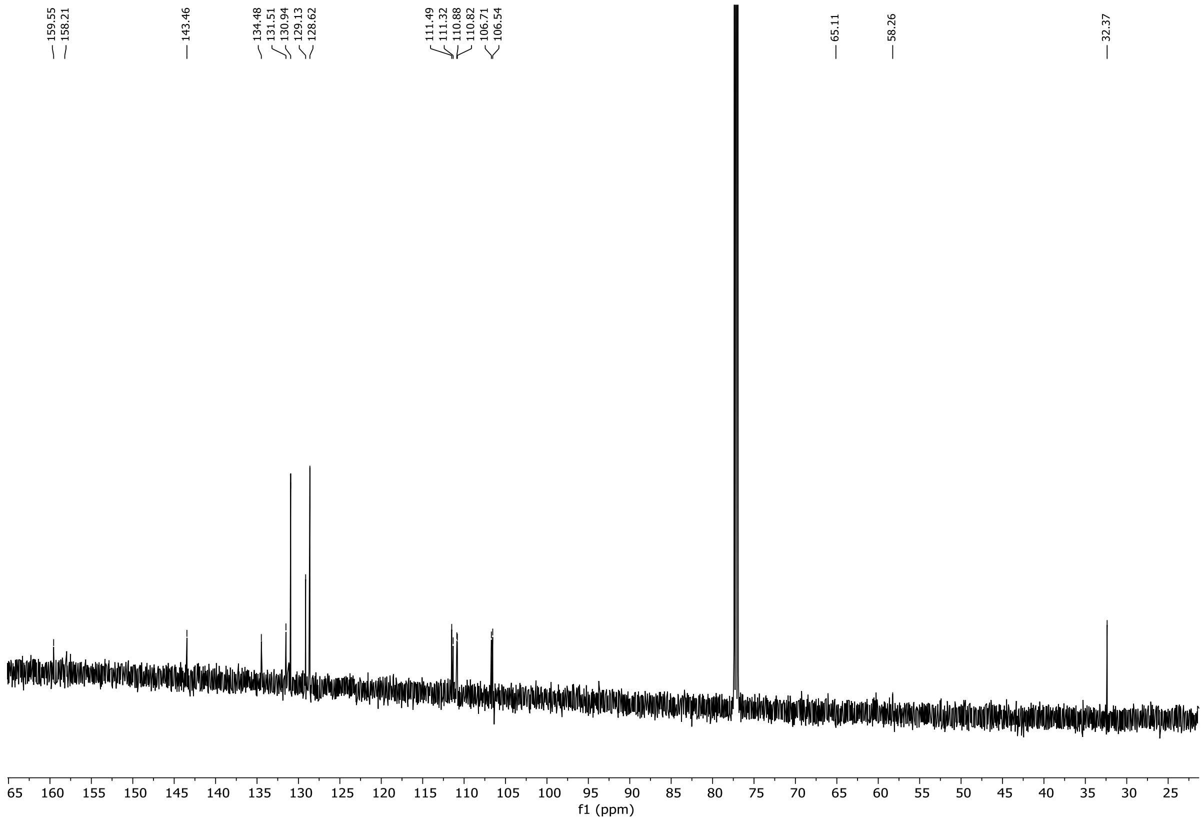

Figure S8. ^{13}C NMR spectrum (75 MHz, recorded in $CDCl_3$, T = 298 K).

4.5. 5-Chloro-3-iodo-1-methyl-2-phenyl-1*H*-indole (5e)

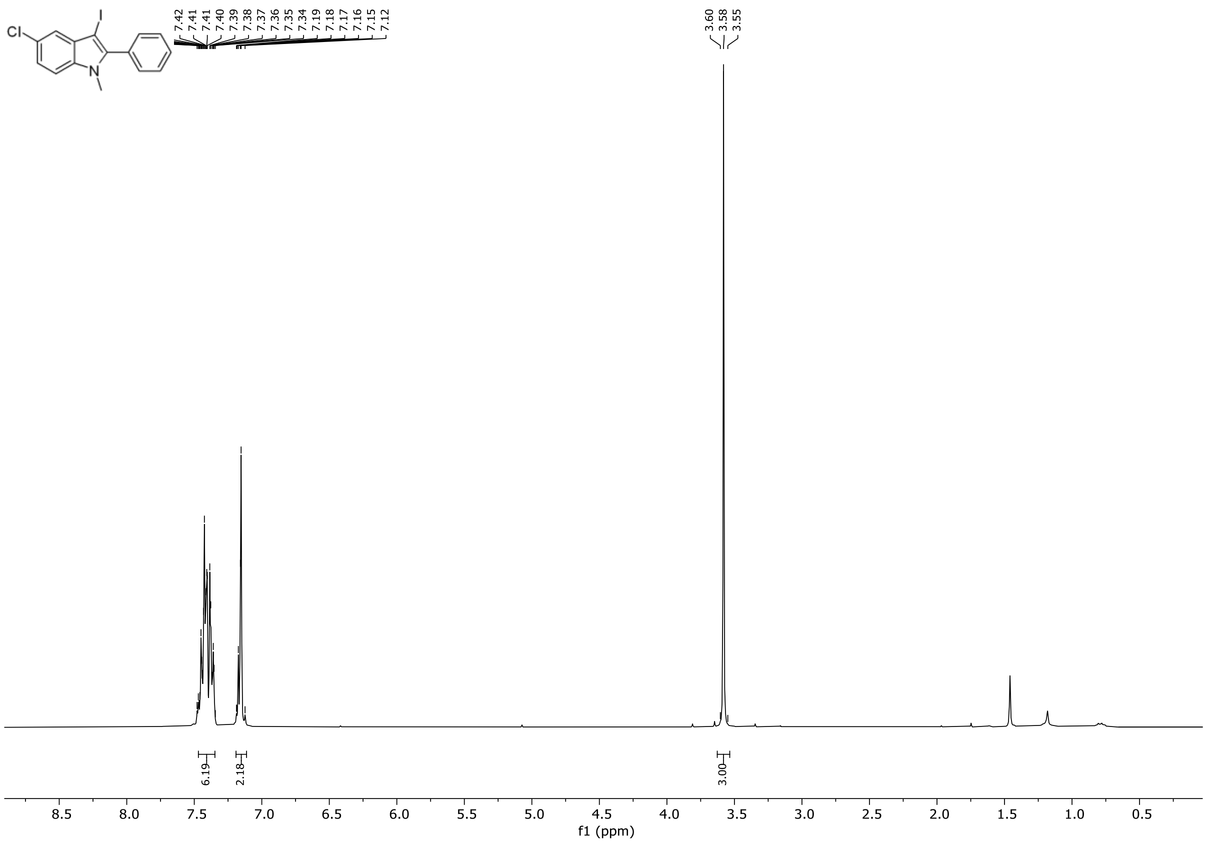

Figure S9. ^{1}H NMR spectrum (300 MHz, recorded in $CDCl_3$, T = 298 K).

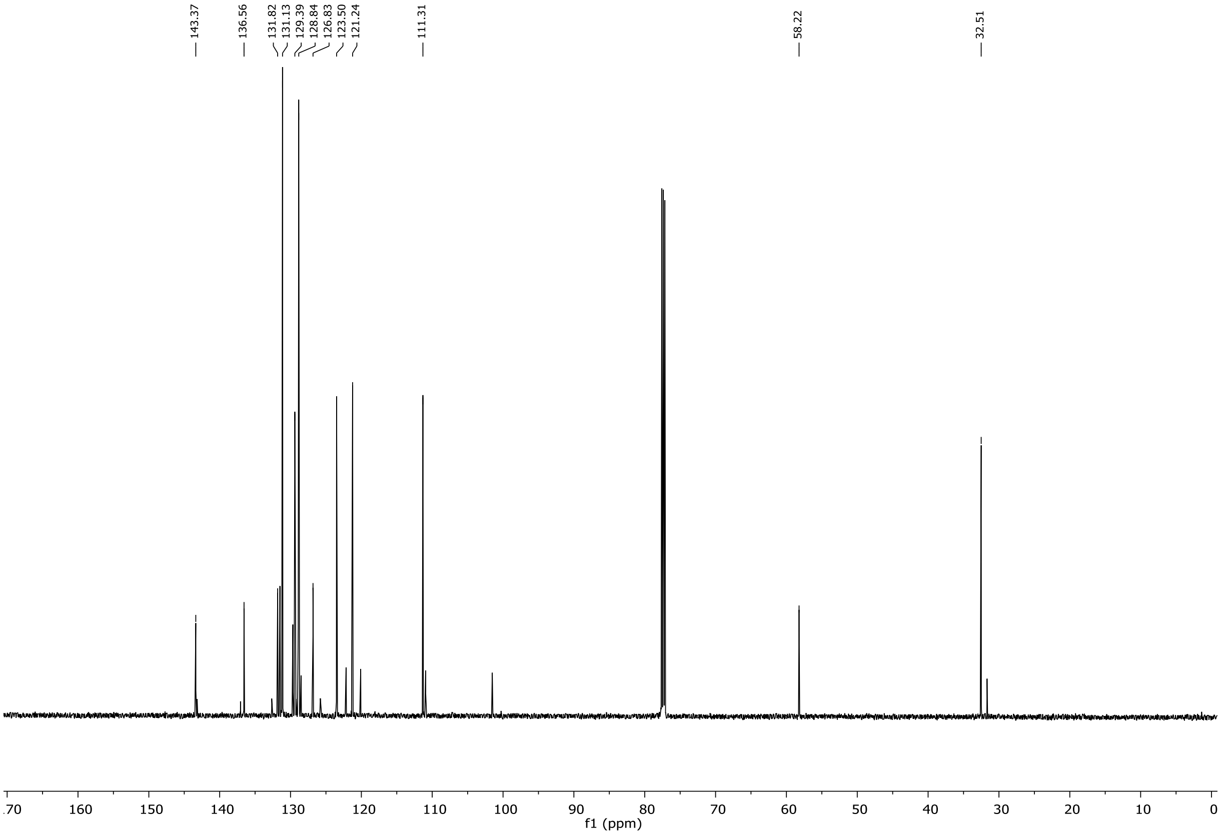

Figure S10. ^{13}C NMR spectrum (75 MHz, recorded in $CDCl_3$, T = 298 K).

4.6. 4-(3-Iodo-1-methyl-1*H*-indol-2-yl)-*N*,*N*-dimethylaniline (5f)[1]

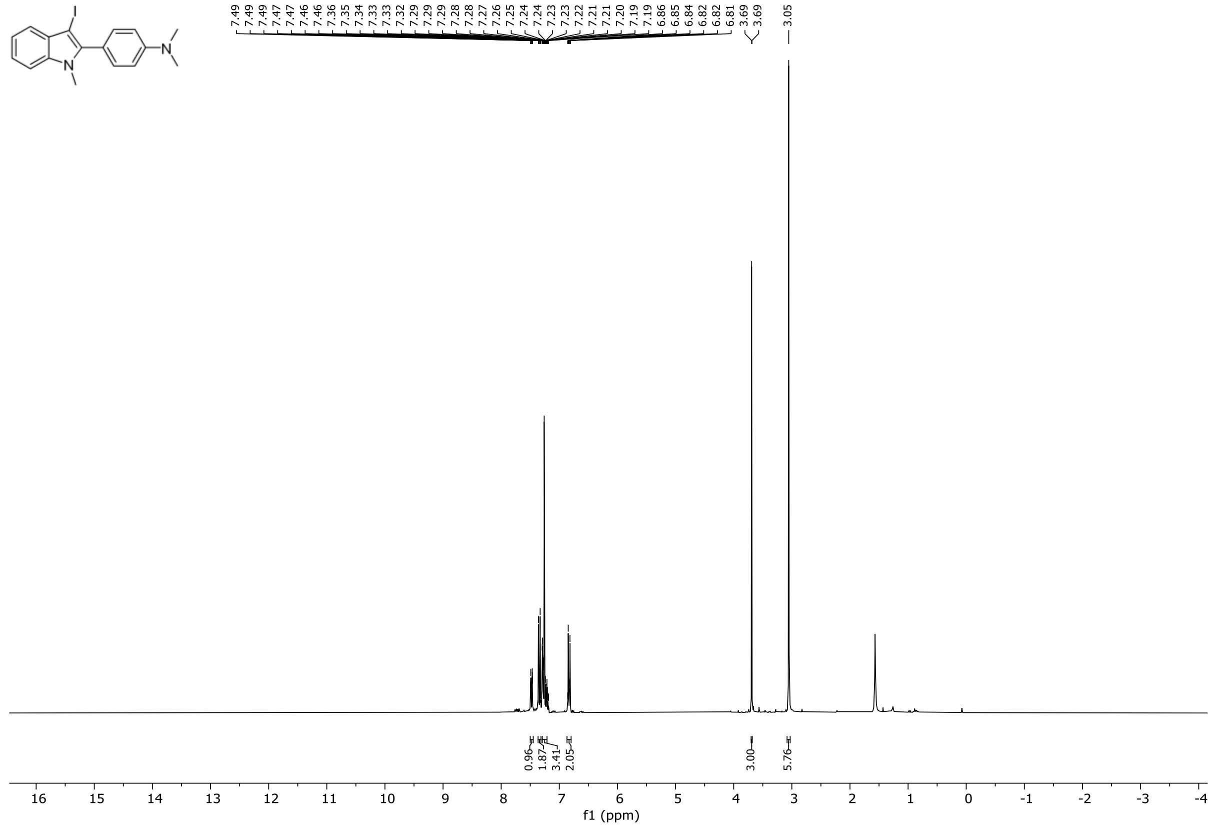

Figure S11. ^{1}H NMR spectrum (300 MHz, recorded in $CDCl_3$, T = 298 K).

4.7. 3-Iodo-2-(4-methoxyphenyl)-1-methyl-1*H*-indole (5g)

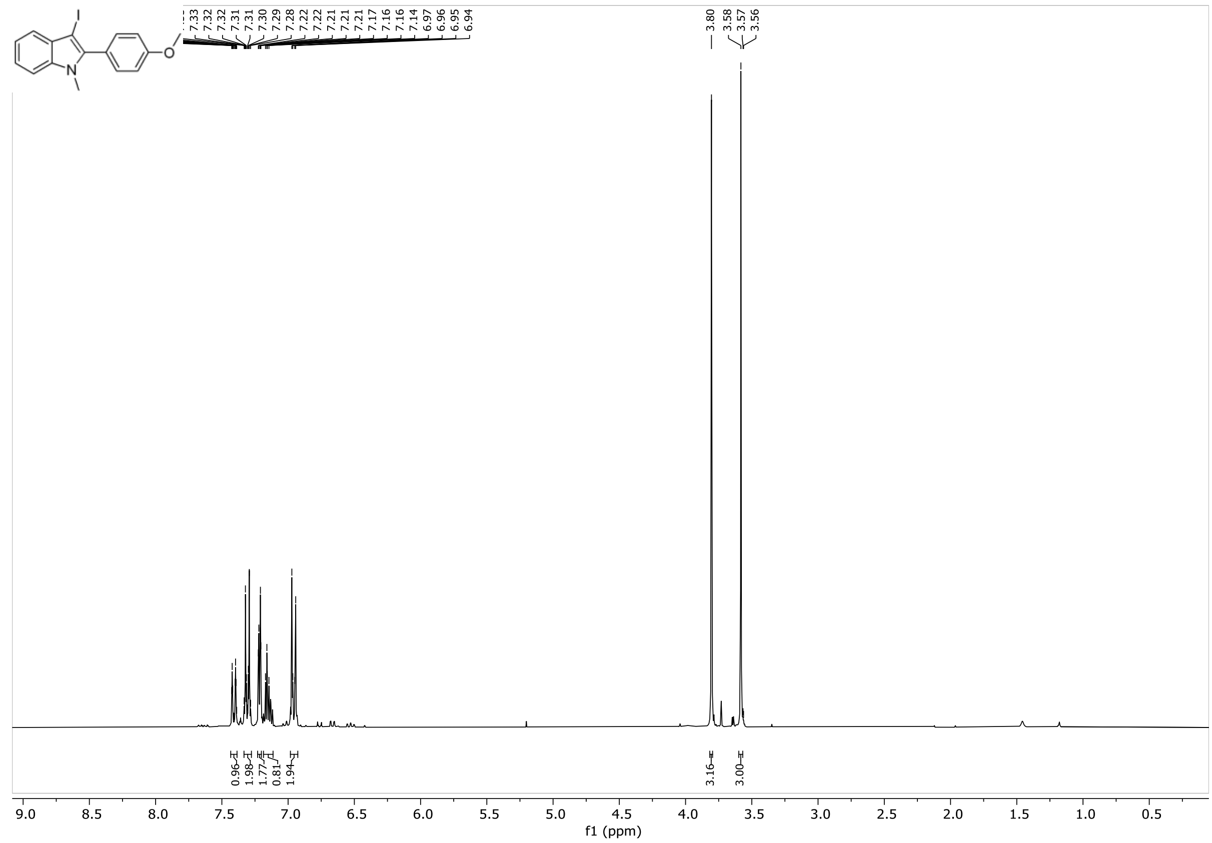

Figure S12. ^{1}H NMR spectrum (300 MHz, recorded in $CDCl_3$, T = 298 K).

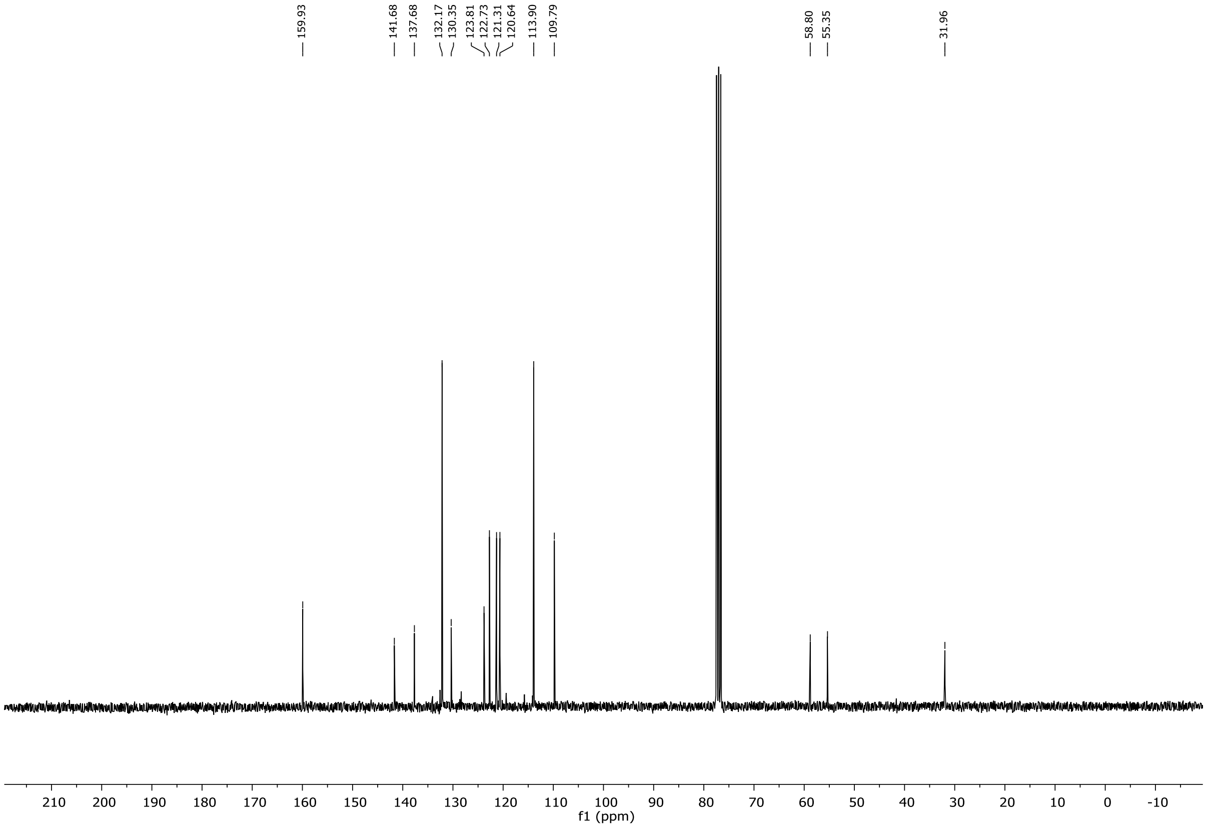

Figure S13. ^{13}C NMR spectrum (75 MHz, recorded in $CDCl_3$, T = 298 K).

4.8. 3-Iodo-1-methyl-2-(*p*-tolyl)-1*H*-indole (5h)

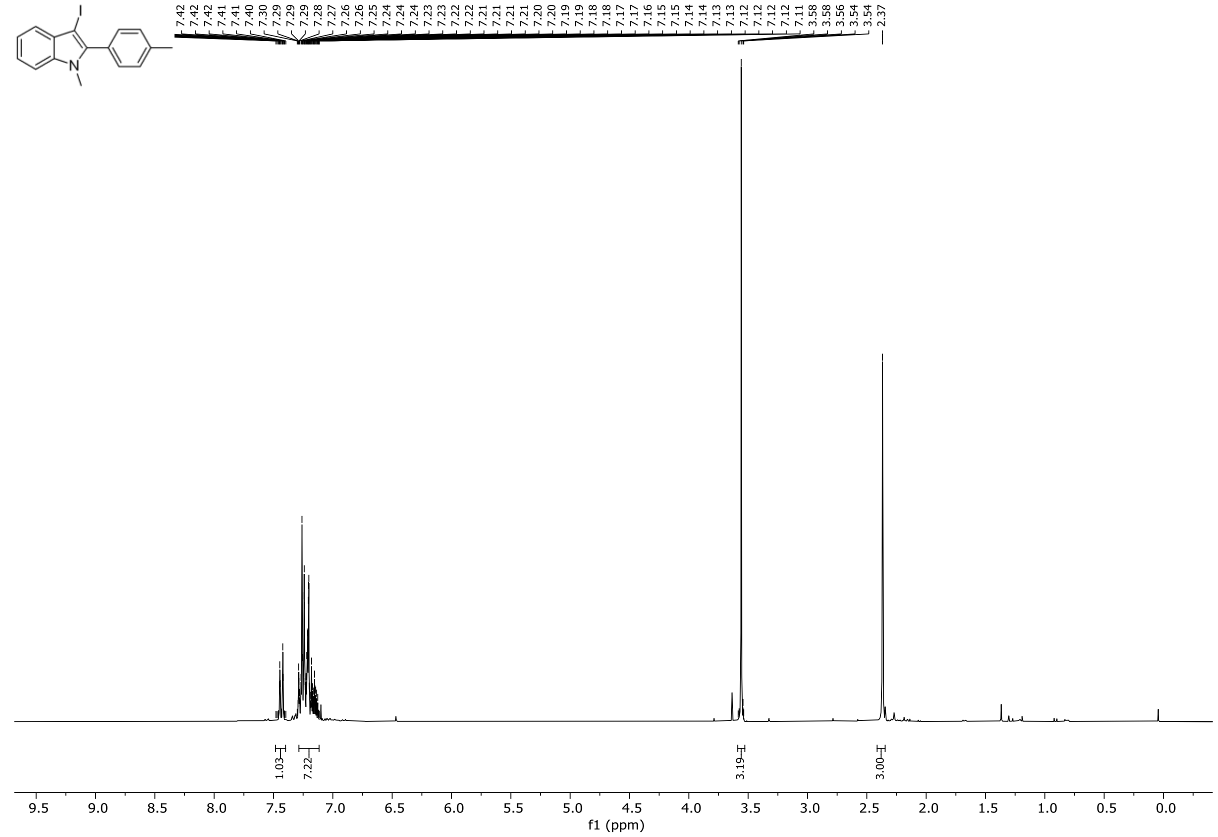

Figure S14. ^{1}H NMR spectrum (300 MHz, recorded in $CDCl_3$, T = 298 K).

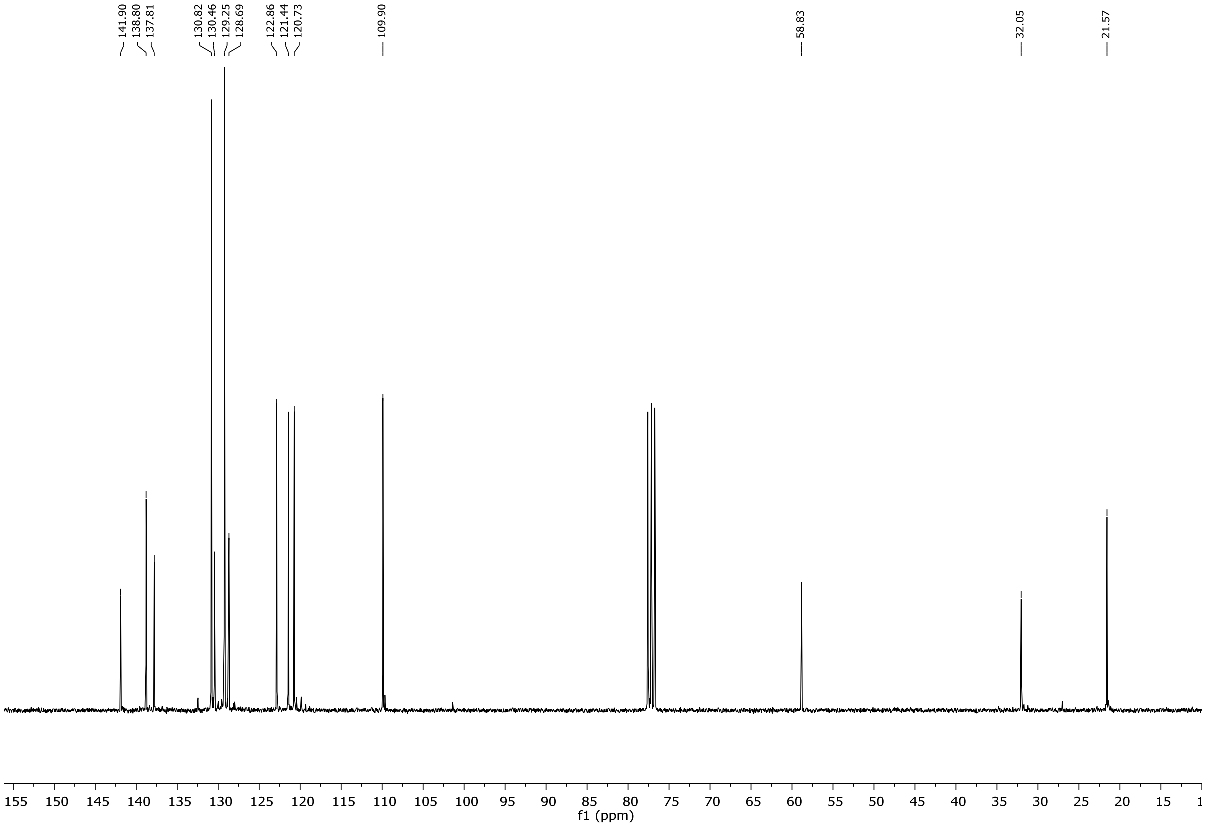

Figure S15. ^{13}C NMR spectrum (75 MHz, recorded in $CDCl_3$, T = 298 K).

4.9. 3-Iodo-1-methyl-2-(4-(trifluormethyl)phenyl)-1*H*-indole (5i)

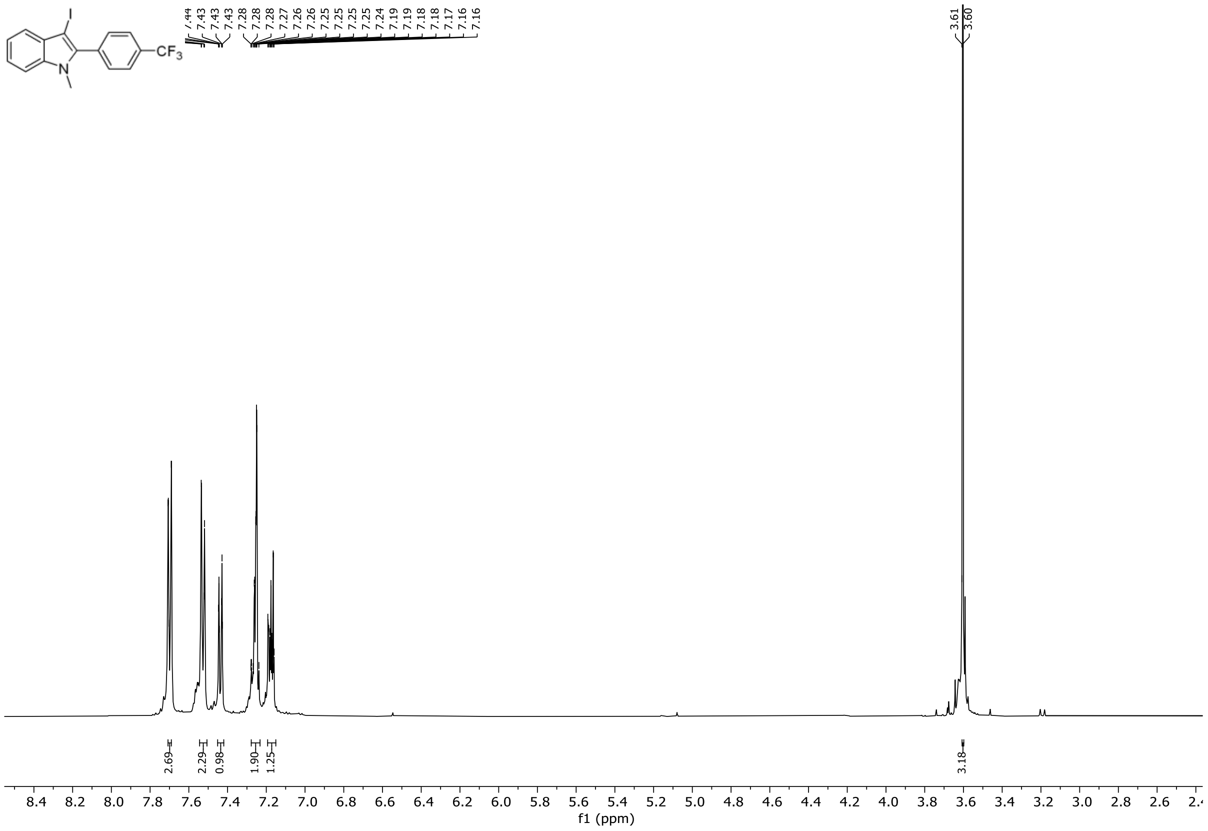

Figure S16. ^{1}H NMR spectrum (300 MHz, recorded in $CDCl_3$, T = 298 K).

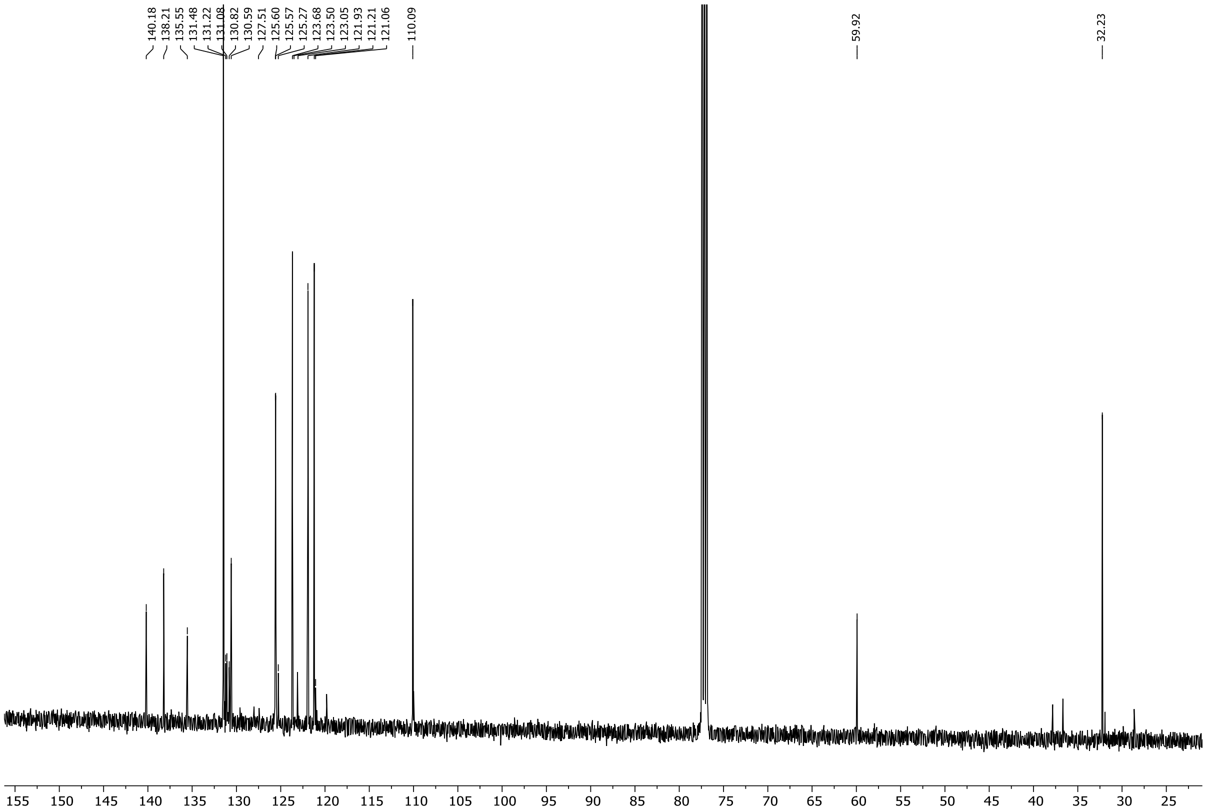

Figure S17. ^{13}C NMR spectrum (75 MHz, recorded in $CDCl_3$, T = 298 K).

4.10. 3-Iodo-2-(4-methoxyphenyl)-1,5-dimethyl-1*H*-indole (5j)

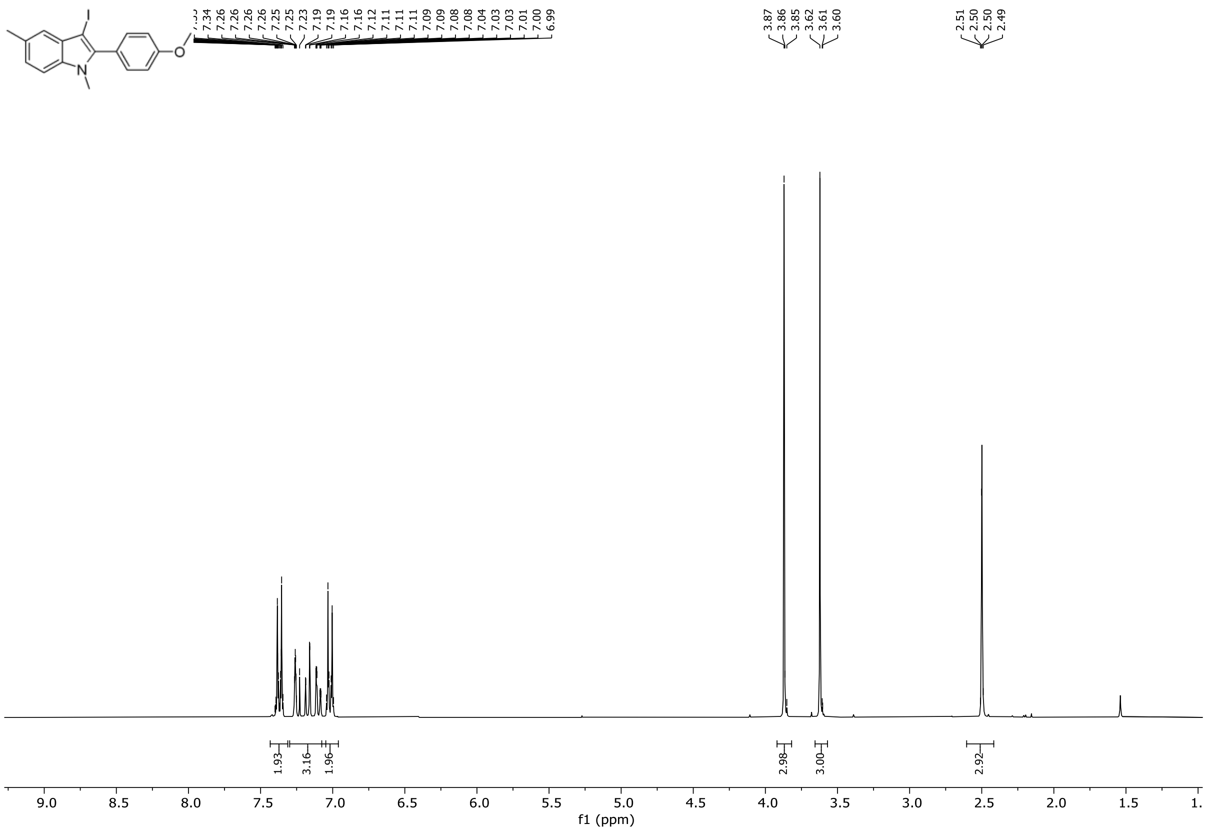

Figure S18. ^{1}H NMR spectrum (300 MHz, recorded in $CDCl_3$, T = 298 K).

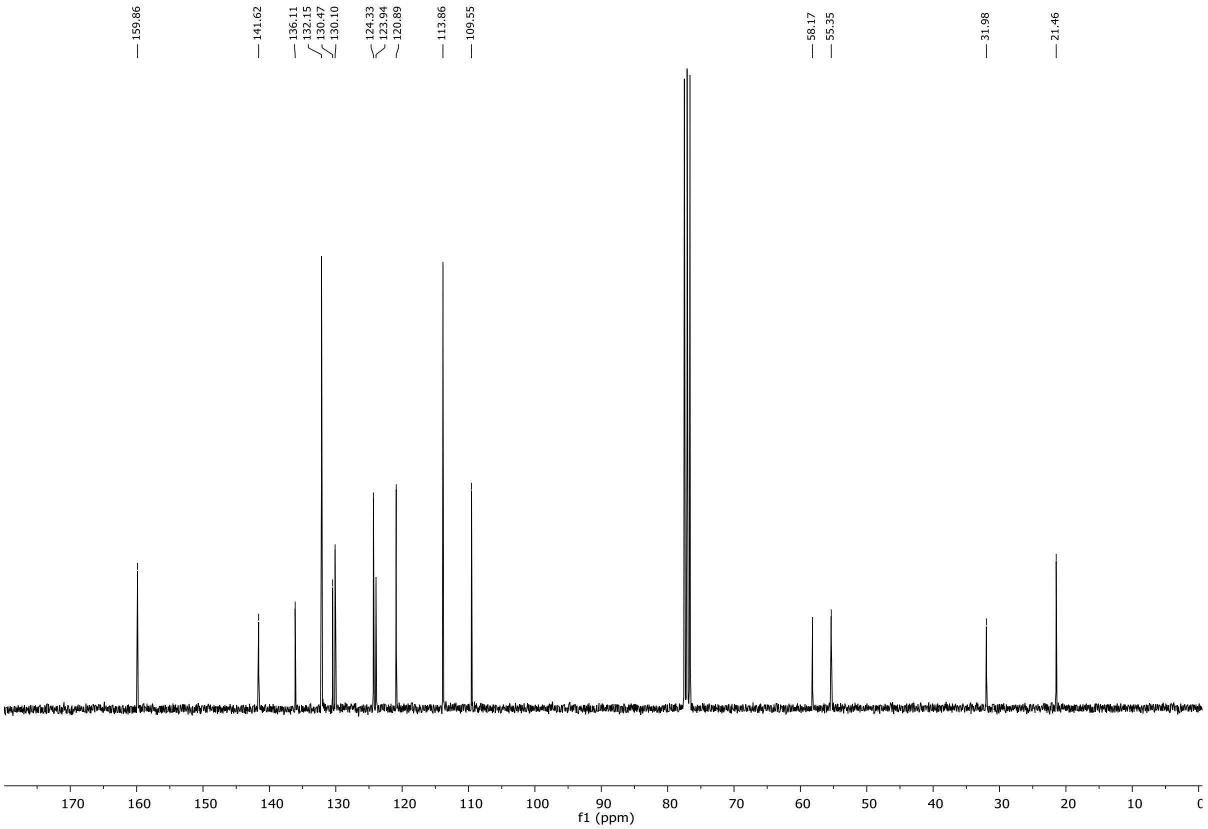

Figure S19. ^{13}C NMR spectrum (75 MHz, recorded in $CDCl_3$, T = 298 K).

4.11. 3-Iodo-1,5-dimethyl-2-(*p*-tolyl)-1*H*-indole (5k)

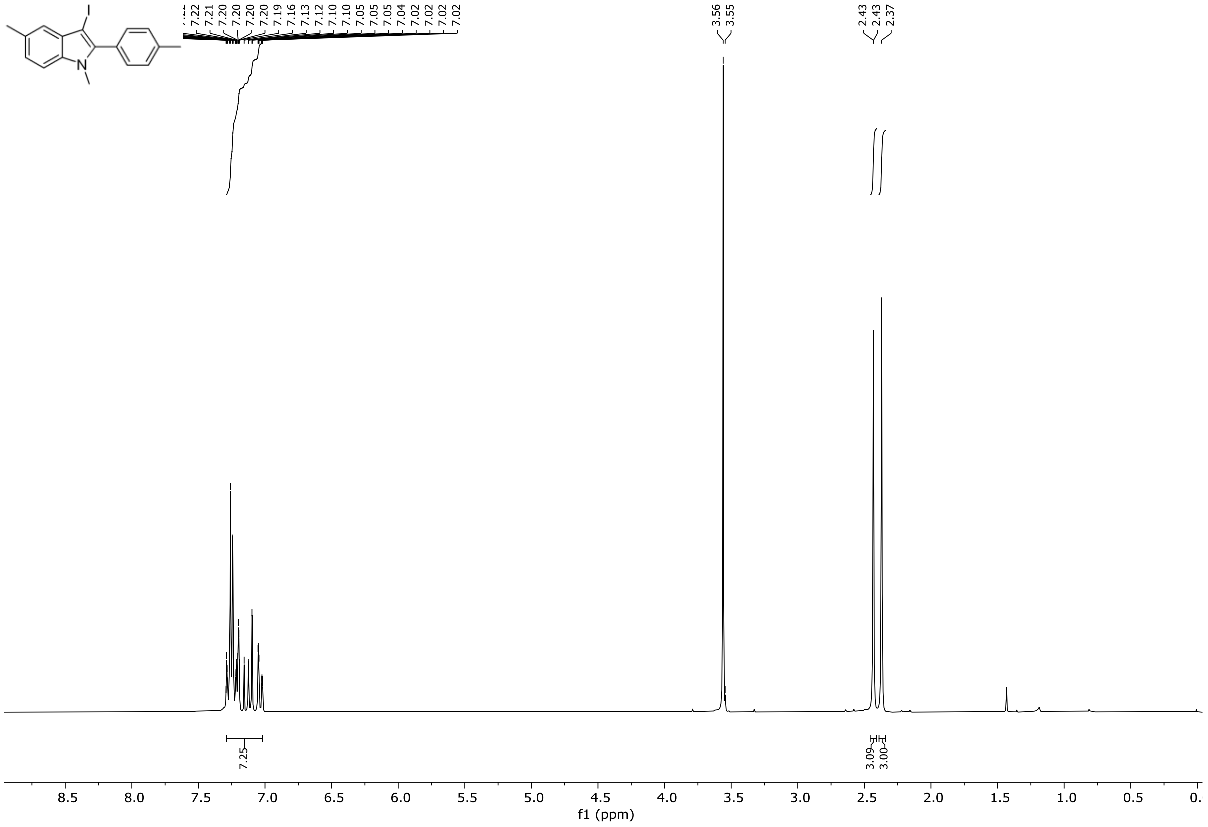

Figure S20. ^{1}H NMR spectrum (300 MHz, recorded in $CDCl_3$, T = 298 K).

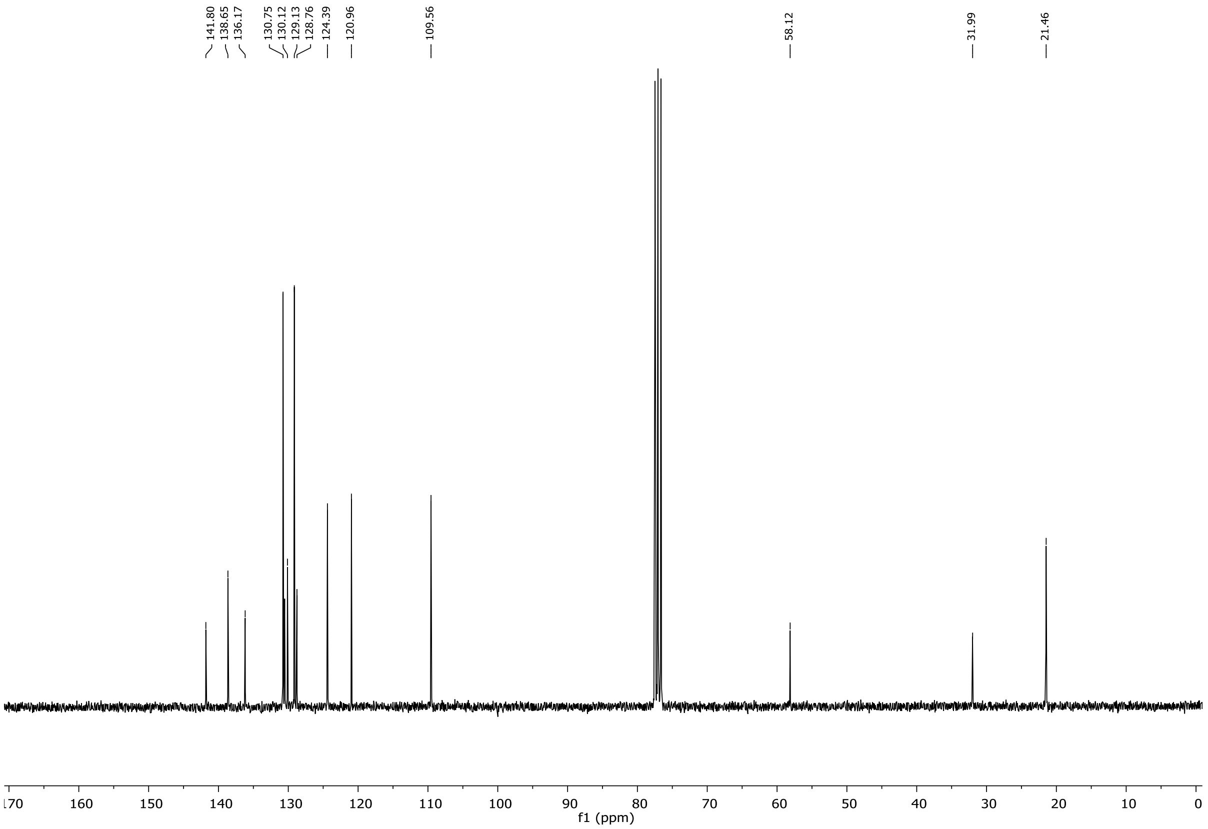

Figure S21. ^{13}C NMR spectrum (75 MHz, recorded in $CDCl_3$, T = 298 K).

4.12. 2-Cyclopropyl-3-iodo-1-methyl-1*H*-indole (5l)

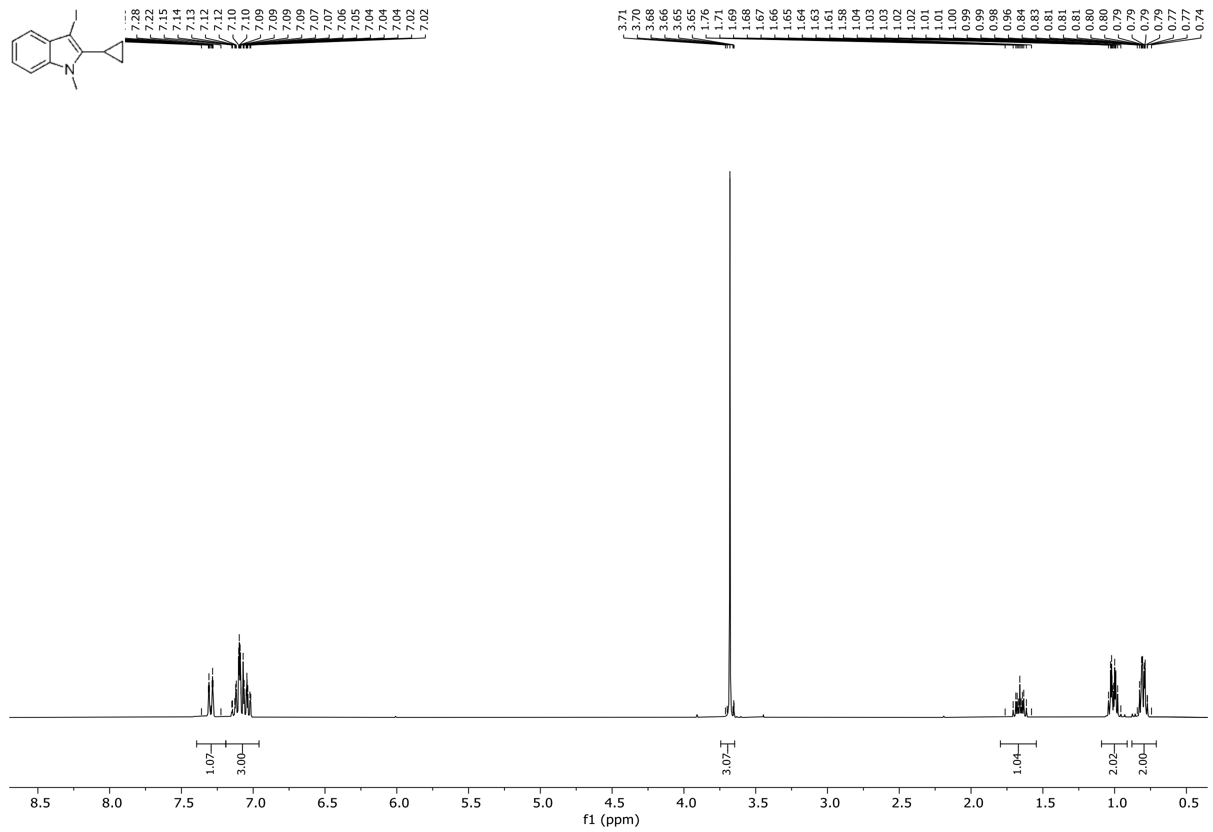

Figure S22. ^{1}H NMR spectrum (300 MHz, recorded in $CDCl_3$, T = 298 K).

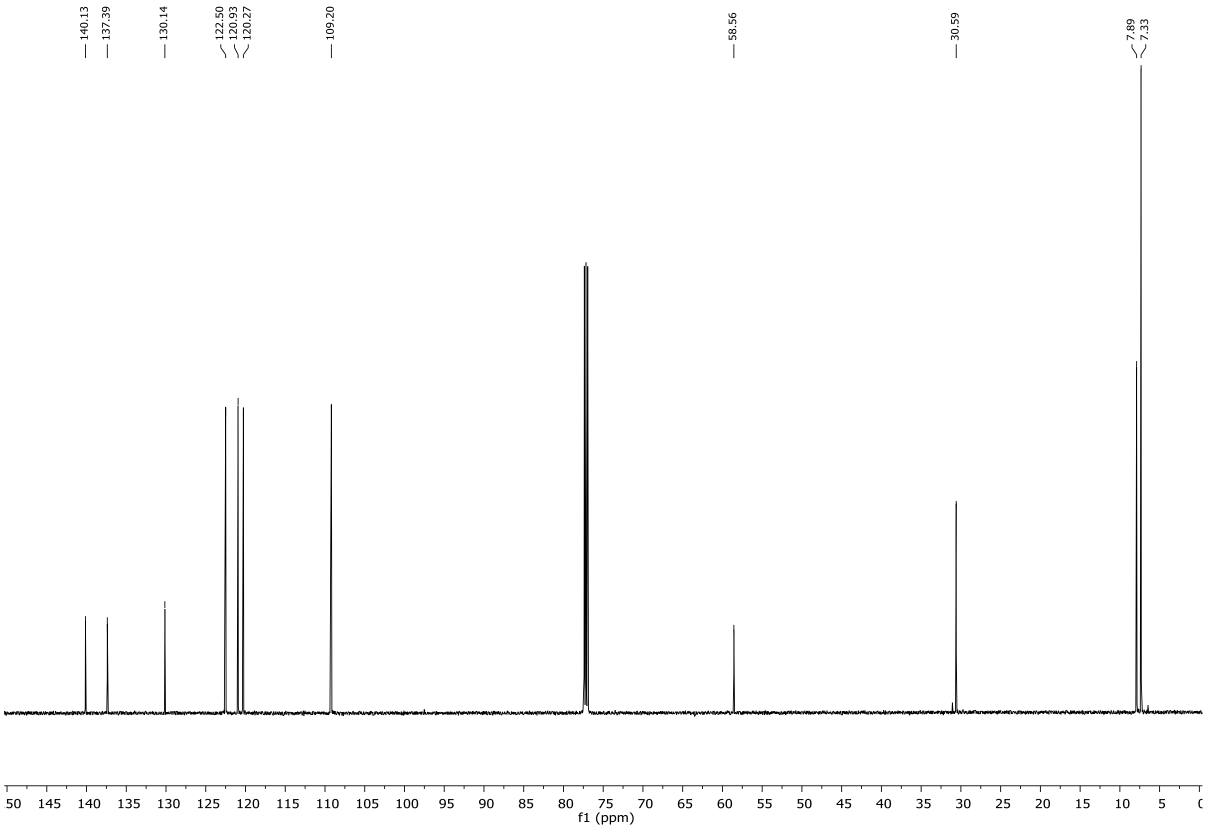

Figure S23. ^{13}C NMR spectrum (75 MHz, recorded in $CDCl_3$, T = 298 K).

4.13. 2-Butyl-3-iodo-1-methyl-1*H*-indole (5m)

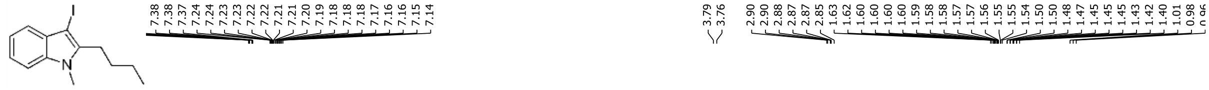

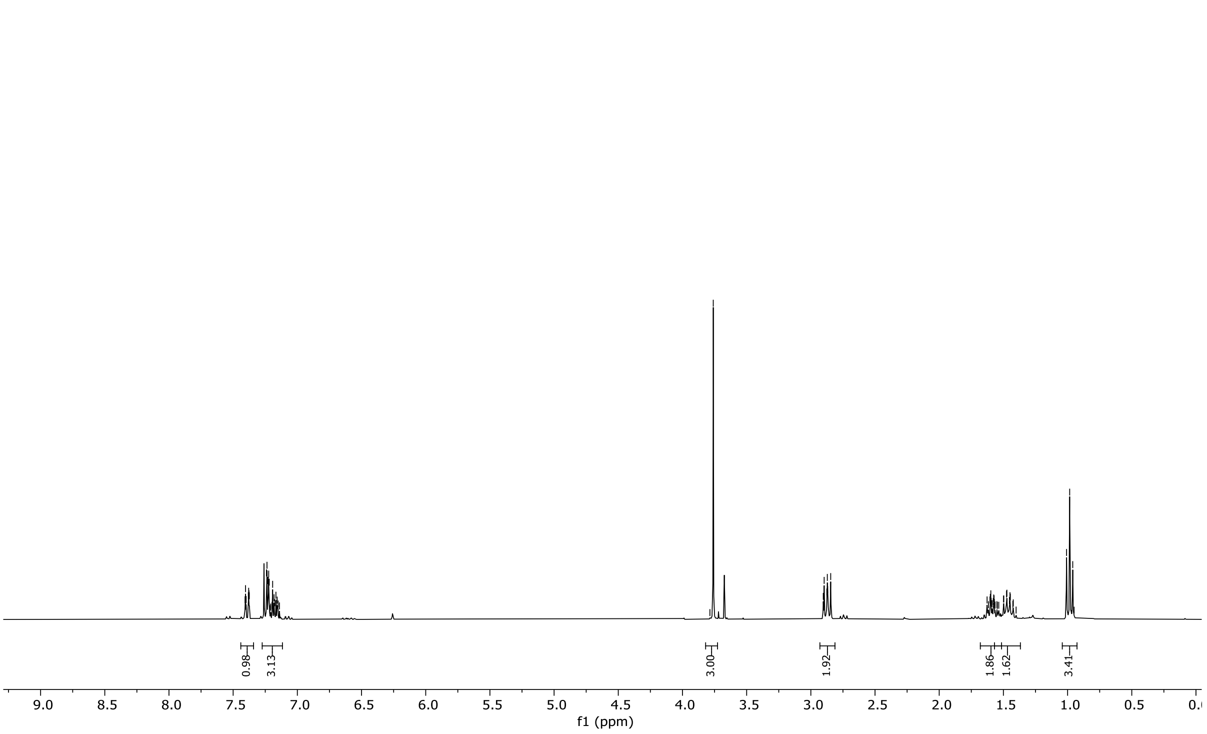

Figure S24. ^{1}H NMR spectrum (300 MHz, recorded in $CDCl_3$, T = 298 K).

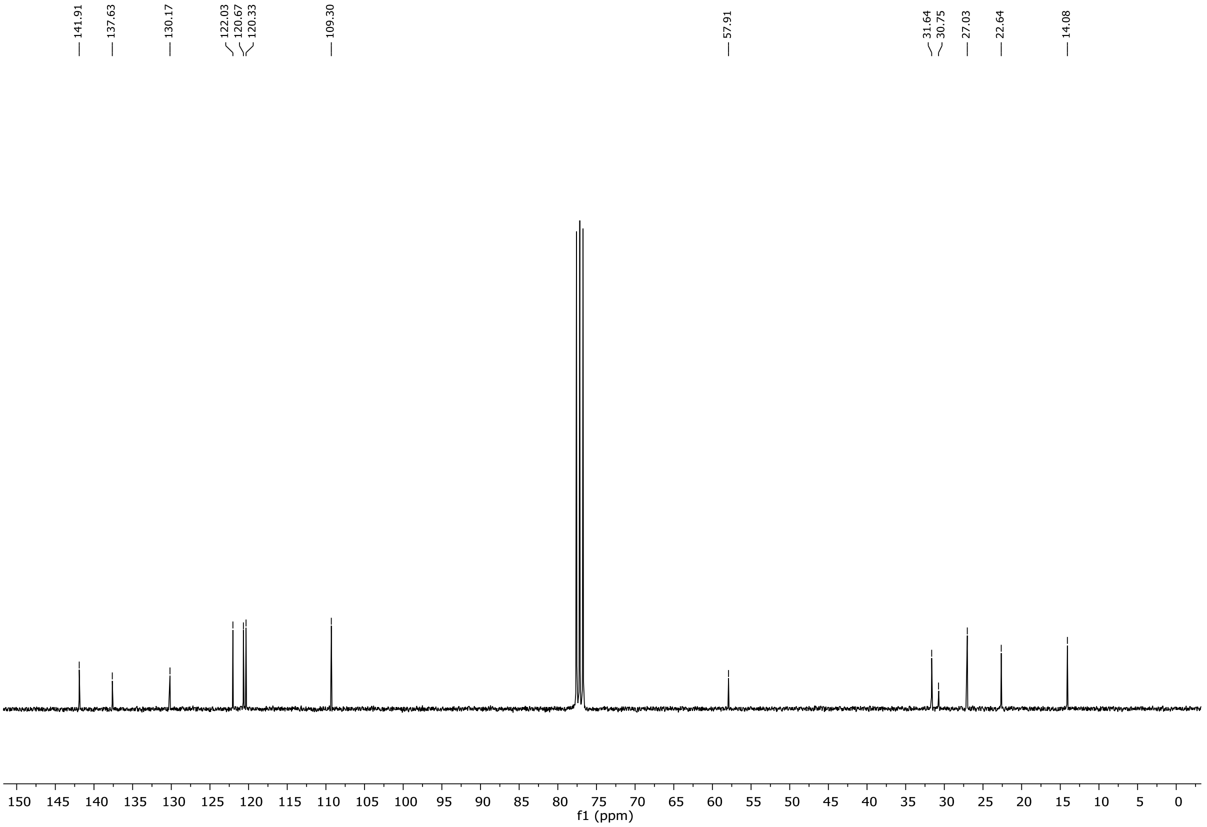

Figure S25. ^{13}C NMR spectrum (75 MHz, recorded in $CDCl_3$, T = 298 K).

4.14. 2-Butyl-3-iodo-1,5-dimethyl-1*H*-indole (5n)

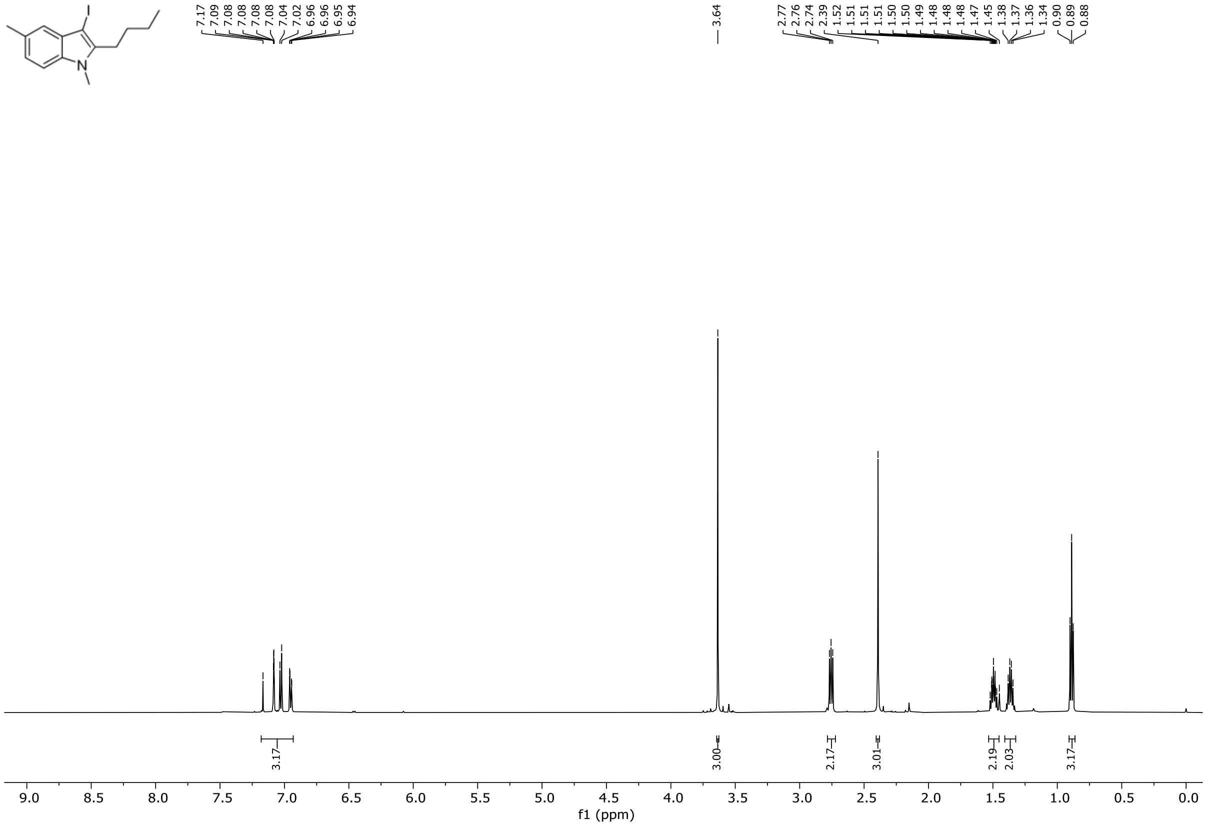

Figure S26. ^{1}H NMR spectrum (300 MHz, recorded in $CDCl_3$, T = 298 K).

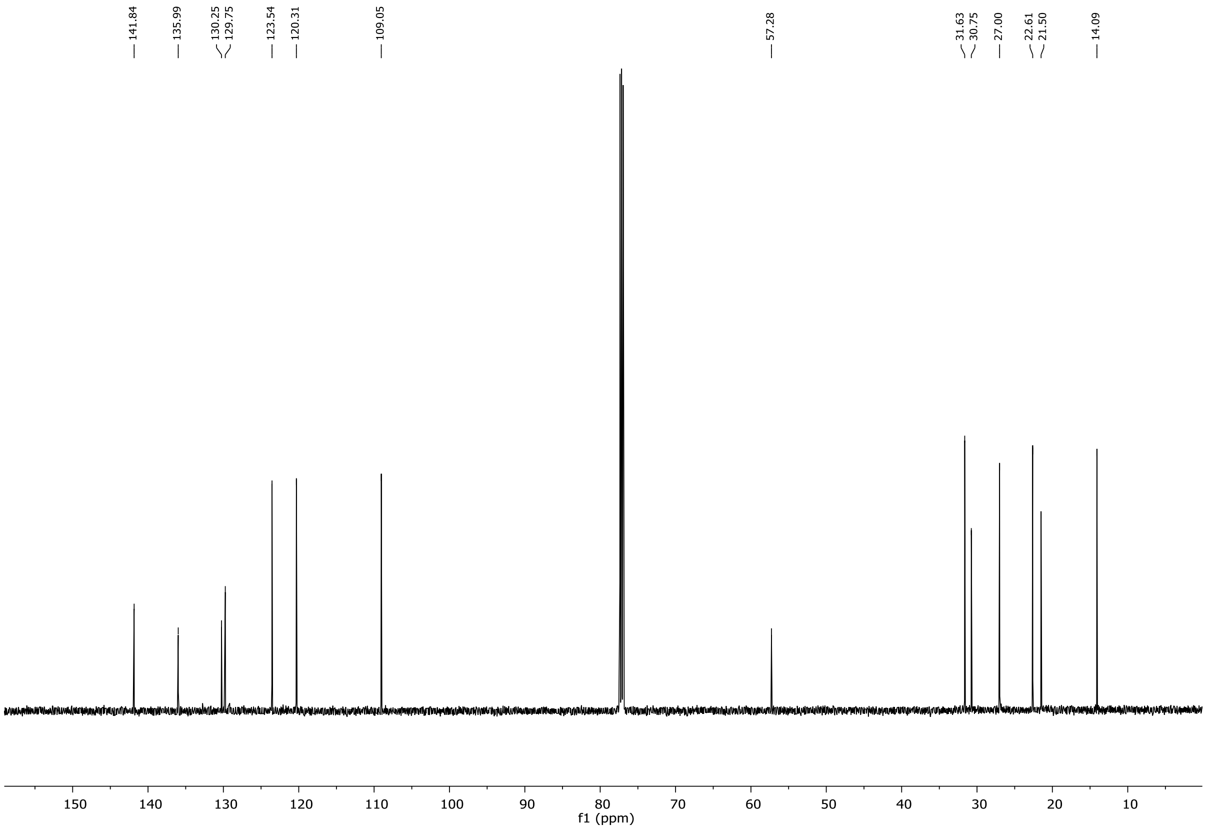

Figure S27. ^{13}C NMR spectrum (75 MHz, recorded in $CDCl_3$, T = 298 K).

4.15. 1-Benzyl-3-iodo-2-phenyl-1*H*-indole (5o)

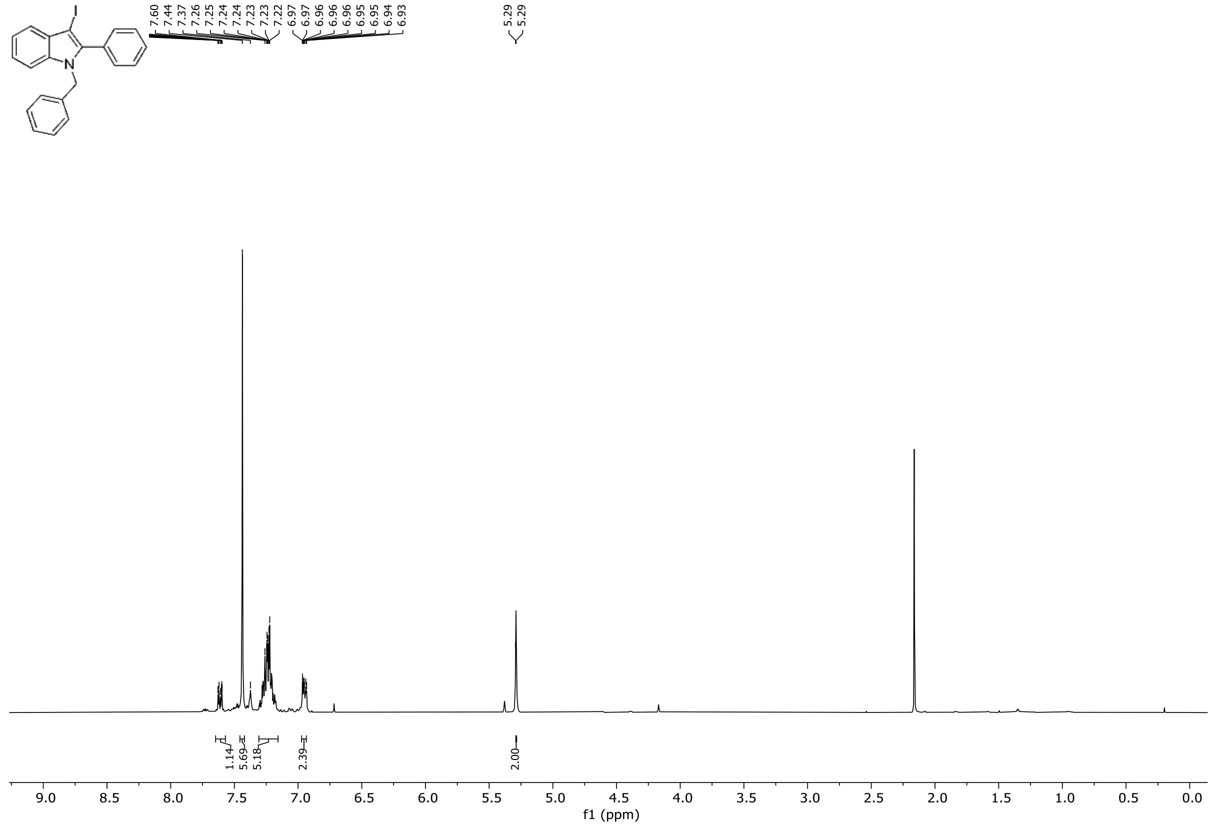

Figure S28. ^{1}H NMR spectrum (300 MHz, recorded in $CDCl_3$, T = 298 K).

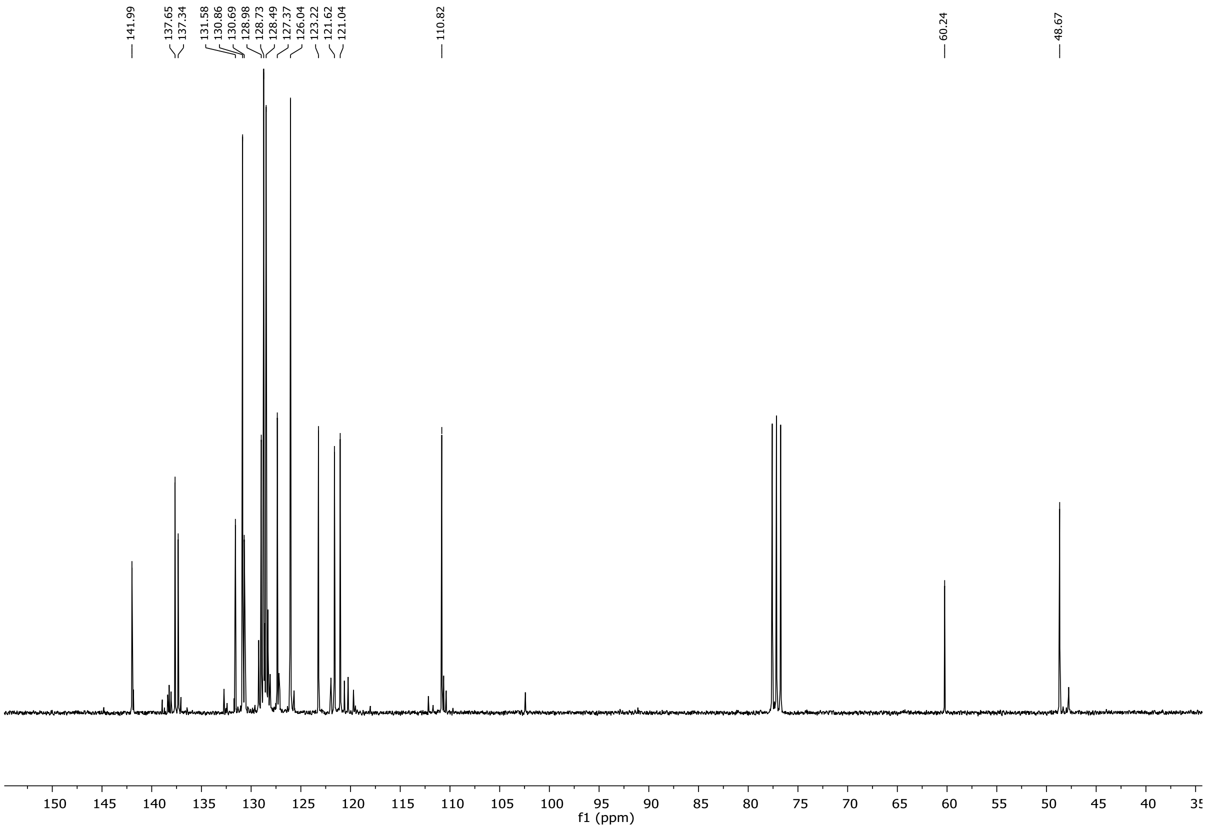

Figure S29. ^{13}C NMR spectrum (75 MHz, recorded in $CDCl_3$, T = 298 K).

4.16. 1-Benzyl-3-iodo-5-methyl-2-phenyl-1*H*-indole (5p)

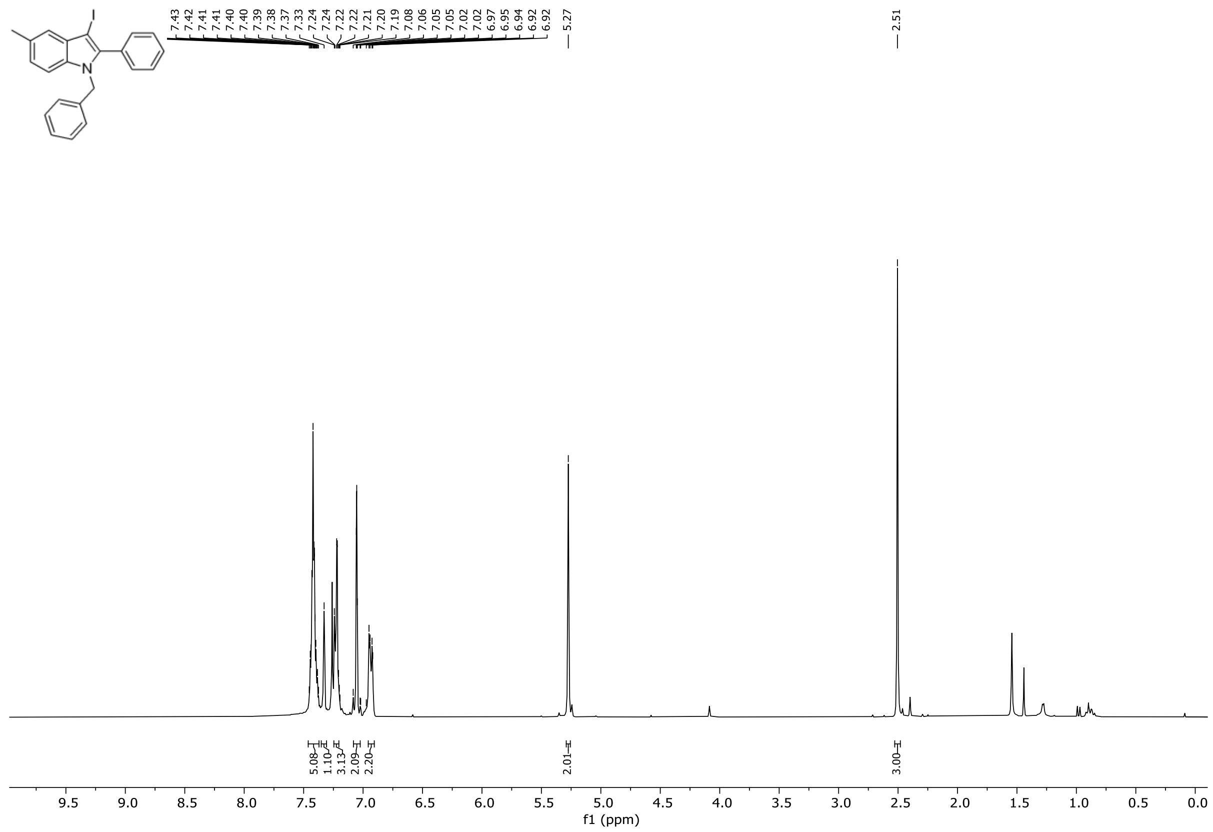

Figure S30. ^{1}H NMR spectrum (300 MHz, recorded in $CDCl_3$, *T* = 298 K).

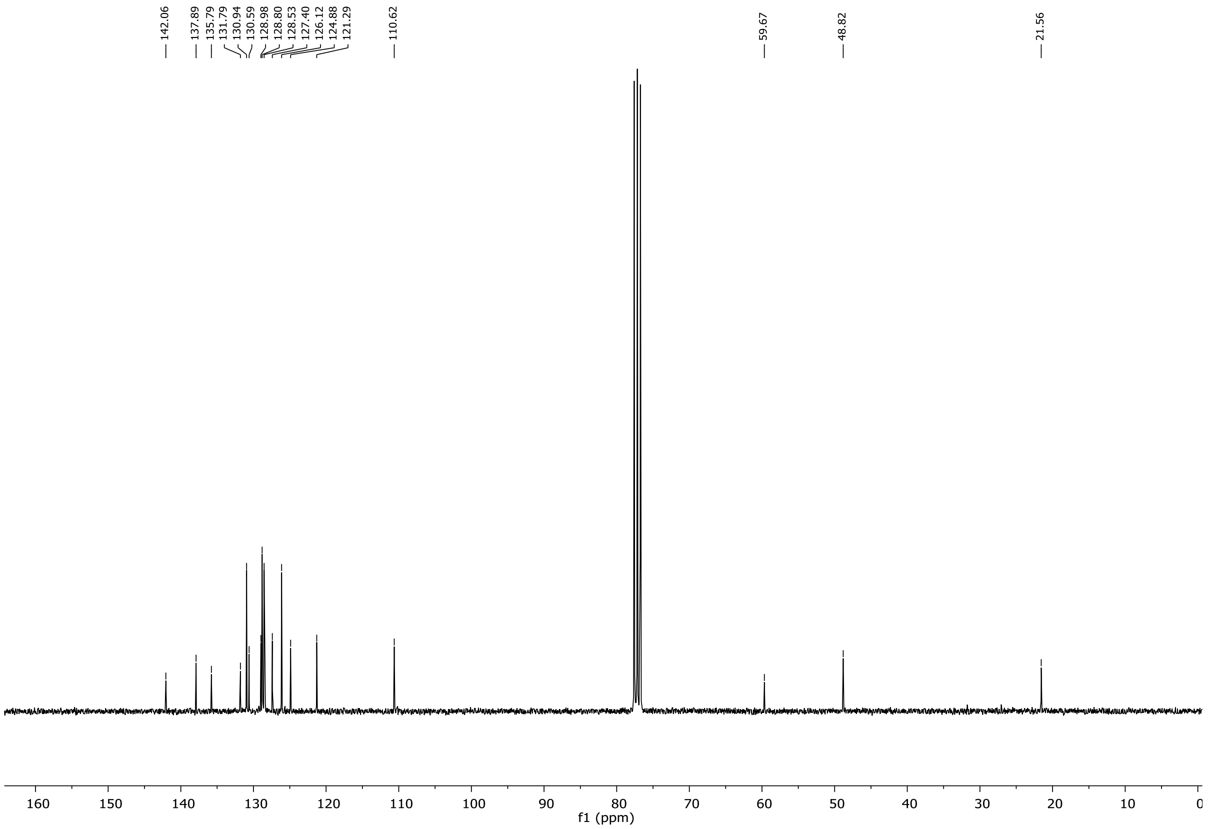

Figure S31. ^{13}C NMR spectrum (75 MHz, recorded in $CDCl_3$, *T* = 298 K).

4.17. 1-Benzyl-5-bromo-3-iodo-2-phenyl-1*H*-indole (5q)

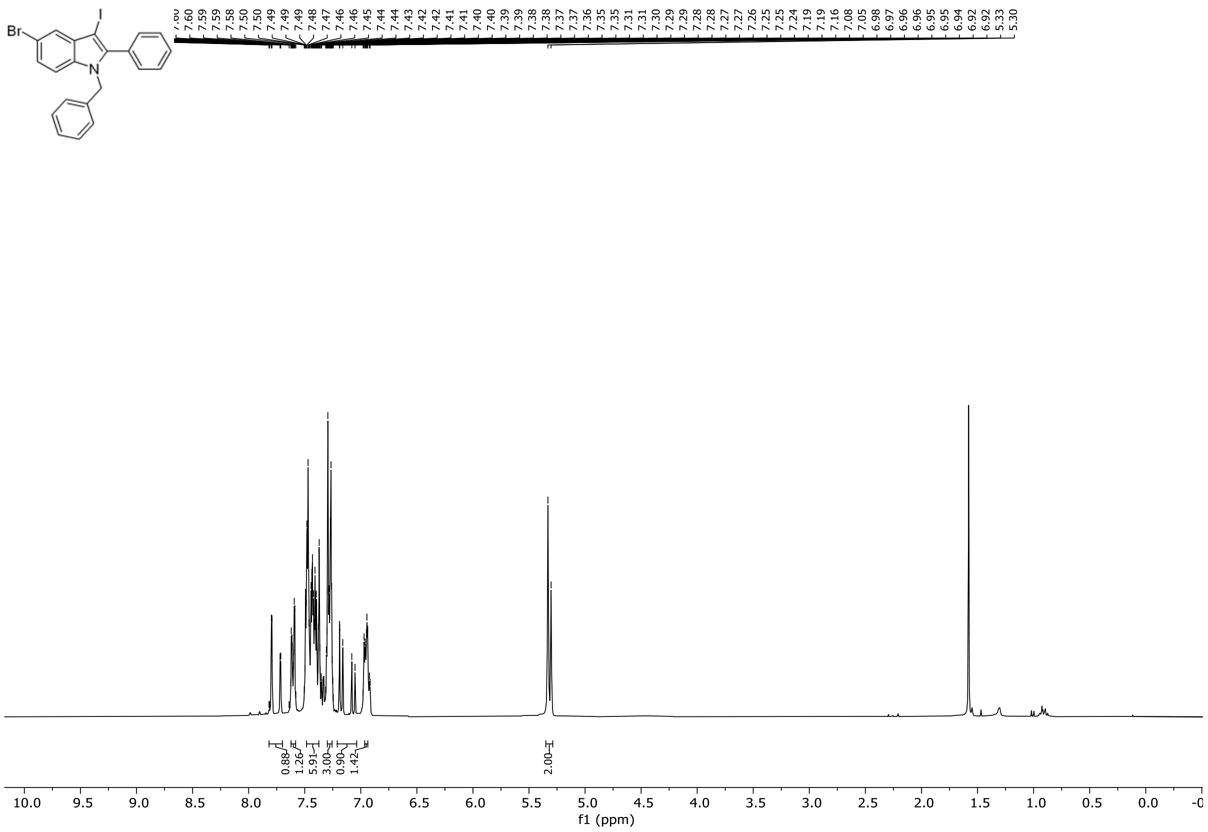

Figure S32. ^{1}H NMR spectrum (300 MHz, recorded in $CDCl_3$, T = 298 K).

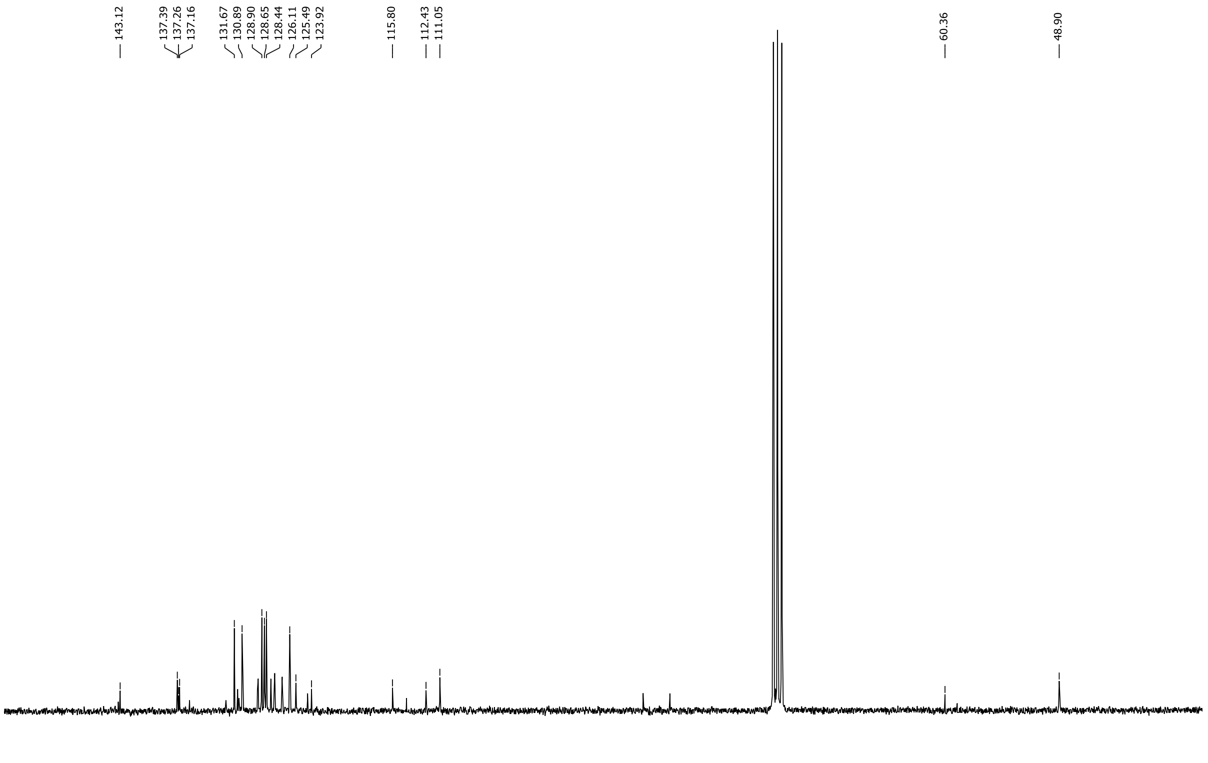

Figure S33. ^{13}C NMR spectrum (75 MHz, recorded in $CDCl_3$, T = 298 K).

4.18. 1-(4-Bromobenzyl)-3-iodo-2-phenyl-1*H*-indole (5r)

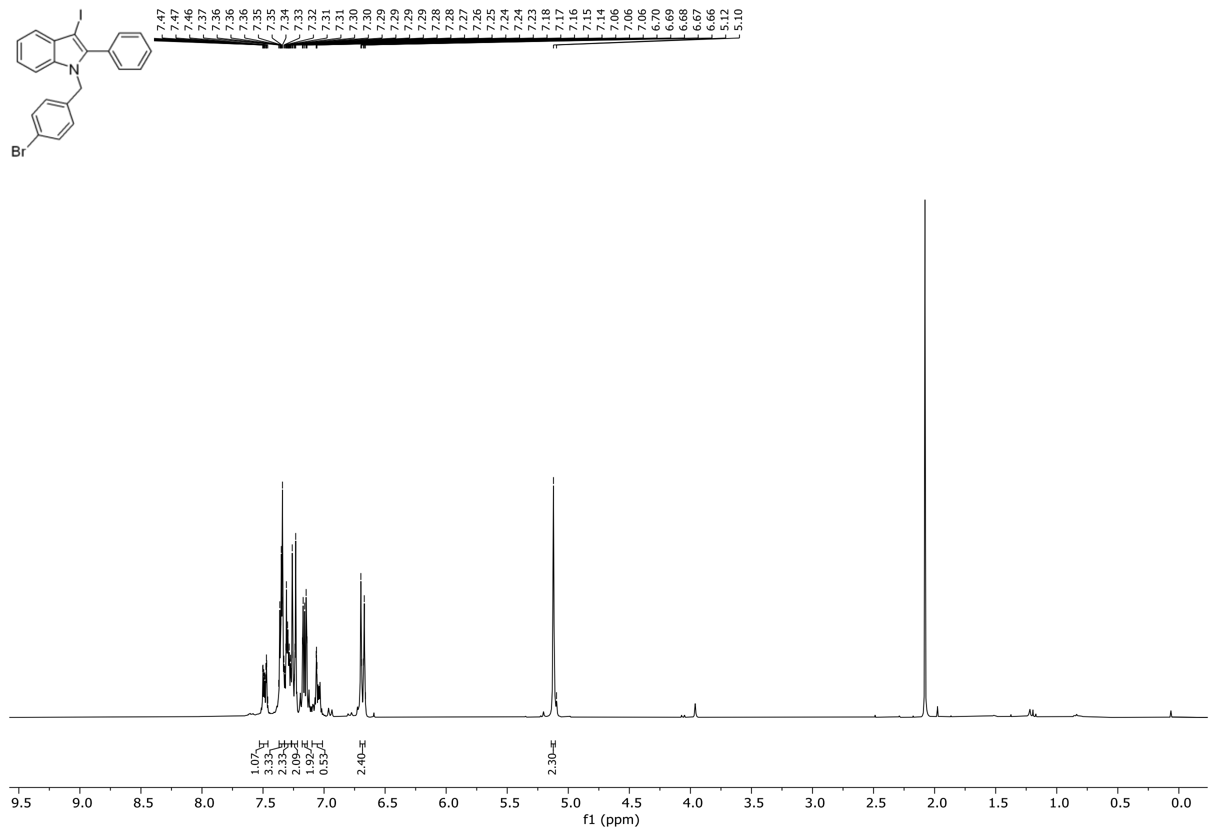

Figure S34. ^{1}H NMR spectrum (300 MHz, recorded in $CDCl_3$, *T* = 298 K).

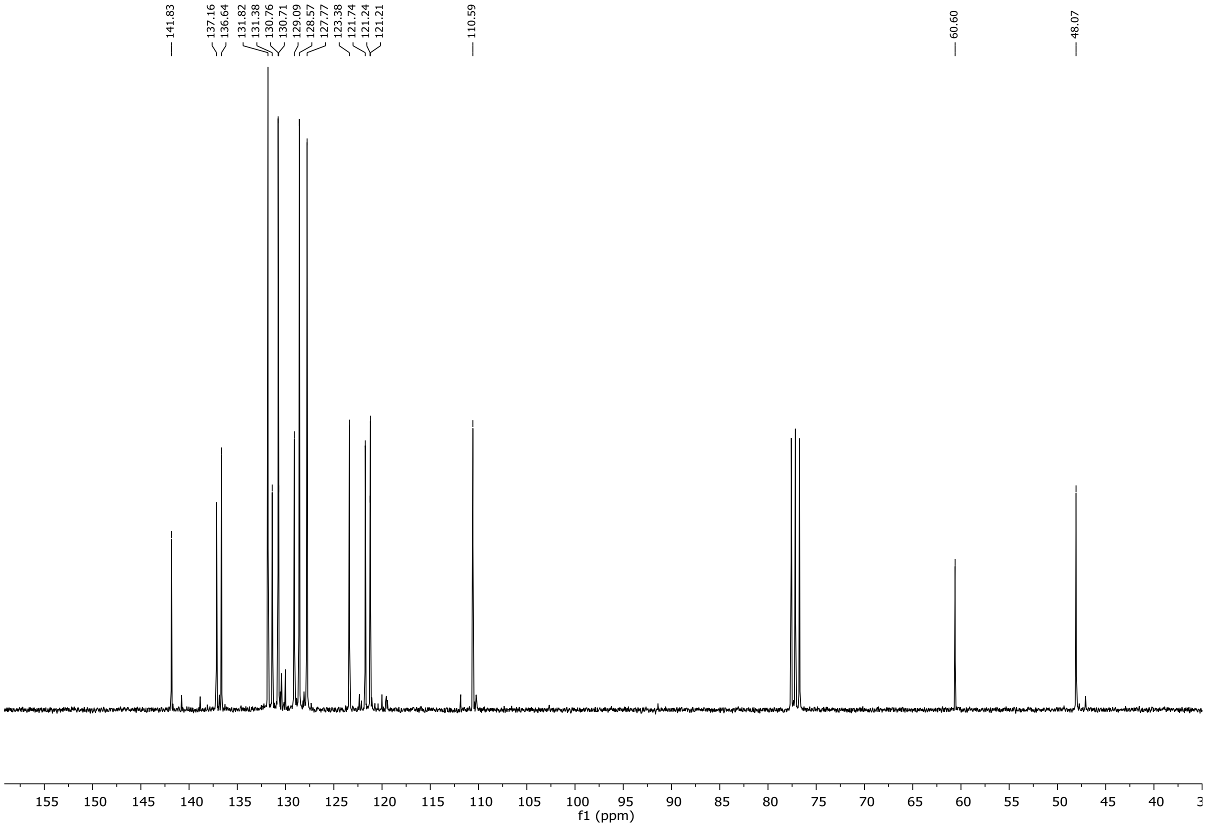

Figure S35. ^{13}C NMR spectrum (75 MHz, recorded in $CDCl_3$, *T* = 298 K).

4.19. Synthesis of 3-iodo-1-methyl-2-phenyl-5-(phenylethynyl)-1*H*-indole (6)

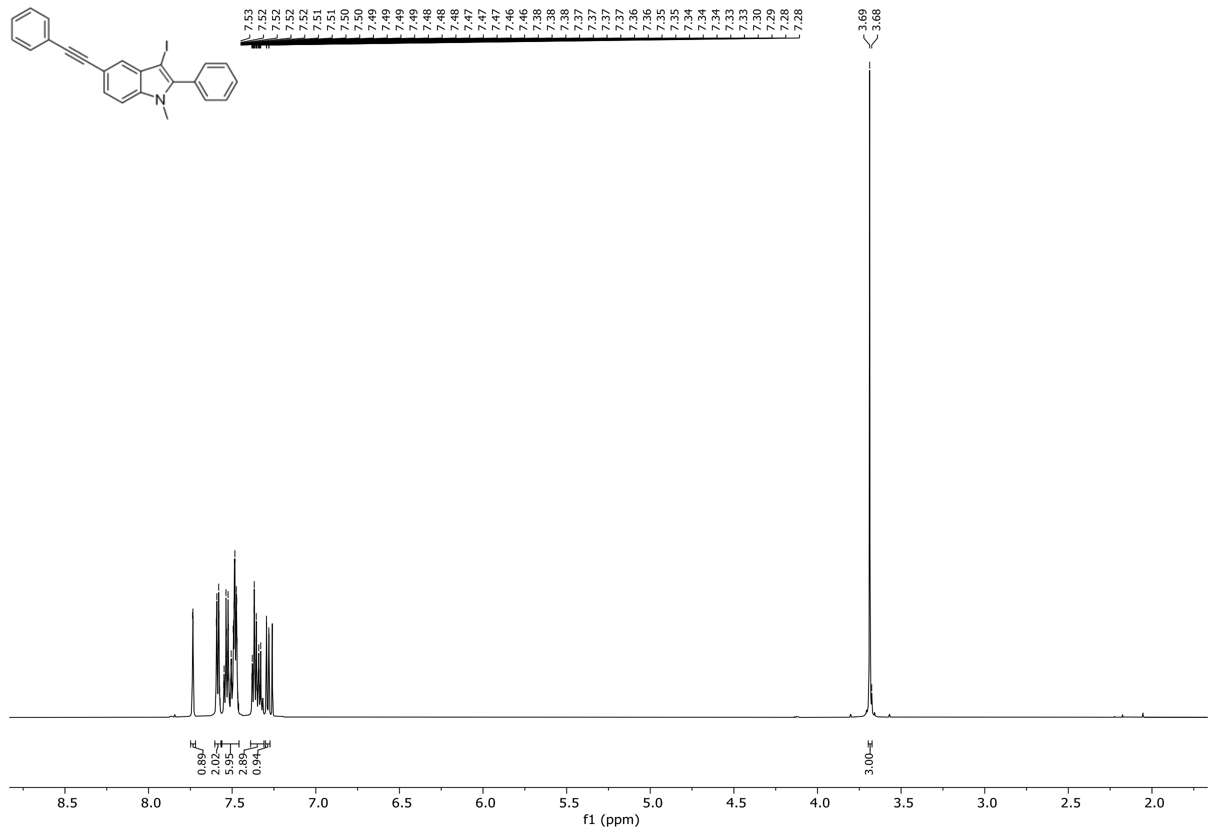

Figure S36. ^{1}H NMR spectrum (300 MHz, recorded in $CDCl_3$, T = 298 K).

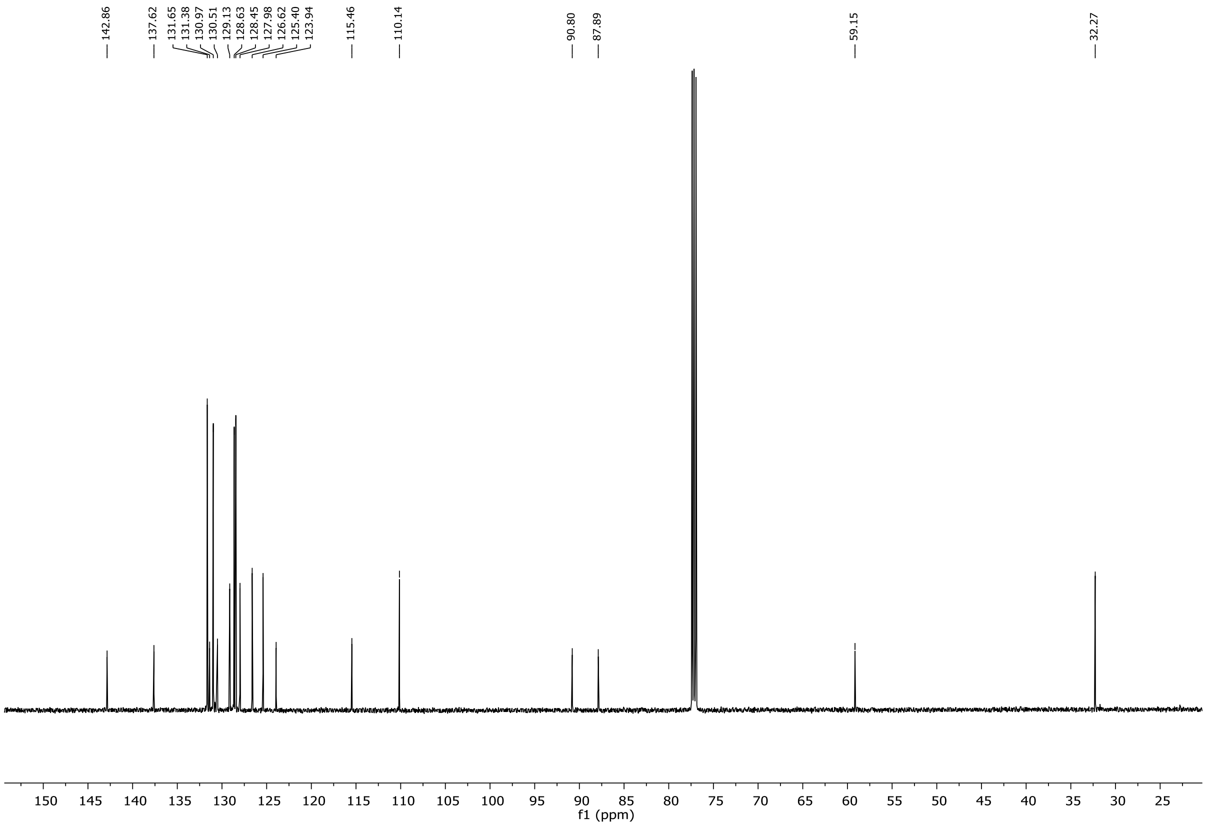

Figure S37. ^{13}C NMR spectrum (75 MHz, recorded in $CDCl_3$, T = 298 K).

4.20. 3-(4-Methoxyphenyl)-1-methyl-2-phenyl-1*H*-indole (8a)

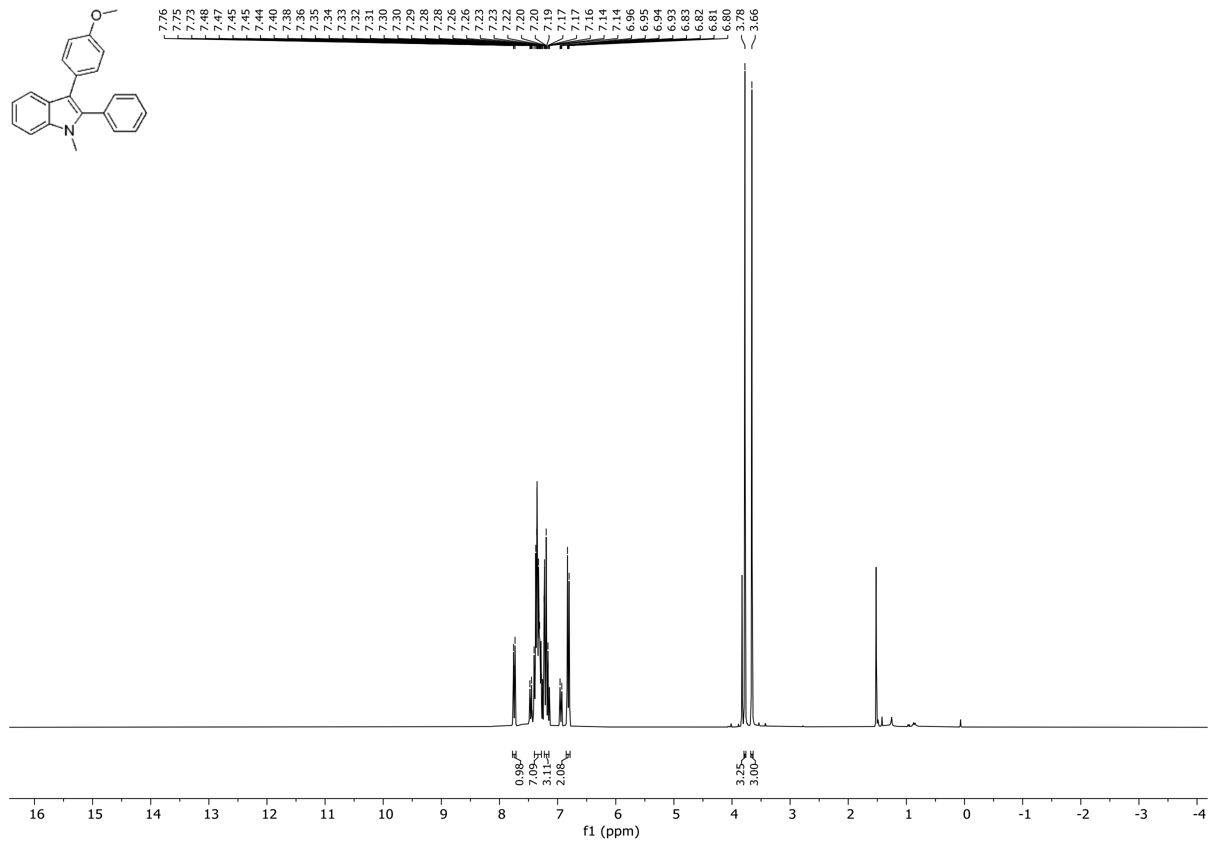

Figure S38. ^{1}H NMR spectrum (300 MHz, recorded in $CDCl_3$, T = 298 K).

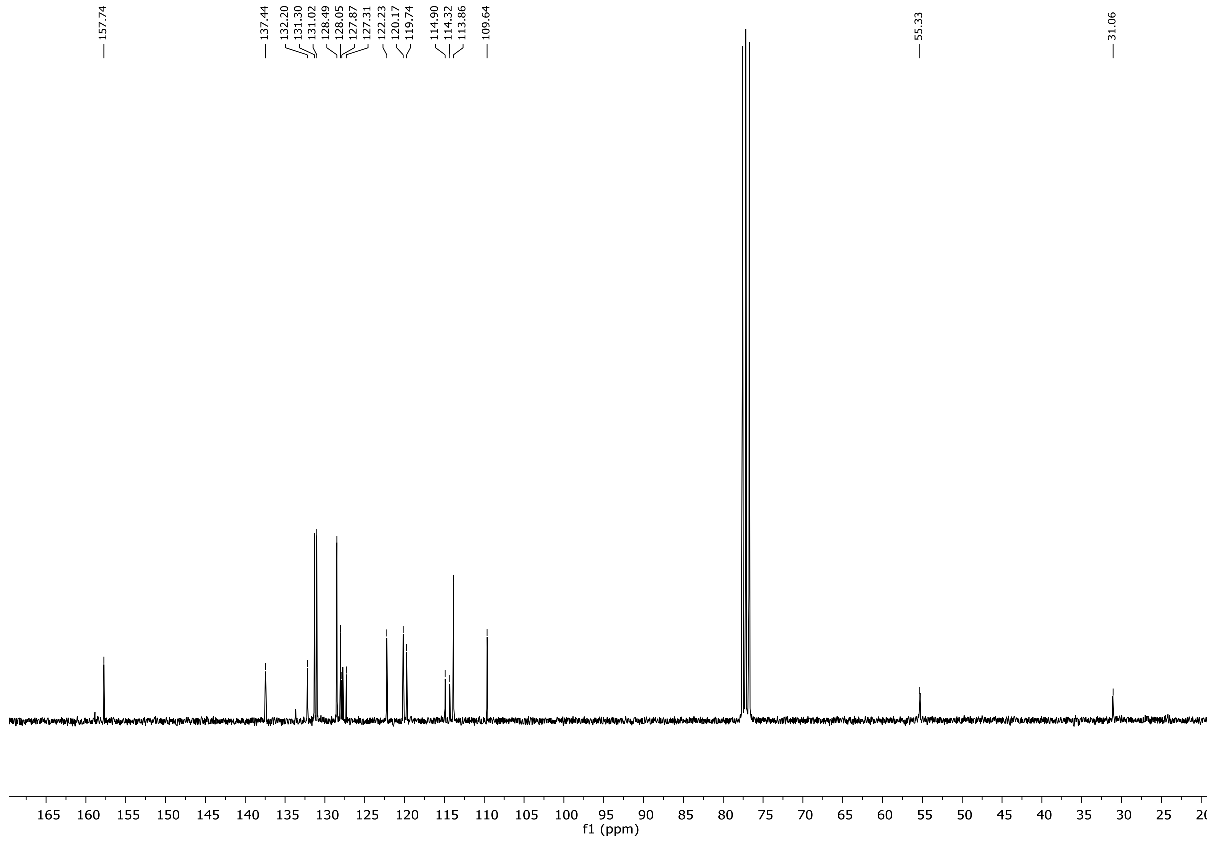

Figure S39. ^{13}C NMR spectrum (75 MHz, recorded in $CDCl_3$, T = 298 K).

4.21. 1-Methyl-2-phenyl-3-(*p*-tolyl)-1*H*-indole (8b)

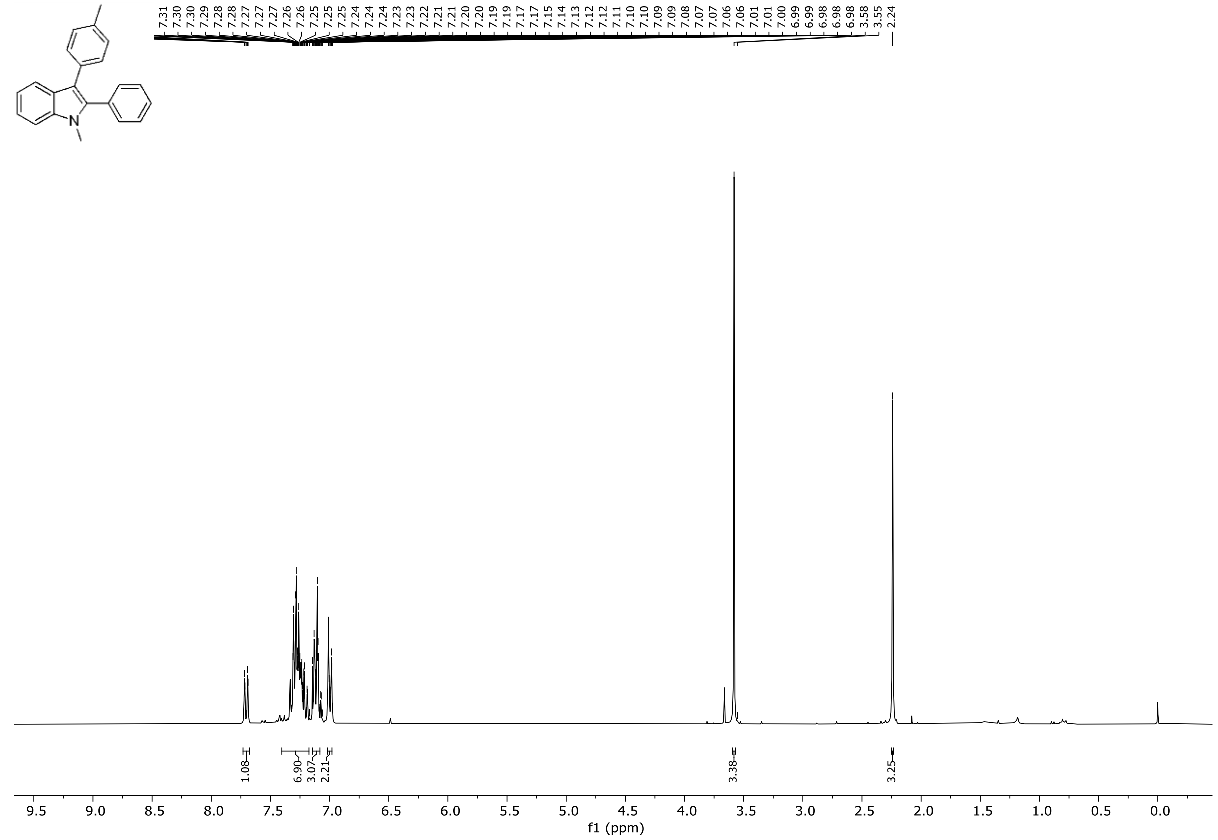

Figure S40. ^{1}H NMR spectrum (300 MHz, recorded in $CDCl_3$, T = 298 K).

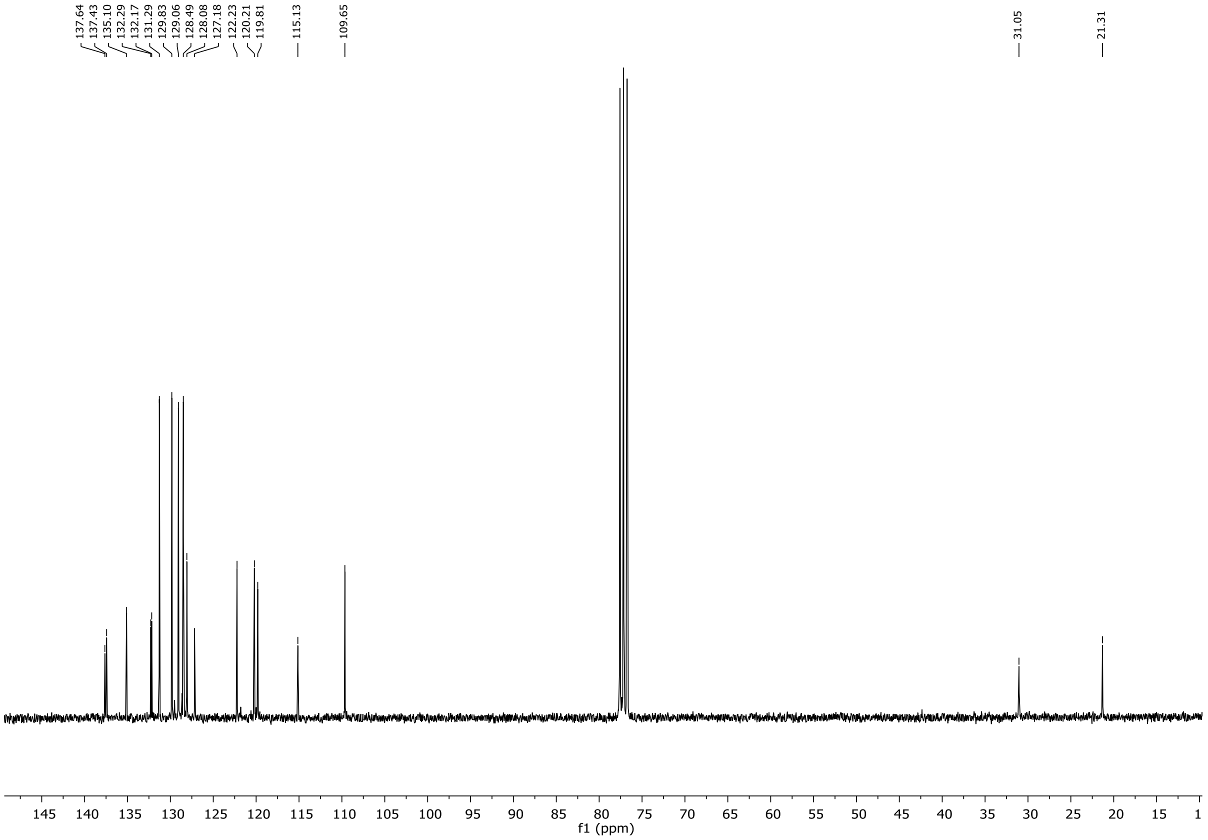

Figure S41. ^{13}C NMR spectrum (75 MHz, recorded in $CDCl_3$, T = 298 K).

4.22. 1-Methyl-2,3-diphenyl-1*H*-indole (8c)

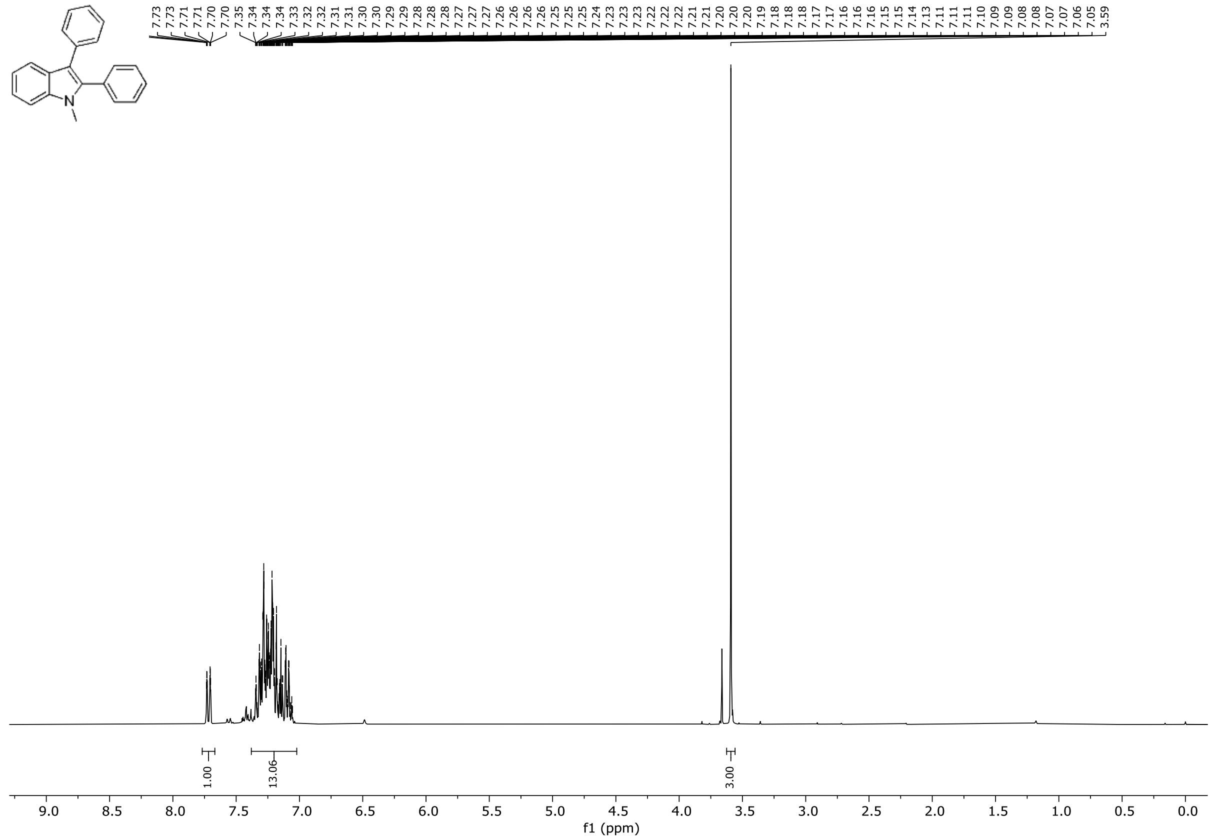

Figure S42. ^{1}H NMR spectrum (300 MHz, recorded in $CDCl_3$, T = 298 K).

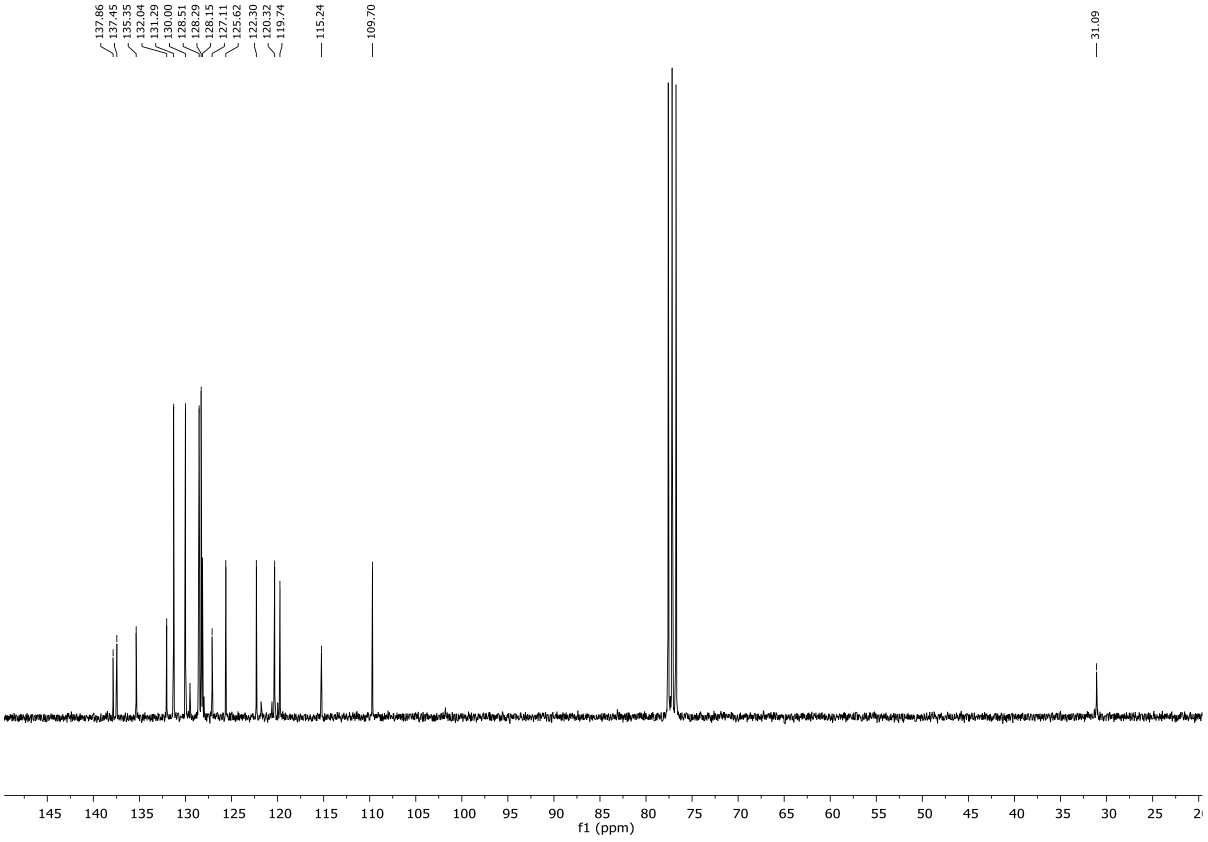

Figure S43. ^{13}C NMR spectrum (75 MHz, recorded in $CDCl_3$, T = 298 K).

4.23. 4-(1-Methyl-2-phenyl-1*H*-indol-3-yl)benzonitrile (8d)

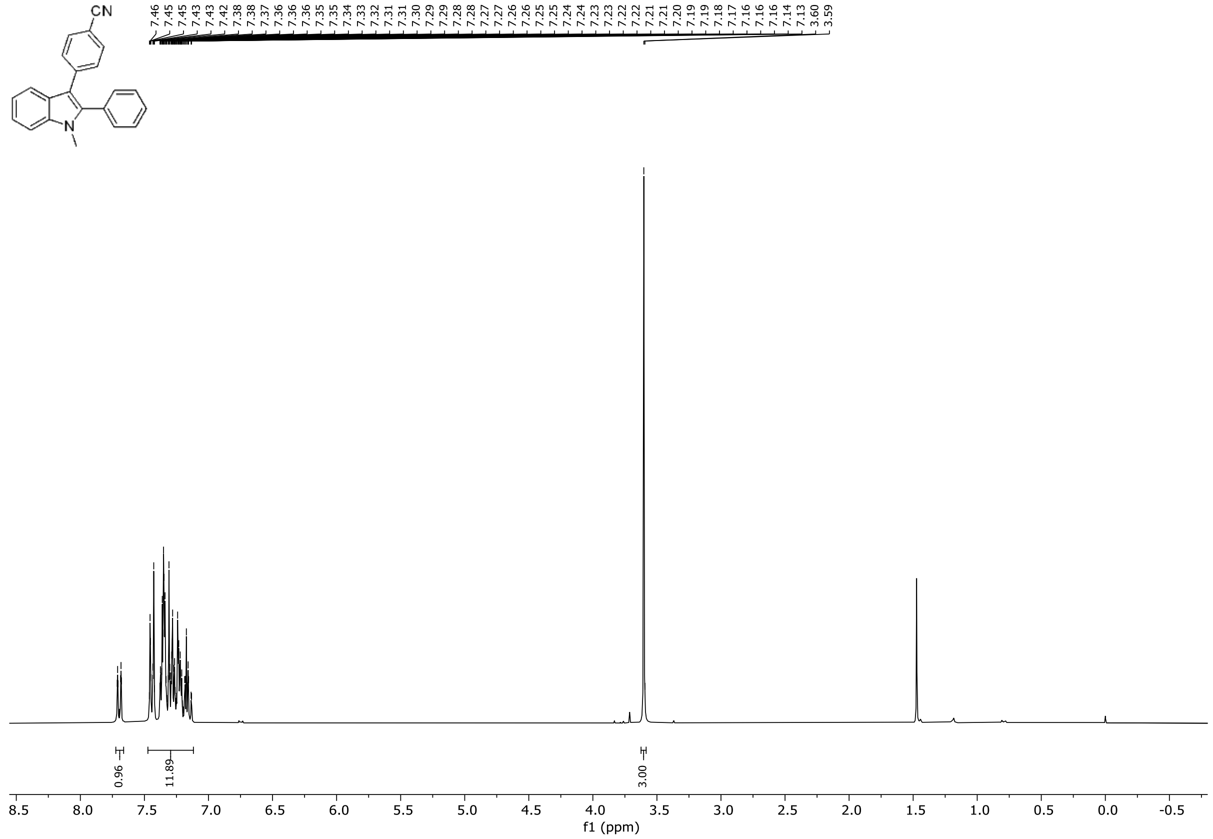

Figure S44. ^{1}H NMR spectrum (300 MHz, recorded in $CDCl_3$, T = 298 K).

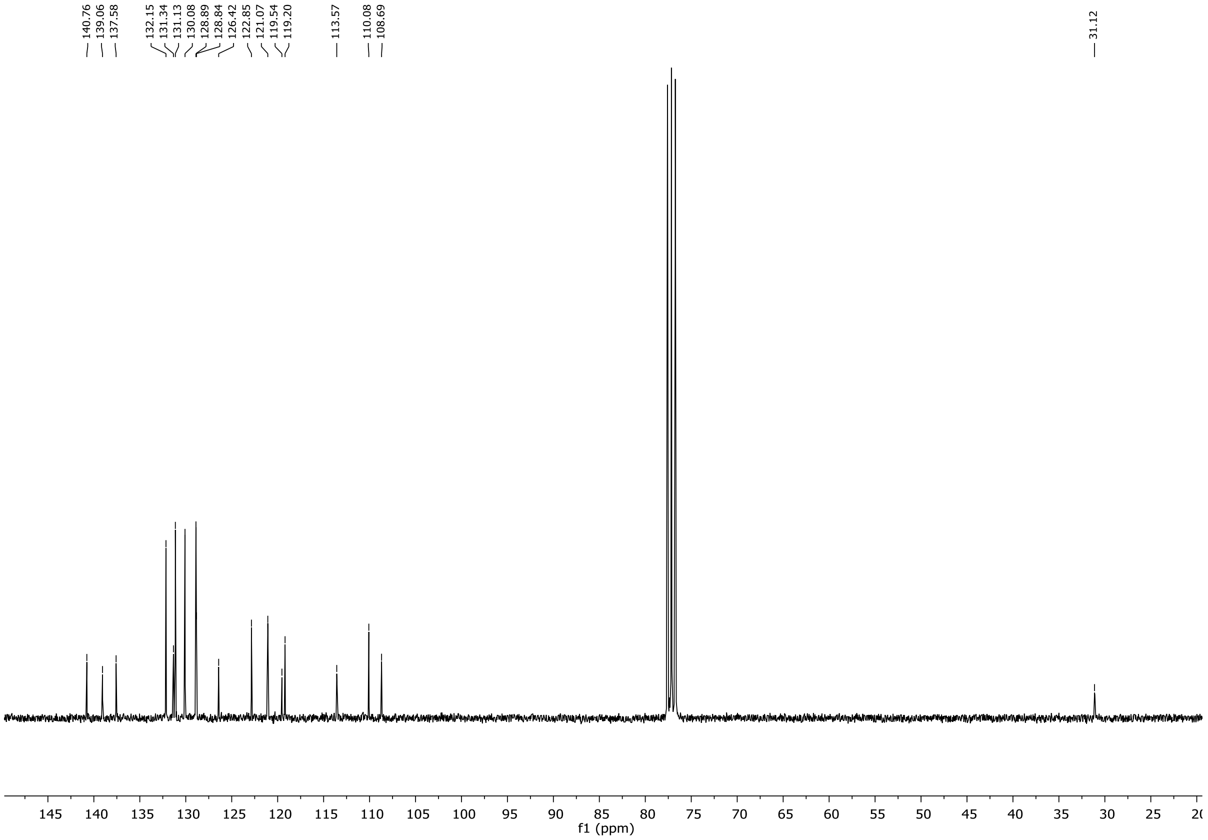

Figure S45. ^{13}C NMR spectrum (75 MHz, recorded in $CDCl_3$, T = 298 K).

5. References

1 J. R. Goerlich, R. Schmutzler, Organophosphorus compounds with tertiary alkyl substituents. VI1: A convenient method for the preparation of Di-1-adamantylphosphine and Di-1-adamyntylchlorophosphine, *Phosphorus Sulfur Silicon Relat. Elem.* **1995**, *102*, 211-215. DOI: https://doi.org/10.1080/10426509508042559

2 N. Zhou, L. Wang, D. W. Thompson, Y. J. T. Zhao, OPE/OPV H-mers: synthesis, electronic properties, and spectroscopic responses to binding with transition metal ions, *Tetrahedron* **2011**, *67*, 125-143. https://doi.org/10.1016/j.tet.2010.11.012

3 M. A. Campo, R. C. Larock, Synthesis of Fluoren-9-ones by the Palladium-Catalyzed Cyclocarbonylation of o-Halobiaryls, *J. Org. Chem.* **2002**, *67*, 5616-5620. https://doi.org/10.1021/jo020140m

4 D. Yue, T. Yao, R. C. Larock, Synthesis of 3-Iodoindoles by the Pd/Cu-Catalyzed Coupling of *N,N*-Dialkyl-2-iodoanilines and Terminal Acetylenes, Followed by Electrophilic Cyclization. *J. Org. Chem.* **2006**, *71*, 62–69. https://doi.org/10.1021/jo051549p

5 A. Sperança, B. Godoi, P. H. Menezes, G. Zeni, *Synlett*, **2013**, *24*, 1125-1132. https://doi.org/10.1055/s-0033-1338427

6 R. W. H. ten Hoedt, G. van Koten, J. G. Noltes, Facile High Yield Synthesis of 1-Methyl-2-p-Tolyl-Indole and Its 3-Iodo Derivative. *Synth. Commun.* **1977**, *7*, 61-69. https://doi.org/10.1080/00397917709410057

7 L. Shi, D. Zhang, R. Lin, C. Zhang, X. Li, N. Jiao, The direct C–H halogenations of indoles. *Tetrahedron Lett.* **2014**, *55*, 2243-2245. https://doi.org/10.1016/j.tetlet.2014.02.071

8 J. Strohmeier, E. von Angerer, Synthesis and Estrogen Receptor Affinity of 2,3-Diarylindoles. *Arch. Pharm.* **1987**, *320*, 407-417. https://doi.org/10.1002/ardp.19873200506

9 F. Brown, F. G. Mann, The mechanism of indole formation from phenacylarylamines. Part II. The stability and reactions of phenacyl-N-alkylarylamines. *J. Chem. Soc.* **1948**, 847-858. https://doi.org/10.1039/JR9480000845

10 M. Miyasaka, A. Fukushima, T. Satoh, K. Hirano, M. Miura, Fluorescent Diarylindoles by Palladium-Catalyzed Direct and Decarboxylative Arylations of Carboxyindoles. *Chem. Eur. J.* **2009**, *15*, 3674-3677. https://doi.org/10.1002/chem.200900098

11 J. Ahmed, S. C. Sau, Sreejyothi P, P. K. Hota, P. K. Vardhanapu, G. Vijaykumar, S. K. Mandal, Direct C–H Arylation of Heteroarenes with Aryl Chlorides by Using an Abnormal N-Heterocyclic-Carbene–Palladium Catalyst. *Eur. J. Org. Chem.* **2017**, 1004-1011. https://doi.org/10.1002/ejoc.201601218
